# Chemotherapeutic Activities of New η^6^-*p*-Cymene Ruthenium(II) and Osmium(II) Complexes with Chelating SS and Tridentate SNS Ligands

**DOI:** 10.3390/molecules29050944

**Published:** 2024-02-21

**Authors:** David O. Ywaya, Halliru Ibrahim, Holger B. Friedrich, Muhammad D. Bala, Lynette Soobramoney, Aliscia Daniels, Moganavelli Singh

**Affiliations:** 1 School of Chemistry and Physics, College of Agriculture, Engineering and Science, University of KwaZulu-Natal, Private Bag X54001, Durban 4000, South Africa; ywayadvd@gmail.com (D.O.Y.); halliruyou@gmail.com (H.I.); bala@ukzn.ac.za (M.D.B.); lynette.komarsammy@gmail.com (L.S.); 2 Nano-Gene and Drug Delivery Group, Discipline of Biochemistry, School of Life Sciences, College of Agriculture, Engineering and Science, University of KwaZulu-Natal, Private Bag X54001, Durban 4000, South Africa; danielsa@ukzn.ac.za (A.D.); singhm1@ukzn.ac.za (M.S.)

**Keywords:** imidazole-2-thiones, ruthenium, osmium, half-sandwich complexes, molecular structures, anti-cancer

## Abstract

A series of new chelating bidentate (SS) alkylimidazole-2-thione-Ru(II)/Os(II) complexes (**3a_i_**, **3a_ii_**, **3a_iii_**, **3b_ii_**/**4a_iii_**, **4b_i_**, **4b_ii_**), and the tridentate (SNS) pyridine-2,6-diylimidazole-2-thione-Ru(II)/Os(II) complexes (**5b_i_**, **5c_iv_**/**6bi**, **6c_i_**, **6c_iv_**) in the forms [M^II^(cym)(L)Cl]PF_6_ and [M^II^(cym)(L)]PF_6_ (M = Ru or Os, cym = η^6^-*p*-cymene, and L = heterocyclic derivatives of thiourea) respectively, were successfully synthesized. Spectroscopic and analytical methods were used to characterize the complexes and their ligands. Solid-state single-crystal X-ray diffraction analyses revealed a “piano-stool” geometry around the Ru(II) or Os(II) centers in the respective complexes. The complexes were investigated for in vitro chemotherapeutic activities against human cervical carcinoma (HeLa) and the non-cancerous cell line (Hek293) using the MTT assay. The compounds **3a_ii_**, **5c_iv_**, **5b_i_**, **4a_iii_**, **6c_i_**, **6c_iv_**, and the reference drug, 5-fluorouracil were found to be selective toward the tumor cells; the compounds **3a_i_**, **3a_iii_**, **3b_ii_**, **4b_i_**, **4b_ii_**, and **6b_i_**, which were found not to be selective between normal and tumor cell lines. The IC_50_ value of the tridentate half-sandwich complex **5b_i_** (86 ± 9 μM) showed comparable anti-proliferative activity with the referenced commercial anti-cancer drug, 5-fluorouracil (87 ± 15 μM). The pincer (SNS) osmium complexes **6c_i_** (36 ± 10 μM) and **6c_iv_** (40 ± 4 μM) were twice as effective as the reference drug 5-fluorouracil at the respective dose concentrations. However, the analogous pincer (SNS) ruthenium complex **5c_iv_** was ineffective and did not show anti-proliferative activity, even at a higher concentration of 147 ± 1 μM. These findings imply that the higher stability of the chelating (SS) and the pincer (SNS) ligand architectures in the complexes improves the biological (anti-proliferative) activity of the complexes by reducing the chance of ligand dissociation under physiological conditions. In general, the pincer (SNS) osmium complexes were found to be more cytotoxic than their ruthenium analogues, suggesting that the anti-proliferative activity of the imidazole-2-thione-Ru/Os complexes depends on the ligand’s spatial coordination, the nature of the metal center, and the charge of the metal complex ions.

## 1. Introduction

Imidazoles are compounds with renowned potency, with many of them being identified as antimicrobial, antithyroid, anti-HIV, and anti-cancer agents [1]. The ease of tuneability of the diazolyl moiety paves the way for functionalization and a wide range of compounds with diverse applications in biological systems, organometallic synthesis, and homogenous catalysis [2,3]. Among such derivatives are the imidazolium salts used to generate corresponding in-situ imidazol-2-ylidenes that can easily be converted to imidazole-2-thiones by direct reaction with elemental sulfur [4,5,6,7,8]. The imidazole-2-thiones exhibit more potent antimicrobial, antifungal [9], and chemotherapeutic activities [10] than their synthons. In organometallic synthesis, the imidazole-2-thiones are analogous to the conventional imidazolylidene as viable ligands and, thus, provide a soft S-donor group for metal coordination [11,12]. This paves the way to complexes with interesting biological activities [3,13,14,15,16,17].

Half-sandwich arene-Ru(II) complexes with various co-ligands have lately attracted intense scientific interest due to their potential as chemotherapeutics agents [18] and promising alternatives to the potent Pt(II) complex, cisplatin, and its oxaliplatin analogues [19,20]. An arene substituent (herein represented by *p*-cymene) on a metal complex has an advantage as a non-labile ligand and is known to stabilize the Ru metal center in its reactive oxidation state of +2 under physiological conditions [21]. Furthermore, experimental evidence shows that the corresponding Ru(II) complexes have kinetic properties similar to Pt(II) complexes [22]. In this vein, efforts have been made by researchers to improve the biological activities of metal complexes by using chelating sulfur—containing ligands to achieve rigidity and limit lability of the complexes [23]. Related studies have demonstrated that complexes containing benzimidazole-thione ligands of the form: [M^II/III^(cym/Cp*)Cl]PF_6_ (M = Ru, Os, Rh, or Ir; cym = η^6^-*p*-cymene and Cp* = η^5^-pentamethylcyclopentadienyl) were active anti-proliferative agents in four cell lines [3]. However, the less kinetically labile Os and Ir derivatives were more potent than their Ru and Rh analogues [3]. Similarly, the design of the imidazole-2-thione ligand framework in this series of new complexes is aimed at taking advantage of its chelating effect and thereby maintaining the +2 oxidation state (with profound physiological effects) on metal (Ru and Os) centers. Hence, the overall object of the current work is to observe their effects on the chemotherapeutic activities of the complexes and the possible activity-structure relationship due to the varying lengths of the spacer and the donor groups in the ligand framework.

Ruthenium and osmium complexes have similar structures and action mechanisms in anti-cancer studies [24,25]. However, osmium complexes have a slower ligand exchange rate and are less reactive than the ruthenium analogues [26,27]. This suggests that osmium complexes may have greater anti-cancer activities as they can reach the target cell unperturbed, with the ligands still coordinated to the osmium center. Related reviews have shown various works on the popularity of NHC-metal complexes based on the coinage metals and those with the PGM group metals, including Ru [28]. However, to the best of our knowledge, such activities based on new imidazole-2-thione Ru or Os complexes are still unreported.

Recently, our group reported on some half-sandwich arene—Ru(II) and Os(II) complexes with *N*,*N*′-chelating ligands, which were more active against cancer cells than the positive control (anti-cancer drug, 5-fluorouracil) [29,30,31]. In further exploring the chemotherapeutic potential of Ru and Os complexes, we herein report for the first time the synthesis, characterization, and in vitro anti-proliferative activities against cervical cancer (HeLa cell line) of a series of new half-sandwich Ru and Os complexes, which are distinguishable by their methylene-, ethylene-, and pyridine-bridged bis(thione) ligands. The chemotherapeutic selectivities of these metal complexes were evaluated using the non-tumor HEK293 (human embryonic kidney) cell line, and a comparison of activities was made with reference to the positive control drug, 5-fluorouracil.

## 2. Results and Discussion

### 2.1. Synthesis and Characterization

**Scheme 1:**
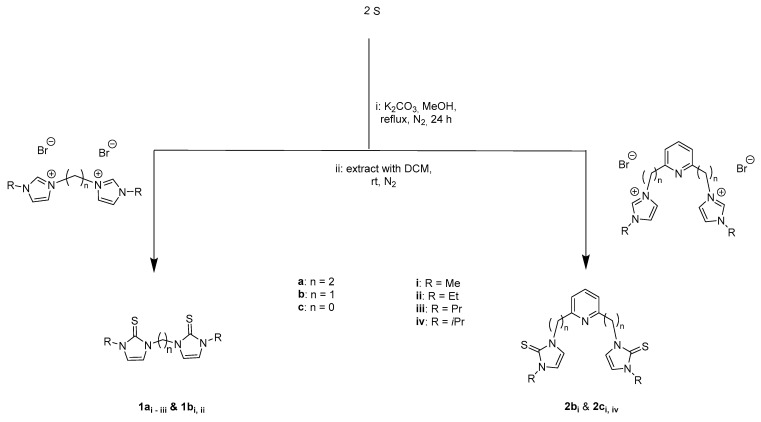
Synthesis of bidentate (SS) alkylimidazole-2-thione ligands, **1a_i–iii_** & **1b_i_**,**_ii_**; and the tridentate (SNS) pyridine-2,6-diylimidazole-2-thione ligands, **2b_i_** & **2c_i_**,**_iv_**.

New alkyl- and pyridine-bridged compounds with the NHC=S moiety were prepared by thionation of alkyl/pyridine-2,6-diyl-bridged imidazolium dibromide in the presence of K_2_CO_3_ according to the literature method (Scheme 1) [4,5,7,8,32,33,34,35]. Both the chelating bidentate (SS) alkylimidazole-2-thione ligands (**1a_i_**, **1a_ii_**, **1a_iii_**, **1b_i_**, **1b_ii_**) and the tridentate (SNS) pyridine-2,6-diylimidazole-2-thione ligands (**2b_i_**, **2c_i_**, **2c_iv_**) were obtained as pale yellow, orange-yellow or colorless solids in moderately high yields, and their identities were confirmed using NMR, FTIR, and HR-MS spectroscopies. The conversion of the imidazolium group into a thione resulted in the loss of the characteristic azolium proton singlet resonance signal at about 10 ppm in the ^1^H-NMR spectra of all the imidazole-2-thione ligands [4,5,32,36]. Further, the observed far downfield resonance signal at around δ 161.6–163.9 ppm in the ^13^C-NMR spectra of all the synthesized imidazole-2-thione ligands and the loss of the imidazolium carbon peak (initially at around 143 ppm) from the derivatized salt, confirmed the formation of the C=S group. In the infrared spectra, three bands consistently appeared in the regions: 1526–1688 cm^−1^, 1070–1190 cm^−1^, and 510–723 cm^−1^, due to mixed vibrations. The bands can be designated as the *υ*(C=C)+*υ*(C=N), *υ*(C-N)+*υ*(C=S), and *υs*(C=S)+*υ*as(C=S) bands, respectively. The HR-MS spectra gave mainly pseudomolecular [M + H]^+^ ions at *m/z* values, within acceptable limits to the calculated values.

**Scheme 2:**
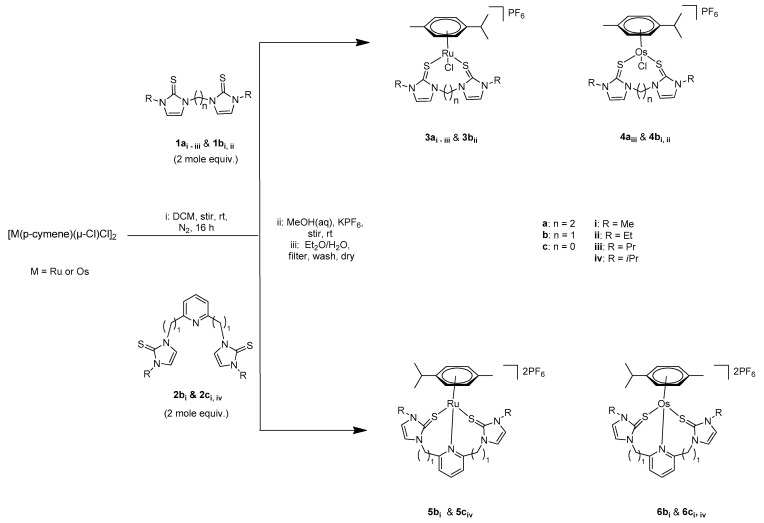
Synthesis of chelating bidentate (SS) alkylimidazole-2-thione-Ru(II)/Os(II) complexes, **3a_i_**, **3a_ii_**, **3a_iii_**, **3b_ii_**/**4a_iii_**, **4b_i,_ 4b_ii_**; and the tridentate (SNS) pyridine-2,6-diylimidazole-2-thione-Ru(II)/Os(II) complexes, **5b_i_**, **5c_iv_**/**6b_i_**, **6c_i_**, **6c_iv_**.

The new brown half-sandwich imidazole-2-thione-ruthenium complexes **3a_i_**, **3a_ii_**, **3a_iii_**, **3b_ii_**, **5b_i_** and **5c_iv_** and their analogous yellow half-sandwich imidazole-2-thione-osmium complexes **4a_iii_**, **4b_i_**, **4b_ii_**, **6b_i_**, **6c_i_**, and **6c_iv_** were obtained by stirring 2 molar equivalents of the respective alkyl/pyridine-2,6-diyl-bridged imidazole-2-thione ligands with [Ru(*p*-cymene)(μ-Cl)Cl]_2_ and [Os(*p*-cymene)(μ-Cl)Cl]_2_ in DCM for 16 h at room temperature [37]. The solution from each system was then poured into an excess aqueous methanolic solution of KPF_6_. This resulted in the formation of either the brown or yellow precipitates of the respective complexes. These were then vacuum-filtered and thoroughly washed with water and ethyl ether. Subsequent drying in vacuo afforded the complexes as air and moisture-stable solids in moderate yields (Scheme 2). All the afforded complexes are soluble in aprotic polar solvents like DMSO, MeCN, DMF, and CH_3_COCH_3_. They were all characterized by NMR and FTIR spectroscopy as well as HR-MS. All complexes had sharp melting points. Suitable single crystals of **3a_i–iii_**, **3b_ii_**, **4a_iii_**, **5c_iv_**, and **6c_i_** were further used to obtain solid-state X-ray crystallographic data for the complexes.

The ^1^H NMR spectra of these complexes showed resonance signals around δ 2.69–2.90 and 5.32–6.37 ppm, due to the isopropyl protons C*H*(CH_3_)_3_ and aromatic protons η^6^-C_6_*H*_4_ of the *p*-cymene moiety in the CD_3_CN solution, respectively. The resonance signals corresponding to the backbone imidazole (olefinic) protons in the imidazole-2-thione ligand moieties appeared at around δ 6.96–7.68 ppm in the spectra of the complexes. The coordination of the ligands to the metal centers caused the signals of the methylene and ethylene protons bridging the thione groups and methylene protons in the lutidyl backbone to become diastereotopic [3,38]. This suggested a twisted conformation in the structure of the complexes, as reported for similar complexes bearing a pincer-like architecture [38]. The ^13^C NMR spectra of all the complexes gave a characteristic slight upfield shift of the resonance signal corresponding to the C=S group to the range δ 152.2–161.6 ppm. This is typical and is attributed to the formation of the S–M bond in the complex molecules [4,34,37,39].

The infrared spectra of the free ligands consisted of *υ*(C=C)+*υ*(C=N) [40,41,42] bands in the regions 1639–1688 cm^−1^ and 1526–1599 cm^−1^. FTIR spectra of the corresponding complexes showed a collapse of the peaks in the former region, with a corresponding slight blue shift in the peaks of the latter region. This suggested a decrease in the delocalization of π-electrons within the ligand’s heterocyclic moiety. This, consequently, led to the weakening of the *υ*(C=N) contribution upon coordination. The appearance of a sharp peak between 820–830 cm^−1^ was ascribed to *υ*(P-F) [29,43,44], and this confirmed the presence of the PF_6_^−^ counter ion in the structure of the complexes. ESI-MS analyses were further used to confirm the identity of the complexes. The ESI-mass spectra of all the complexes (except for **4_aiii_**, **4b_ii_**, **5b_i_**, and **6b_i_**) contained molecular [M−PF_6_]^+^ ions at *m/z* values that match calculated values. The spectra of the complexes **4_aiii_**, **4b_ii_** contained [(M−PF_6_−Cl)]^2+^ ions, while those of **5b_i_** and **6b_i_** showed fragmented ions [(M−PF_6_−2CH_3_)]^2+^. The observed *m/z* values were comparable to the expected calculated data. (See the electronic Supplementary Materials).

**Figure 1:**
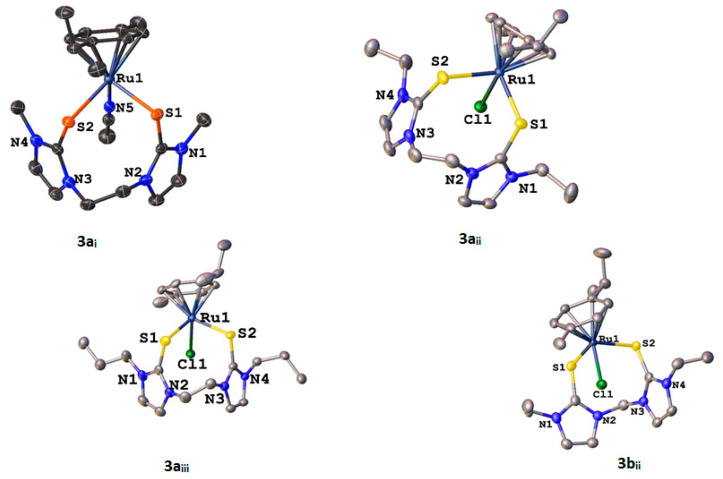
ORTEP plots of the chelating (SS) alkylimidazole-2-thione-Ru(II) complexes, **3a_i_**, **3a_ii_**, **3a_iii_** & **3b_ii_**, with ellipsoids drawn at the 50% probability level. Hydrogen atoms and PF_6_^−^ ions are omitted for clarity.

**Figure 2:**
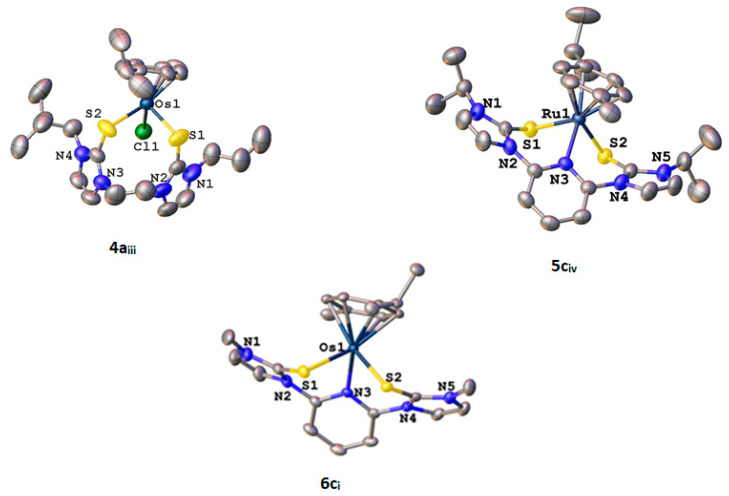
ORTEP plots of the chelating (SS) alkylimidazole-2-thione-Os(II) complex **4a_iii_**; and those of the chelating (SNS) pyridine-2,6-diylimidazole-2-thione-Ru(II)/Os(II) complexes, **5c_iv_** & **6c_i_** with ellipsoids drawn at the 50% probability level. Hydrogen atoms and PF_6_^−^ ions are omitted for clarity.

Single crystals of the complexes **3a_i_**, **3a_ii_**, **3a_iii_**, **3b_ii_**, **4a_iii_**, **5c_iv_**, and **6c_i_** that were suitable for X-ray diffraction analysis were grown via a slow diffusion of diethyl ether into a saturated acetonitrile solution of each of the complexes. Table 1 summarizes the crystallographic data for the compounds and Table 2 gives the selected bond lengths and angles. The traditional piano-stool (half-sandwich) configurations were observed for all complexes (Figure 1 and Figure 2); where the *p*-cymene acted as the seat along with the SS-chelating thione and chlorido or acetonitrile ligands serving as the legs of the stool (Figure 1: **3a_i–iii_**, **3b_ii_**; and Figure 2: **4a_iii_**). The data revealed that during crystallization, the chlorido ligand in **3a_i_** was substituted with an acetonitrile solvent molecule, resulting in a doubly charged complex cation and two PF_6_^−^ counter ions (Figure 1).

As expected, the average Ru-S distance (2.3964 Å) in **5c_iv_** is comparatively shorter than those recorded for the other Ru complexes: **3a_i_** (2.4419 Å), **3a_ii_** (2.4566 Å), **3a_iii_** (2.4562 Å), and **3b_ii_** (2.440 Å) (Table 2). This is attributed to the strong pincer-type bonding mode in the **2c_i_** ligand framework and is within the range of the bond length reported for similar compounds [37]. The Ru-N bond length, 2.0710(16) Å in **3a_i_** (Table 2) formed between the ruthenium and the acetonitrile nitrogen (Figure 1) is shorter than the reported value of 2.044(3) Å in an analogous compound [45]. On the other hand, the Ru-Cl bond lengths in **3a_ii_**, **3a_iii_**, and **3b_ii_** are observed within the range 2.4018(7)–2.4072(4) Å (Table 2), but are shorter than the reported range of 2.423(12)–2.420(19) Å observed in similar compounds [39]. The average C-S bond length in **6c_i_** (1.7030 Å) is also shorter than those in **3a_i-iii_**, **3b_ii_**, **4a_ii_**, **5c_iv_** (Table 2), or as observed in a reported analogue (i.e., 1.7395 Å) [39]. However, the recorded C-S bond length in the complex **6c_i_** is longer than the value of 1.6935 Å observed in other similar compounds [37]. The S-Ru-S bond angle of 84.700(4)° in the pincer (SNS) Ru complex **5c_iv_** is smaller than those observed in the chelating (SS) Ru complexes **3a_i–iii_** and **3b_ii_** (Table 2). In this same vein, the value is also smaller than the previously reported 85.56(3)° [37] and 93.63(10)° [39] for similar compounds.

The average Os-S bond length (2.4483 Å) in the chelating (SS) osmium complex, **4a_iii_** is similar to the Ru-S bond distance (2.4562 Å) in the isostructural ruthenium complex **3a_iii_**. This demonstrates that these complexes are nearly identical in their three-dimensional structure (Figure 1 and Figure 2; and Table 2). The average Os-S bond length (2.4009 Å) in the pincer (SNS) Os complex **6c_i_** is shorter than the recorded 2.4483 Å in the related chelating (SS) Ru complex, **4a_iii_** (Table 2). This is attributed to the strong pincer (SNS) type bonding mode of the ligand, **2c_iv_**. The said value is also shorter than the 2.4401 Å in the previously reported [Os(H)(CO)(PPh_3_){H_2_B(mt)_2_}] [46]. Similarly, the 2.4027(12) Å Os-Cl bond length in **4a_iii_** is shorter than the 2.4110(5) Å reported for the related half-sandwich osmium complex [Os(cym)(L)Cl]PF_6_, where L represents heterocyclic derivatives of thiourea [3]. However, this value is comparable to 2.4030(12) Å for the Ru-Cl bond length in the isostructural ruthenium complex **3a_iii_** (Table 2; and Figure 1 and Figure 2). Similarly, there is minimum variation in the bond length between the metal center and pyridine nitrogen, ranging between the 2.1733(18) Å Os-N bond length in **6c_i_** and the 2.172(2) Å for the corresponding Ru-N in the previously reported isostructural ruthenium complex [Ru(*p*-cymene)(Bmtp)](PF_6_)_2_ [37]. The S-Os-S bond angle of 90.94(6)° in **4a_iii_** is larger than the 86.07(2)° in **6c_i_**. However, this value is comparable to the 91.02(2)° recorded for the S-Ru-S angle in the isostructural ruthenium complex **3a_iii_** (Table 2).

### 2.2. Chemotherapeutic Activities of the Arene-Ru(II)/Os(II) Complexes

Statistical data from the archives of the American Cancer Society indicate that cancer is the second major death-causing disease in the world in recent times. It has accounted for about 17% of reported deaths when compared to other diseases [47]. Although researchers have made many efforts and have provided some efficient anti-cancer drugs to the markets, certain persistent drawbacks like poor solubility, drug resistance, and negative side effects due to the toxic nature of the chemotherapeutic drugs have also continuously fostered the development of novel, promising, and highly effective anti-cancer drugs aimed at bringing an end to the disease [48]. Despite various approaches being practiced in reducing the risk of cancer, there is still a dire need to develop novel treatment strategies that ensure safety as well as efficacy against this life-threatening disease [49].

Certain heterocycles, especially those derived from azoles like imidazole and triazole, have profound potential as effective pharmacological agents [50]. The presence of such heterocycles in the structural motif of drug compounds significantly enhances the bioactive nature of the parent structure. The imidazole moiety is generally found in drugs for fungal infections, but many researchers reported imidazole derivatives as potential anti-cancer agents [51]. This has motivated us and other researchers to explore novel imidazole derivatives, like the imidazole-2-thiones that we present herein as viable nontoxic substitutes to the known chemotherapeutic agents [52]. Thus, we synthesized and assessed the chemotherapeutic potentials of new imidazole-2-thione-Ru/Os complexes on HeLa cancer cell lines and Hek293 cells. The half-sandwich complexes were evaluated alongside 5-fluorouracil (as a positive control) for in vitro cytotoxicity against the cancerous cell lines by the MTT assay method.

The cytotoxicity of the arene-Ru and arene-Os complexes was investigated against HeLa and Hek293 cells. The former cell is a cervical cancer line, while the latter is a non-tumor kidney cell line used to examine tumor selectivity. Compounds **3a_ii_**, **5c_iv_**, **5b_i_**, **4a_iii_**, **6c_i_**, **6c_iv_**, and the reference drug 5-fluorouracil were found to be selective toward the tumor cells, as opposed to compounds **3a_i_**, **3a_iii_**, **3b_ii_**, **4b_i_**, **4b_ii_**, and **6b_i_**, which were found not to be selective between normal cell lines and tumor cell lines (Table 3; entries 1–13). The tridentate half-sandwich complex **5b_i_** (86 ± 9 μM) showed comparable anti-proliferative activity with the referenced commercial anti-cancer drug, 5-fluorouracil (87 ± 15 μM). The pincer (SNS) osmium complexes **6c_i_** (36 ± 10 μM) and **6c_iv_** (40 ± 4 μM) were twice as effective as the reference drug (Table 3; entries 11–13). However, the analogous pincer (SNS) ruthenium complex, **5c_iv,_** was less effective and exhibited a higher IC_50_ value of 147 ± 1 μM (Table 3; entry 9). These findings imply that the higher stability of the chelating (SS) and the pincer (SNS) ligand architectures in the complexes improve the biological (anti-proliferative) activity by rendering ligand dissociation less likely to occur under physiological conditions [21,24]. The pincer (SNS) osmium complexes were more cytotoxic than their ruthenium analogues. These findings suggest that the slower ligand exchange kinetics in these osmium complexes are essential for high cytotoxic activity [3]. These allow for more efficient interactions with biological target molecules. Osmium typically forms coordinative bonds with more inert bonds than in the analogous lighter homolog, ruthenium [27]. Due to their relative inertness and sufficient stability under physiological conditions, osmium complexes are considered exciting alternatives to ruthenium-based anti-cancer agents [26]. However, there was no apparent general pattern in the reactivity of the two metal centers and the isostructural ligand. For instance, the isostructural ruthenium complex **5c_iv_** (147 ± 21 μM) and osmium complex **6c_iv_** (40 ± 4 μM) complexes showed no similar biological activities (Table 3; entries 9 and 12, respectively). The same was also observed with the ruthenium complex **5b_i_** (86 ± 9 μM) and its osmium analogue **6b_i_** (151 ± 12 μM) (Table 3; entries 8 and 10, respectively). This suggested that the effect of the metal center on the anti-proliferative activity depends on the framework in the ligands and their spatial arrangement in coordination to the metal center. Similar findings from a related study have shown that cytotoxicity is affected by the nature of the metal center, with the less kinetically labile Os derivatives being more potent than their Ru counterparts [3]. It was also observed that Os metallodrugs are usually equal to or more potent than their Ru analogues when they contain NO, NN, CN, or SN-bidentate ligands [27]. Our findings, reported herein, further suggest that due to the observed structure-activity relationship in the biological anti-proliferative activities of these compounds, Os metallodrugs are also more potent than their Ru analogues when they contain pincer (SNS) tridentate ligands.

## 3. Material and Methods

### 3.1. General Information

All manipulations were performed under dinitrogen gas using standard Schlenk and vacuum-line techniques. Solvents were purified and degassed by standard procedures before use [53]. Metal precursors: [Ru(*p*-cymene)Cl_2_]_2_, [54,55] [Os(*p*-cymene)Cl_2_]_2_ [56,57] and the imidazole-2-thione ligand precursors: 3,3′-(ethane-1,2-diyl)bis(1-methyl-1*H*-imidazol-3-ium) dibromide, 3,3′-(ethane-1,2-diyl)bis(1-ethyl-1*H*-imidazol-3-ium) dibromide, 3,3′-(ethane-1,2-diyl)bis(1-propyl-1*H*-imidazol-3-ium) dibromide, 3,3′-methylenebis(1-methyl-1*H*-imidazol-3-ium) dibromide, 3,3′-methylenebis(1-ethyl-1*H*-imidazol-3-ium) dibromide [5,36,58,59], 3,3′-(pyridine-2,6-diyl)bis(1-methyl-1*H*-imidazol-3-ium) dibromide, 3,3′-(pyridine-2,6-diyl)bis(1-isopropyl-1*H*-imidazol-3-ium) dibromide, and 3,3′-(pyridine-2,6-diylbis(methylene))bis(1-methyl-1*H*-imidazol-3-ium) dibromide [32,60,61] were all prepared using standard procedures. The imidazole-2-thione ligands; **1a_i_**: 3,3′-(ethane-1,2-diyl)bis(1-methyl-1*H*-imidazole-2(3*H*)-thione) [6], **1b_i_**: 3,3′-methylenebis(1-methyl-1*H*-imidazole-2(3*H*)-thione) [7], **2c_i_**: 3,3′-(pyridine-2,6-diyl)bis(1-methyl-1*H*-imidazole-2(3*H*)-thione) [8] and **2c_iv_**: 3,3′-(pyridine-2,6-diyl)bis(1-isopropyl-1*H*-imidazole-2(3*H*)-thione) [8], were synthesized according to the procedures described in the literature. Other chemicals employed were of analytical grade obtained from Sigma–Aldrich (Johannesburg, South Africa) and used as received. The NMR spectra data were obtained from a Bruker (Karlsruhe, Germany) Top Spin 400 MHz spectrometer using sample solutions in CDCl_3_ for ligands and CD_3_CN for the complexes. The spectra were internally referenced relative to solvent peak(s), and chemical shift values (δ ppm) were recorded with respect to δ = 0 ppm for tetramethylsilane in both ^1^H and ^13^C NMR spectra. Infrared spectra were recorded using an ATR Perkin Elmer (Waltham, MA, USA) Spectrum 100 spectrophotometer operating between 4000 and 400 cm^−1^ in the solid state. High-resolution mass (HR-MS) spectra were recorded using a Waters (Wexford, Ireland) Synapt G2 TOF-MS analyzer by direct ES in the positive mode. The instrument was configured to use Li as the positive ionization specie required for the fragmentation of the molecules in the HR-MS experiments. The melting points were determined with a Stuart Scientific (Microsep, Johannesburg) SMP3 melting point apparatus.

### 3.2. General Procedure for the Synthesis of Imidazole-2-thione Ligands

A modified generic procedure is described herein. In a 100 mL round-bottomed flask fitted with a reflux condenser was placed one of the imidazolium salts: 1,1′-diethyl-3,3-ethylenediimidazolium dibromide, 1,1′-dipropyl-3,3-ethylenediimidazolium dibromide, 1,1′-diethyl-3,3-methylenediimidazolium dibromide, or 3,3′-(pyridine-2,6-diylbis(methylene))bis(1-methyl-1*H*-imidazol-3-ium) dibromide (10 mmol). This was followed by the addition of 2 molar equivalents of sulfur powder (20 mmol), K_2_CO_3_ (2.0 g), and dry methanol (50 mL) as a solvent. The resultant mixture was allowed to reflux for 24 h and then concentrated with a rotary evaporator. The solid obtained was then extracted with DCM (2 × 30 mL) and filtered over a bed of Celite. Removal of all volatiles from the filtrate under reduced pressure afforded the pale yellow, orange-yellow, or colorless crystalline solids of the chelating (SS) ligands **1a_i_**,**1a_ii_**, **1a_iii_**, **1b_i_**, and **1b_ii_** in good to excellent yields (Scheme 1). A similar synthetic route was also employed for the chelating (SNS) ligand analogues **2b_i_**, **2c_i_,** and **2c_iv_**, which bear a pyridine-2,6-diyl spacer as illustrated in Scheme 1.

*3,3′-(ethane-1,2-diyl)bis(1-methyl-1H-imidazole-2(3H)-thione)* (**1a_i_**). Colorless solid. Yield: 1.9 g (75%. m.p.: 195–197 °C). ^1^H NMR (400 MHz, CDCl_3_): δ 3.59 (s, 2NC*H*_3_, 6 H), 4.46 (s, NC*H*_2_-C*H*_2_N, 4 H), 6.58 (d, *J* = 8.2 Hz, imidazole, 4 H). ^13^C NMR (100 MHz, CDCl_3_): δ 35.2 (N*C*H_3_), 45.6 (N*C*H_2_-*C*H_2_N), 117.9 (imidazole), 162.4 (C=S) ppm. FTIR (solid state): *υ*(C=C, C=N) 1639, 1561 cm^−1^; *υ*(C-N, C=S) 1190 cm^−1^; *υ*_s/as_(C=S, C=S) 670, 510 cm^−1^. ESI-HRMS (CH_3_CN): *m/z* found for [M + H]^+^: 255.0738; calculated: 255.0733.

*3,3′-(ethane-1,2-diyl)bis(1-ethyl-1H-imidazole-2(3H)-thione)* (**1a_ii_**). Pale yellow solid. Yield: (1.84 g, 65%. m.p.: 131–133 °C). ^1^H NMR (400 MHz, CDCl_3_): δ 1.34 (t, *J* = 7.3 Hz, 2NCH_2_C*H*_3_, 6H), 4.06 (m, 2NC*H*_2_CH_3_, 4H), 4,48 (s, NC*H*_2_-C*H*_2_N, 4H), 6.58 (d, *J* = 14.0 Hz, imidazole, 4H). ^13^C NMR (100 MHz, CDCl_3_): δ 14.3 (NCH_2_*C*H_3_), 42.9 (N*C*H_2_CH_3_), 45.4 (N*C*H_2_-*C*H_2_N), 115.9, 118.1 (imidazole), 161.6 (C=S). FTIR (solid state): *υ*(=C-H) 3154, 3117, 3084 cm^−1^; *υ*(CH_3_) 2981, 2940 cm^−1^; *υ*(C=C, C=N) 1643, 1563 cm^−1^; *υ*(C-N, C=S) 1177 cm^−1^; *υ*_s/as_(C=S, C=S) 670, 514 cm^−1^. ESI-HRMS (CH_3_CN): *m/z* found for [M + H]^+^: 283.1049; calculated: 283.1051. ESI-HRMS (CH_3_CN): *m/z* found for [M + H]^+^: 283.1049; calculated: 283.1046.

*3,3′-(ethane-1,2-diyl)bis(1-propyl-1H-imidazole-2(3H)-thione)* (**1a_iii_**). Orange solid. Yield: (1.86 g, 60%. m.p.: 91–92 °C). ^1^H NMR (400 MHz, CDCl_3_): δ 0.92 (t *J* = 7.4 Hz, 2NCH_2_CH_2_C*H*_3_, 6H), 1.77 (m, 2NCH_2_C*H*_2_CH_3_, 4H), 3.96 (t, *J* = 7.3 Hz, 2NC*H*_2_CH_2_CH_3_, 4H), 4.49 (s, NC*H*_2_-C*H*_2_N, 4H), 6.54 (d, *J* = 11.2 Hz, imidazole, 4H). ^13^C NMR (100 MHz, CDCl_3_): δ 11.1 (NCH_2_CH_2_*C*H_3_), 22.3 (NCH_2_*C*H_2_CH_3_), 45.3 (N*C*H_2_CH_2_CH_3_), 49.5 (N*C*H_2_-*C*H_2_N) 116.7, 117.9 (imidazole), 161.8 (C=S). FTIR (solid state): *υ*(=C-H) 3163, 3127, 3097 cm^−1^; *υ*(CH_3_) 2959, 2933 cm^−1^; *υ*(C=C, C=N) 1641, 1564, 1526 cm^−1^, *υ*(C-N, C=S) 1179 cm^−1^; *υ*_s/as_(C=S, C=S) 665, 532 cm^−1^. ESI-HRMS (CH_3_CN): *m/z* found for [M + H]^+^: 311.1364; calculated: 311.1364.

*3,3′-(methane-1,1-diyl)bis(1-methyl-1H-imidazole-2(3H)-thione)* (**1b_i_**). Pale yellow solid. Yield: 2.02 g, 84%. m.p.: 194–197 °C). ^1^H NMR (400 MHz, CDCl_3_): δ 3.56 (s, 2NC*H*_3_, 6H), 6.30 (s, NC*H*_2_N, 2H), 6.59 (d, *J* = 2.1 Hz, imidazole, 2H), 7.59 (d, *J* = 2.1, imidazole, 2H) ppm. ^13^C NMR (100 MHz, CDCl_3_): δ 35.3 (N*C*H_3_), 56.2 (N*C*H_2_N), 117.8, 118.7 (imidazole), 163.9 (C=S). FTIR (solid state): *υ*(C=C, C=N) 1654, 1569 cm^−1^; *υ*(C-N, C=S) 1162 cm^−1^; *υ*_s/as_(C=S, C=S) 968, 671, 521 cm^−1^. ESI-HRMS (CH_3_CN): *m/z* found for [M + H]^+^: 241.0592; calculated: 241.0537.

*3,3′-(methane-1,1-diyl)bis(1-ethyl-1H-imidazole-2(3H)-thione)* (**1b_ii_**). Colorless solid. Yield: (1.88 g 70%. m.p.: 175–177 °C). ^1^H NMR (400 MHz, CDCl_3_): δ 1.35 (t, *J* = 7.3 Hz, 2NCH_2_C*H*_3_, 6H), 4.05 (m, 2NC*H*_2_CH_3_, 4H), 6.33 (s, NC*H*_2_N, 2H), 6.62 (d, *J* = 2.3 Hz, imidazole, 2H), 7.64 (d, *J* = 2.3 Hz, imidazole, 2H). ^13^C NMR (100 MHz, CDCl_3_): δ 14.1 (NCH_2_*C*H_3_), 42.9 (N*C*H_2_CH_3_), 55.8 (N*C*H_2_N), 116.0, 116.9 (imidazole), 162.9 (C=S). FTIR (solid state): *υ*(=C-H) 3109, 3080 cm^−1^; *υ*(CH_3_) 2989, 2945 cm^−1^; *υ*(C=C, C=N) 1681, 1566 cm^−1^; *υ*(C-N, C=S) 1070 cm^−1^; *υ*_s/as_(C=S, C=S) 715, 515 cm^−1^. ESI-HRMS (CH_3_CN): *m/z* found for [M + H]^+^: 269.0904; calculated: 269.0850.

*3,3′-(pyridine-2,6-diylbis(methylene))bis(1-methyl-1H-imidazole-2(3H)-thione)* (**2b_i_**). Pale yellow solid. Yield: (2.32 g, 70%. m.p.: 190–193 °C). ^1^H NMR (400 MHz, CDCl_3_): δ 3.59 (s, 2C*H*_3_, 6H), 5.33 (s, NC*H*_2_py, 4 H), 6.67 (d, *J* = 1.7 Hz, imidazole, 2H), 6.81 (d, *J* = 1.4 Hz, imidazole, 2H), 7.2 (d, *J* = 7.8 Hz, py, 2H), 7.59 (t, *J* = 7.7 Hz, py 1H) ppm. ^13^C NMR (100 MHz, CDCl_3_): δ = 35.4 (*C*H_3_), 52.6 (N*C*H_2_py), 117.3, 118.0 (imidazole), 122.1, 138.3, 155.3 (py) 163.1 (C=S). FTIR (solid state): *υ*(C=C, C=N) 1666, 1570 cm^−1^; *υ*(C-N, C=S) 1140, 772 cm^−1^; *υ*_s/as_(C=S, C=S) 723, 527 cm^−1^. ESI-HRMS (CH_3_CN): *m/z* found for [M + H]^+^: 332.1013; calculated: 332.0959.

*3,3′-(pyridine-2,6-diyl)bis(1-methyl-1H-imidazole-2(3H)-thione)* (**2c_i_**). Colorless solid. Yield: (1.91 g 63%. m.p.: 270–273 °C). ^1^H NMR (400 MHz, CDCl_3_): δ 3.66 (s, 2C*H*_3_, 6H), 6.79 (s, imidazole, 2H), 7.48 (s, imidazole, 2H), 8.02 (t, *J* = 8.1 Hz, pyridine, 1H), 8.90 (d, *J* = 8.0 Hz, pyridine, 2H). ^13^C NMR (100 MHz, CDCl_3_): δ 35.3 (*C*H_3_), 115.9 (imidazole), 116.7 (imidazole), 118.7 (pyridine), 140.0 (pyridine), 148.6 (pyridine), 162.9 (C=S) ppm. FTIR (solid state): *υ*(C=C, C=N) 1688, 1577 cm^−1^; *υ*(C-N, C=S) 1084 cm^−1^, *υ*_s/as_(C=S, C=S) 791, 546 cm^−1^. ESI-HRMS (CH_3_CN): *m/z* found for [M + H]^+^: 304.0686; calculated: 304.0691.

*3,3′-(pyridine-2,6-diyl)bis(1-isopropyl-1H-imidazole-2(3H)-thione)* (**2c_iv_**). Colorless solid. Yield: (2.69 g 75%. m.p.: 189–190 °C). ^1^H NMR (400 MHz, CDCl_3_): δ 1.39 (d, *J* = 6.7 Hz, 2(C*H*_3_)_2_CH), 12H), 5.26 (m, 2(CH_3_)_2_C*H*, 2H), 6.86 (s, imidazole, 2H), 7.50 (s, imidazole, 2H), 8.02 (t, *J* = 7.9 Hz, pyridine, 1H), 8.86 (d, *J* = 8.0 Hz, pyridine, 2H). ^13^C NMR (100 MHz, CDCl_3_): δ 21.8 ((*C*H_3_)_2_CH), 48.7 ((CH_3_)_2_*C*H), 113.8 (pyridine), 116.8 (imidazole), 117.6 (imidazole), 139.7 (pyridine), 148.6 (pyridine), 161.6 (C=S) ppm. FTIR (solid state): υ(C=C, C=N) 1669, 1599 cm^−1^; υ(C-N, C=S) 1571, 1127 cm^−1^, υ_s_(C=S)+υ_as_(C=S) 783, 666, 527 cm^−1^. ESI-HRMS (CH_3_CN): *m/z* found for [M + H]^+^: 360.1313; calculated: 360.1317.

### 3.3. General Procedure for the Synthesis of the Chelating Bidentate (SS) Alkylimidazole-2-thione-Ru(II)/Os(II) Complexes and the Tridentate (SNS) Pyridine-2,6-diylimidazole-2-thione-Ru(II)/Os(II) Complexes

[Ru(*p*-cymene)Cl_2_]_2_/[Os(*p*-cymene)Cl_2_]_2_, (0.05 mmol) and alkane or pyridine-bridged organochalcogen ligand (**1a_i–iii_** & **1b_i_**_,**ii**_; or **2b_i_** & **2c_i_**_,**iv**_; 0.1 mmol) were dissolved in dry dichloromethane (DCM; 6 mL) and stirred at room temperature for 16 h, then concentrated at reduced pressure. The crude residue obtained was dissolved in dry methanol (2 mL), and the respective complexes were precipitated by adding an equimolar saturated aqueous solution of KPF_6_. Subsequent filtration, washing with a water/diethyl ether mixture (3 × 2 mL), and drying in vacuo of the precipitate afforded the respective brown or yellow half-sandwich ruthenium or osmium complexes in good to excellent yields (Scheme 2).

*[(η^6^-cymene)Ru(L)Cl]PF_6_* (**3a_i_**: *L* = **1a_i_**). Dark brown solid. Yield: (49.01 mg, 73%. m.p.: 187–188 °C). ^1^H NMR (400 MHz, CDCl_3_): δ 1.32 (d, *J* = 6.9 Hz, (C*H*_3_)_2_CHC_6_H_4_(CH_3_)-*p*), 6H), 2.23 (s, (CH_3_)_2_CHC_6_H_4_(C*H*_3_)-*p*), 3H), 2.88 (m, (CH_3_)_2_C*H*C_6_H_4_(CH_3_)-*p*), 1H), 3.59 (s, 2NCH_3_, 6H), 4.35, 5.23 (m, NC*H*_2_-C*H*_2_N, 4H), 5.70, 5.83 (d, *J* = 6.1 Hz, (CH_3_)_2_CHC_6_*H*_4_(CH_3_)-*p*), 4H), 7.20, 7.25 (d, *J* = 2.0 Hz, imidazole, 4H). ^13^C NMR (100 MHz, CDCl_3_): δ 19.0 (CH_3_)_2_CHC_6_H_4_(*C*H_3_)*-p*), 22.8 (*C*H_3_)_2_CHC_6_H_4_(CH_3_)-*p*), 31.7 (CH_3_)_2_*C*HC_6_H_4_(CH_3_)-*p*), 37.1 (NCH_3_), 48.4 (N*C*H_2_-*C*H_2_), 86.9, 88.7, 105.0, 107.6 (CH_3_)_2_CH*C_6_*H_4_(CH_3_)-*p*), 123.7, 123.8 (imidazole), 154.0 (C=S). FTIR (solid state): *υ*(C=C, C=N) 1567 cm^−1^; *υ*(C-N, C=S) 1200 cm^−1^; *υ*(P-F) 824 cm^−1^; *υ*_s/as_(C=S, C=S) 739, 685, 555 cm^−1^. ESI-HRMS (CH_3_CN): *m/z* found for [M−PF_6_]^+^: 525.0483; calculated: 525.0377.

*[(η^6^-cymene)Ru(L)Cl]PF_6_* (**3a_ii_**: *L* = **1a_ii_**). Dark brown solid. Yield: (46.01 mg, 66%. m.p.: 146–147 °C). ^1^H NMR (400 MHz, CD_3_CN): δ 1.33 (m, 2NCH_2_C*H*_3_, C*H*_3_)_2_CHC_6_H_4_(CH_3_)-*p*, 12H), 2.16 (s, CH_3_)_2_CHC_6_H_4_(C*H*_3_)-*p*, 3H), 2.88 (m, CH_3_)_2_C*H*C_6_H_4_(CH_3_)-*p*, 1H), 3.87–4.38 (m, 2NC*H*_2_CH_3_, NC*H*_2_-C*H*_2_N, 8H), 5.32 (d, *J* = 5.7 Hz, CH_3_)_2_CHC_6_*H*_4_(CH_3_)-*p*, 1H), 5.49 (d, *J* = 5.7 Hz, CH_3_)_2_CHC_6_*H*_4_(CH_3_)-*p*, 1H), 5.69 (d, *J* = 6.0, CH_3_)_2_CHC_6_*H*_4_(CH_3_)-*p*, 1H), 5.82 (d, *J* = 6.0 Hz, CH_3_)_2_CHC_6_*H*_4_(CH_3_)-*p*, 1H), 7.05 (d, *J* = 21.6 Hz, imidazole, 2H), 7.25 (d, *J* = 9.4 Hz, imidazole, 2H). ^13^C NMR (100 MHz, CD_3_CN): δ 14.8 (NCH_2_*C*H_3_), 18.9 (CH_3_)_2_CHC_6_H_4_(*C*H_3_)-*p*), 22.9 (*C*H_3_)_2_CHC_6_H_4_(CH_3_)-*p*), 31.6 (CH_3_)_2_*C*HC_6_H_4_(CH_3_)-*p*), 45.2 (N*C*H_2_CH_3_), 47.7 (N*C*H_2_-*C*H_2_N), 84.5, 85.3, 101.2, 104.0 (CH_3_)_2_CH*C*_6_H_4_(CH_3_)-*p*), 120.7, 122.3 (imidazole), 155.2 (C=S) ppm. FTIR (solid state): *υ*(C=C, C=N) 1565 cm^−1^; *υ*(C-N, C=S) 1150 cm^−1^; *υ*(P-F) 830 cm^−1^, *υ*_s/as_(C=S, C=S) 736, 679, 556 cm^−1^. ESI-HRMS (CH_3_CN): *m/z* found for [M−PF_6_]^+^: 553.0801; calculated: 553.0800.

*[(η^6^-cymene)Ru(L)Cl]PF_6_* (**3a_iii_**: *L* = **1a_iii_**). Dark brown solid. Yield: (51 mg, 70%. m.p.: 111–113 °C). ^1^H NMR (400 MHz, CDCl_3_): δ 0.91 (t, *J* = 7.4 Hz, 2NCH_2_CH_2_C*H*_3_, 6H), 1.32 (d, *J* = 6.9 Hz, (C*H*_3_)_2_CHC_6_H_4_(CH_3_)-*p*), 6H), 1.76 (m, 2NCH_2_C*H*_2_CH_3_, 4H), 2.30 (CH_3_)_2_CHC_6_H_4_(C*H*_3_)-*p*), 3H), 2.82 (m, (CH_3_)_2_C*H*C_6_H_4_(CH_3_)-*p*), 1H), 3.78 (m, NC*H*_2_CH_2_CH_3_, 2H), 4.05 (m, NC*H*_2_CH_2_CH_3_, 2H), 4.47 (m, NC*H*_2_-CH_2_N, 2H), 5.33 (m, NCH_2_-C*H*_2_N, 2H), 2H), 5.87 (d, *J* = 5.8 Hz, (CH_3_)_2_CHC_6_*H*_4_(CH_3_)-*p*), 2H), 6.02 (d, *J* = 5.8 Hz, (CH_3_)_2_CHC_6_*H*_4_(CH_3_)-*p*), 2H), 7.23 (d, *J* = 2.1 Hz, imidazole, 2H), 7.33 (d, *J* = 2.1 Hz, imidazole, 2H). ^13^C NMR (100 MHz, CDCl_3_): δ 11.4 (NCH_2_CH_2_*C*H_3_), 18.8 (CH_3_)_2_CHC_6_H_4_(*C*H_3_)-*p*), 23.1 (NCH_2_*C*H_2_CH_3_), 24.0 (*C*H_3_)_2_CHC_6_H_4_(CH_3_)-*p*), 31.6 (CH_3_)_2_*C*HC_6_H_4_(CH_3_)-*p*), 49.0 (N*C*H_2_CH_2_CH_3_), 51.5 (N*C*H_2_-*C*H_2_N), 79.3, 81.5, 97.8, 100.4 (CH_3_)_2_CH*C*_6_H_4_(CH_3_)-*p*), 122.8, 124.6 (imidazole), 152.3 (C=S). FTIR (solid state): *υ*(C=C, C=N) 1564 cm^−1^; *υ*(C-N, C=S) 1115 cm^−1^, 685; *υ*(P-F) 823 cm^−1^; *υ*_s/as_(C=S, C=S) 738, 555 cm^−1^. ESI-HRMS (CH_3_CN): *m/z* found for [M−PF_6_]^+^: 581.1107; calculated: 581.1003.

*[(η^6^-cymene)Ru(L)Cl]PF_6_* (**3b_ii_**: *L* = **1b_ii_**). Red brown solid. Yield: (35 mg, 51%. m.p.: 199–201 °C). ^1^H NMR (400 MHz CD_3_CN): δ 1.29 (d, *J* = 6.9 Hz, (C*H*_3_)_2_CHC_6_H_4_(CH_3_)-*p*), 6H), 1.39 (t, *J* = 7.8 Hz, 2NC*H*_2_C*H*_3_, 6H), 2.13 (s, (CH_3_)_2_CHC_6_H_4_(C*H*_3_)-*p*), 3H), 2.90 (m, (CH_3_)_2_C*H*C_6_H_4_(CH_3_)-*p*), 1H), 4.08 (m, 2NC*H*_2_CH_3_, 4H), 5.33 (d, *J* = 5.8 Hz, (CH_3_)_2_CHC_6_*H*_4_(CH_3_)-*p*), 2H), 5.51 (d, *J* = 5.8 Hz, (CH_3_)_2_CHC_6_*H*_4_(CH_3_)-*p*), 2H), 6.16 (dd, *J* = 264.3 Hz, NC*H*_2_N, 2H), 7.16 (d, *J* = 2.0 Hz, imidazole, 2H), 7.43 (d, *J* = 2.0 Hz, imidazole, 2H). ^13^C NMR (100 MHz, CD_3_CN): δ 15.1 (NCH_2_*C*H_3_), 19.0 (CH_3_)_2_CHC_6_H_4_(*C*H_3_)-*p*), 22.9 (*C*H_3_)_2_CHC_6_H_4_(CH_3_)-*p*), 31.2 (CH_3_)_2_*C*HC_6_H_4_(CH_3_)-*p*), 45.5 (N*C*H_2_CH_3_), 58.4 (N*C*H_2_N), 84.7, 85.0, 101.3, 104.2 (C*H*_3_)_2_CH*C*_6_H_4_(CH_3_)-*p*), 120.9, 121.7 (imidazole), 158.3 (C=S). FTIR (solid state): *υ*(C=C, C=N) 1571 cm^−1^; *υ*(C-N, C=S) 1059 cm^−1^; *υ*(P-F) 826 cm^−1^; *υ*_s/as_(C=S, C=S) 743, 554 cm^−1^. ESI-HRMS (CH_3_CN): *m/z* found for [M−PF_6_]^+^: 539.0646; calculated: 539.0644.

*[(η^6^-cymene)Ru(L)]PF_6_* (**5c_iv_**: *L* = **2c_iv_**). Dark yellow solid. Yield: (58 mg, 65%. m.p.: 217–219 °C). ^1^H NMR (400 MHz, CDCl_3_): δ (d, *J* = 6.9 Hz, (C*H*_3_)_2_CHC_6_H_4_(CH_3_)-*p*), 6H), 1.50–1.74 (m, (CH_3_)_2_CHC_6_H_4_(C*H*_3_)-*p*), 2NCH(C*H*_3_)_2_, 15H), 2.78 (m, (CH_3_)_2_C*H*C_6_H_4_(CH_3_)-*p*), 1H), 5.22 (2NC*H*(CH_3_)_2_, 2H), 5.32 (d, *J* = 6.1 Hz, (CH_3_)_2_CHC_6_*H*_4_(CH_3_)-*p*), 2H), 5.62 (d, *J* = 6.0 Hz, (CH_3_)_2_CHC_6_*H*_4_(CH_3_)-*p*), 2H), 7.55 (d, *J* = 2.5 Hz, pyridine, 2H), 7.68 (m, imidazole, 4H), 8.35 (t, *J* = 8.1 Hz, pyridine) ppm. ^13^C NMR (100 MHz, CDCl_3_): δ 19.1 (CH_3_)_2_CHC_6_H_4_(*C*H_3_)-*p*), 22.2 (*C*H_3_)_2_CHC_6_H_4_(CH_3_)-*p*), 22.97 (NCH(*C*H_3_)_2_), 32.1 (CH_3_)_2_*C*HC_6_H_4_(CH_3_)-*p*), 53.4 (N*C*H(CH_3_)_2_, 88.5, 89.8, 106.4, 106.6 (CH_3_)_2_CH*C*_6_H_4_(CH_3_)-*p*), 121.0 (pyridine), 122.0, 123.9 (imidazole), 146.2, 149.5 (pyridine), 158.3 (C=S) ppm. FTIR (solid state): *υ*(C=C, C=N) 1609, 1580 cm^−1^; *υ*(C-N, C=S) 1155, 680 cm^−1^; *υ*(P-F) 830 cm^−1^, *υ*_s/as_(C=S, C=S) 739, 555 cm^−1^. ESI-HRMS (CH_3_CN): *m/z* found for [M−PF_6_]^+^: 740.1014; calculated: 740.1019; ESI-HRMS (CH_3_CN): *m/z* found for [M + Li]^+^: 892.0860; calculated: 892.0821.

*[(η^6^-cymene)Ru(L)]PF_6_* (**5b_i_**: *L* = **2b_i_**). Dark brown solid. Yield: (51 mg, 60%. m.p.: 196–198 °C). ^1^H NMR (400 MHz, CDCl_3_): δ 1.27 (d, *J* = 6.9 Hz, (C*H*_3_)_2_CHC_6_H_4_(CH_3_)-*p*), 6H), 1.69(s, (CH_3_)_2_CHC_6_H_4_(C*H*_3_)-*p*), 3H), 2.90 (m, (CH_3_)_2_C*H*C_6_H_4_(CH_3_)-*p*), 1H), 3.57 (s, 2NC*H*_3_, 6H), 5.25 (d, *J* = 14.9 Hz, NCH_2_, 2H), 5.79 (d, *J* = 6.0 Hz, (CH_3_)_2_CHC_6_*H*_4_(CH_3_)-*p*), 2H), 6.06 (d, *J* = 6.0 Hz, (CH_3_)_2_CHC_6_*H*_4_(CH_3_)-*p*), 2H), 6.28 (d, *J* = 14.8 Hz, NCH_2_, 2H), 6.96 (d, *J* = 1.9 Hz, imidazole, 2H), 7.04 (d, *J* = 1.9 Hz, imidazole, 2H), 7.91 (d, *J* = 7.6 Hz, pyridine, 2H), 8.11 (t, *J* = 7.7 Hz, pyridine, 1H). ^13^C NMR (100 MHz, CDCl_3_): δ 18.3 (CH_3_)_2_CHC_6_H_4_(*C*H_3_)-*p*), 22.5, (*C*H_3_)_2_CHC_6_H_4_(CH_3_)-*p*), 31.2 (CH_3_)_2_*C*HC_6_H_4_(CH_3_)-*p*), 36.7 (N*C*H_3_), 57.5 (NCH_3_), 89.5, 105.1, 108.1 (CH_3_)_2_CH*C*_6_H_4_(CH_3_)-*p*), 119.6, 123.9 (imidazole), 129.9, 143.3, 153.0 (pyridine), 161.5 (C=S). FTIR (solid state): *υ*(C=C, C=N) 1569 cm^−1^; *υ*(C-N, C=S) 1145, 699 cm^−1^; *υ*(P-F) 826 cm^−1^, *υ*_s/as_(C=S, C=S) 739, 555 cm^−1^. ESI-HRMS (CH_3_CN): *m/z* found for [M−PF_6_−2CH_3_]^2+^: 684.0386; calculated: 684.0393, for [M−2PF_6_−2CH_3_]^2+^: 538.0687 calculated: 539.0751.

*[(η^6^-cymene)Os(L)Cl]PF_6_* (**4a_iii_**: *L* = **1a_iii_**). Yellow solid. Yield: (55 mg, 68%. m.p.: 125–127 °C). ^1^H NMR (400 MHz, CDCl_3_): δ 0.91 (t, *J* = 7.4 Hz, 2NCH_2_CH_2_C*H*_3_, 6H), 1.32 (d, *J* = 6.9 Hz, (C*H*_3_)_2_CHC_6_H_4_(CH_3_)-*p*), 6H), 1.76 (m, 2NCH_2_C*H*_2_CH_3_, 4H), 2.30 (CH_3_)_2_CH*C*_6_H_4_(C*H*_3_)-*p*), 3H), 2.82 (m, (CH_3_)_2_C*H*C_6_H_4_(CH_3_)-*p*), 1H), 3.78 (m, NC*H*_2_CH_2_CH_3_, 2H), 4.05 (m, NC*H*_2_CH_2_CH_3_, 2H), 4.47 (m, NC*H*_2_-CH_2_N, 2H), 5.33 (m, NCH_2_-C*H*_2_N, 2H), 2H), 5.87 (d, *J* = 5.8 Hz, (CH_3_)_2_CHC_6_*H*_4_(CH_3_)-*p*), 2H), 6.02 (d, *J* = 5.8 Hz, (CH_3_)_2_CHC_6_*H*_4_(CH_3_)-*p*), 2H), 7.23 (d, *J* = 2.1 Hz, imidazole, 2H), 7.33 (d, *J* = 2.1 Hz, imidazole, 2H). ^13^C NMR (100 MHz, CDCl_3_): δ 11.4 (NCH_2_CH_2_*C*H_3_), 18.8 (CH_3_)_2_CHC_6_H_4_(*C*H_3_)-*p*), 23.1 (NCH_2_*C*H_2_CH_3_), 24.0 (*C*H_3_)_2_CHC_6_H_4_(CH_3_)-*p*), 31.6 (CH_3_)_2_*C*HC_6_H_4_(CH_3_)-*p*), 49.0 (N*C*H_2_CH_2_CH_3_), 51.5 (N*C*H_2_-*C*H_2_N), 79.3, 81.5, 97.8, 100.4 (CH_3_)_2_CH*C*_6_H_4_(CH_3_)-*p*), 122.8, 124.6 (imidazole), 152.3 (C=S). FTIR (solid state): *υ*(C=C, C=N) 1566 cm^−1^; *υ*(C-N, C=S) 1113, 691 cm^−1^; *υ*(P-F) 827 cm^−1^; *υ*_s/as_(C=S, C=S) 739, 556 cm^−1^. ESI-HRMS (CH_3_CN): *m/z* found for [M−PF_6_−Cl]^+^: 635.1862; calculated: 635.4136.

*[(η^6^-cymene)Os(L)Cl]PF_6_* (**4b_i_**: *L* = **1b_i_**). Yellow solid. Yield: (47 mg, 63%. m.p.: 158–160 °C). ^1^H NMR (400 MHz CDCl_3_): δ 1.29 (d, *J* = 6.9 Hz, (C*H*_3_)_2_CHC_6_H_4_(CH_3_)-*p*), 6H), 2.29 (s, (CH_3_)_2_CHC_6_H_4_(C*H*_3_)-*p*), 3H), 2.87 (m, (CH_3_)_2_C*H*C_6_H_4_(CH_3_)-*p*), 1H), 3.73 (s, 2NC*H*_3_, 6H), 5.84 (d, *J* = 5.7 Hz, (CH_3_)_2_CHC_6_*H*_4_(CH_3_)-*p*), 2H), 6.01 (d, *J* = 5.7 Hz, (CH_3_)_2_CHC_6_*H*_4_(CH_3_)-*p*), 2H), 6.01 (d, *J* = 5.7 Hz, (CH_3_)_2_CHC_6_*H*_4_(CH_3_)-*p*), 2H), 6.15 (d, *J* = 14.1 Hz, NC*H*_2_N, 1H), 6.57 (d, *J* = 14.0 Hz, NC*H*_2_N, 1H), 7.34 (d, *J* = 1.8 Hz, imidazole, 2H), 7.63 (d, *J* = 1.8 Hz, imidazole, 2H). ^13^C NMR (100 MHz, CDCl_3_): δ 18.8 (CH_3_)_2_CHC_6_H_4_(*C*H_3_)-*p*), 23.1 (*C*H_3_)_2_CHC_6_H_4_(CH_3_)-*p*), 31.6 (C*H*_3_)_2_*C*HC_6_H_4_(CH_3_)-*p*), 37.6 (N*C*H_3_), 59.7 (N*C*H_2_N), 78.9, 80.7, 97.8, 101.0 (C*H*_3_)_2_CH*C*_6_H_4_(CH_3_)-*p*), 122.2, 125.1 (imidazole), 156.5 (C=S) ppm. FTIR (solid state): *υ*(C=C, C=N) 1572 cm^−1^; *υ*(C-N, C=S) 1098 cm^−1^; *υ*(P-F) 820 cm^−1^; *υ*_s/as_(C=S, C=S) 751, 555 cm^−1^. ESI-HRMS (CH_3_CN): *m/z* found for [M−PF_6_]^+^: 601.0878; calculated: 601.0902.

*[(η^6^-cymene)Os(L)Cl]PF_6_* (**4b_ii_**: *L* = **1b_ii_**). Yellow solid. Yield: (43 mg, 56%. m.p.: 161–163 °C). ^1^H NMR (400 MHz, CDCl_3_): δ 1.28–1.41 (m, 2NCH_2_C*H*_3_, (C*H*_3_)_2_CHC_6_H_4_(CH_3_)-*p*), 12H), 2.27 (s (CH_3_)_2_CHC_6_H_4_(C*H*_3_)-*p*), 3H), 2.84 (m, (CH_3_)_2_C*H*C_6_H_4_(CH_3_)-*p*), 1H), 4.00–4.26 (m. 2NC*H*_2_CH_3_, 4H), 5.84–5.88 (m, (CH_3_)_2_CHC_6_*H*_4_(CH_3_)-*p*), 2H), 6.02 (d, *J* = 5.7 Hz, (CH_3_)_2_CHC_6_*H*_4_(CH_3_)-*p*), 2H), 6.16 (d, *J* = 14.0 Hz, NC*H*_2_N, 1H), 6.58 (d, *J* = 14.1 Hz, NC*H*_2_N, 1H), 7.18 (d, *J* = 1.9 Hz, imidazole, 1H), 7.41 (d, *J* = 1.9 Hz, imidazole, 1H), 7.50 (d, *J* = 2.0 Hz, imidazole, 1H), 7.68 (d, *J* = 1.9 Hz, imidazole, 1H). ^13^C NMR (100 MHz, CDCl_3_): δ 15.6 (NCH_2_*C*H_3_), 18.8 (CH_3_)_2_CHC_6_H_4_(*C*H_3_)-*p*), 23.0 (*C*H_3_)_2_CHC_6_H_4_(CH_3_)-*p*), 31.5 (CH_3_)_2_*C*HC_6_H_4_(CH_3_)-*p*), 46.2 (N*C*H_2_CH_3_), 59.6 (N*C*H_2_N), 79.3, 81.0, 97.8, 100.8 (CH_3_)_2_CH*C*_6_H_4_(CH_3_)-*p*), 122.8, 123.5 (imidazole), 155.5 (C=S). FTIR (solid state): *υ*(C=C, C=N) 1572 cm^−1^; *υ*(C-N, C=S) 1090 cm^−1^; *υ*(P-F) 823 cm^−1^; *υ*_s/as_(C=S, C=S) 769, 555 cm^−1^. ESI-HRMS (CH_3_CN): *m/z* found for [(M−PF_6_−Cl)]^2+^: 593.1472; calculated: 594.1527.

*[(η^6^-cymene)Os(L)]PF_6_* (**6c_i_**: *L* = **2c_i_**). Yellow solid. Yield: (62 mg, 68%. m.p.: 259–260 °C). ^1^H NMR (400 MHz, CDCl_3_): δ 1.20 (d, *J* = 6.9 Hz, (C*H*_3_)_2_CHC_6_H_4_(CH_3_)-*p*), 6H), 1.83 (CH_3_)_2_CHC_6_H_4_(C*H*_3_)-*p*), 3H), 2.69 (m, (CH_3_)_2_C*H*C_6_H_4_(CH_3_)-*p*), 1H), 3.88 (s, 2NC*H*_3_, 6H), 5.76 (d, *J* = 5.8 Hz, (CH_3_)_2_CHC_6_*H*_4_(CH_3_)-*p*), 2H), 6.00 (d, *J* = 5.8 Hz, (CH_3_)_2_CHC_6_*H*_4_(CH_3_)-*p*), 2H), 7.39 (d, *J* = 2.4 Hz, imidazole, 2H), 7.60 (d, *J* = 2.4 Hz, imidazole, 2H), 7.66 (d, *J* = 7.9 Hz, pyridine, 2H), 8.25 (t, *J* = 8.2 Hz, pyridine, 1H). ^13^C NMR (100 MHz, CDCl_3_): δ 19.2 (CH_3_)_2_CHC_6_H_4_(*C*H_3_)-*p*), 23.0 (*C*H_3_)_2_CHC_6_H_4_(CH_3_)-*p*), 32.1 (CH_3_)_2_*C*HC_6_H_4_(CH_3_)-*p*), 37.2 (N*C*H_3_), 80.6, 81.9, 98.8, 99.4 (CH_3_)_2_CH*C*_6_H_4_(CH_3_)-*p*), 120.9, 123.5 (imidazole), 125.8, 146.6, 149.3 (pyridine), 159.2 (C=S). FTIR (solid state): *υ*(C=C, C=N) 1607 cm^−1^; *υ*(C-N, C=S) 1158, 1096 cm^−1^; *υ*(P-F) 822 cm^−1^; *υ*_s/as_(C=S, C=S) 722, 554 cm^−1^. ESI-HRMS (CH_3_CN): *m/z* found for [M−PF_6_]^+^: 772.0970; calculated: 774.0964.

*[(η^6^-cymene)Os(L)]PF_6_* (**6c_iv_**: *L* = **2c_iv_**). Yellow solid. Yield: (60 mg, 62%. m.p.: 209–210 °C). ^1^H NMR (400 MHz, CDCl_3_): δ 1.20 (d, *J* = 6.9 Hz, (C*H*_3_)_2_CHC_6_H_4_(CH_3_)-*p*), 6H), 1.48 (d, *J* = 6.7 Hz, NCH(CH_3_)_2_, 6H), 1.69 (d, *J* = 6.7 Hz, NCH(CH_3_)_2_, 6H), 1.83 (s, (CH_3_)_2_CHC_6_H_4_(C*H*_3_)-*p*), 3H), 2.69 (m, (CH_3_)_2_C*H*C_6_H_4_(CH_3_)-*p*), 1H), 5.11 (m, 2NC*H*(CH_3_)_2_, 2H), 5.70(d, *J* = 5.7 Hz, (CH_3_)_2_CHC_6_*H*_4_(CH_3_)-*p*), 2H), 5.92 (d, *J* = 5.7 Hz, (CH_3_)_2_CHC_6_*H*_4_(CH_3_)-*p*), 2H), 7.52 (d, *J* = 2.4 Hz, pyridine, 2H), 7.66 (m, imidazole, 4H), 8.27 (t, *J* = 8.2 Hz, pyridine). ^13^C NMR (100 MHz, CDCl_3_): δ 19.3 (CH_3_)_2_CHC_6_H_4_(*C*H_3_)-*p*), 22.2 (*C*H_3_)_2_CHC_6_H_4_(CH_3_)-*p*), 22.9 (NCH(*C*H_3_)_2_, 32.0 (CH_3_)_2_*C*HC_6_H_4_(CH_3_)-*p*), 53.4 (N*C*H(CH_3_)_2_, 80.8, 82.2, 98.5, 99.5 (CH_3_)_2_CH*C*_6_H_4_(CH_3_)-*p*), 121.2 (pyridine), 122.2, 123.9 (imidazole), 146.5, 149.2 (pyridine), 157.9 (C=S). FTIR (solid state): *υ*(C=C, C=N) 1609, 1580 cm^−1^; *υ*(C-N, C=S) 1153, 734 cm^−1^; *υ*(P-F) 827 cm^−1^, *υ*_s/as_(C=S, C=S) 680, 556 cm^−1^. ESI-HRMS (CH_3_CN): *m/z* found for [M−PF_6_]^+^: 830.1597; calculated: 830.1590.

*[(η^6^-cymene)Os(L)]PF_6_* (**6b_i_**: *L* = **2b_i_**). Yellow solid. Yield: (57 mg, 60%. m.p.: 275–277 °C). ^1^H NMR (400 MHz, CDCl_3_): δ 1.27 (d, *J* = 6.8 Hz, (C*H*_3_)_2_CHC_6_H_4_(CH_3_)-*p*), 1.78 (s, (CH_3_)_2_CHC_6_H_4_(C*H*_3_)-*p*), 3H), 2.83 (m, (CH_3_)_2_C*H*C_6_H_4_(CH_3_)-*p*), 1H), 3.59 (s, 2NC*H*_3_, 6H), 5.22 (d, *J* = 14.9 Hz, NC*H*_2_, 2H), 6.07 (d, *J* = 5.6 Hz, (CH_3_)_2_CHC_6_*H*_4_(CH_3_)-*p*), 2H), 6.19 (d, *J* = 14.9 Hz, NCH_2_, 2H), 6.37 (d, *J* = 5.6 Hz, (CH_3_)_2_CHC_6_*H*_4_(CH_3_)-*p*), 2H), 6.99 (d, *J* = 1.7 Hz, imidazole, 2H), 7.07 (d, *J* = 1.8 Hz, imidazole, 2H), 7.88 (d, *J* = 7.7 Hz, pyridine, 2H), 8.02 (t, *J* = 7.7 Hz, pyridine, 1H). ^13^C NMR (100 MHz, CDCl_3_): δ = 18.1 (CH_3_)_2_CHC_6_H_4_(*C*H_3_)-*p*), 22.6 (*C*H_3_)_2_CHC_6_H_4_(CH_3_)-*p*), 31.1 (CH_3_)_2_*C*HC_6_H_4_(CH_3_)-*p*), 36.8 (N*C*H_3_), 58.3 (N*C*H_2_), 81.8, 97.5, 101.8, 120.0 (CH_3_)_2_CH*C*_6_H_4_(CH_3_)-*p*), 123.6, 129.8 (imidazole), 139.5, 143.5, 152.3 pyridine, 161.6 (C=S). FTIR (solid state): *υ*(C=C, C=N) 1606 cm^−1^; *υ*(C-N, C=S) 1162 cm^−1^; *υ*(P-F) 823 cm^−1^, *υ*_s/as_(C=S, C=S) 722, 555 cm^−1^. ESI-HRMS (CH_3_CN): *m/z* found for [M−PF−2CH_3_]^2+^: 774.1000; calculated: 774.0964.

### 3.4. X-ray Crystallography

X-ray diffraction data on single crystals of **3a_i–iii_**, **3b_ii_**, **5c_iv,_** and **6c_i_** were collected with a Bruker APEX-II CCD diffractometer equipped with graphite-monochromated MoKα radiation (λ = 0.71073 Å). The crystals were kept at 149.99 K during data collection using Olex2 [62]. The structures were solved using Intrinsic Phasing with the SHELXT [63] structure solution program and refined with the SHELXL [64] refinement package using Least Squares minimization. The crystal data and structure refinement information obtained are summarized in Table 1. Crystallographic data for the structures in this article have been deposited with the Cambridge Crystallographic Data Centre with CCDC numbers for compounds **3ai**–**6ci** of 2302988-2302990 and 2321930-2321933. The data can be obtained free of charge at http://www.ccdc.cam.ac.uk/conts/retrieving.html, accessed on 16 February 2024 (or from the Cambridge Crystallographic Data Centre, 12 Union Road Cambridge CB2 1EZ, UK; Fax: +44-1223/336-033; E-mail: deposit@ccdc.cam.ac.uk).

### 3.5. In Vitro Anti-Cancer Activity

#### 3.5.1. Chemicals

Phosphate-buffered saline tablets (PBS), 3-(4,5-dimethylthiazol-2-yl)-2.5-diphenyltetrazolium bromide (MTT), and DMSO were obtained from Merck (Darmstadt, Germany). The human embryonic kidney (HEK293) and cervical carcinoma (HeLa) cell lines were sourced from the American Type Culture Collection (ATCC) (Manassas, VA, USA). Sterile cell culture plasticware was procured from Nest Biotechnologies (Wuxi, China), and the Eagles minimum essential medium (EMEM), trypsin-versene, antibiotics (penicillin (5000 U/mL)/streptomycin (5000 g/mL), were supplied by Lonza BioWhittaker (Walkersville, MD, USA). Gamma-irradiated fetal bovine serum (FBS) was obtained from Cytiva Europe GmbH, Vienna, Austria. Ultrapure deionized 18 MΩ water (Milli-Q50, Merck Life Science, Johannesburg, South Africa) was used throughout.

#### 3.5.2. MTT Cell Viability Assay

The cytotoxicity of the compounds was examined in the HeLa and HEK293 cells using the MTT assay [65]. Cells were trypsinized, seeded into clear 96 well plates, and incubated at 37 °C overnight to allow cells to attach. The cells were then prepared by replacing the growth medium with a fresh complete medium (EMEM+10% FBS+ 1% Antibiotics). The samples were then added as a DMSO/H_2_O solution to the cells at various concentrations and incubated at 37 °C for 48 h. After that, the growth medium was aspirated and replaced with the medium (0.1 mL) and MTT solution (0.01 mL: 5 mg/mL in PBS), and the cells were incubated for a further 4 h. The medium was then removed and replaced with DMSO (0.1 mL). Absorbance measurements were then taken at 570 nm using a Mindray MR-96A microplate reader (Vacutec, Hamburg, Germany).

## 4. Conclusions

A new series of half-sandwich ruthenium and osmium complexes with alkyl or pyridine-2,6-diyl bridged imidazole-2-thione ligands was synthesized and evaluated for their chemotherapeutic activities against the human cervical cancer cell line (HeLa) and the non-cancerous line (Hek293). The findings showed that the activities of the complexes are influenced by the structure of the ligand framework and its coordination with the metal center. In general, the pincer tridentate (SNS) half-sandwich complexes were more potent than chelating bidentate (SS) half-sandwich complexes. The two osmium complexes, **6c_i_**, and **6c_iv_**, were more cytotoxic than the ruthenium complexes and were twice as effective and selective toward the tumor cells than the reference drug, 5-fluorouracil. These findings suggest that slower ligand exchange kinetics are essential for high cytotoxic activity. Isostructural ruthenium and osmium complexes have no similar biological activities; hence, their anti-proliferative activity appears to depend on the spatial arrangement of the ligand′s coordination and the nature of the metal center.

## Figures and Tables

**Table 1:** Crystallographic data and structure refinement parameters for compounds **3a_i-iii_**, **3b_ii_**, **5c_iv,_** and **6c_i_**.

	Compound						
Parameter	3a_i_	3a_ii_	3a_iii_	3b_ii_	4a_iii_	5c_iv_	6c_i_
Formula	C_22_H_31_F_6_N_5_PRuS_2_	C_22_H_32_ClF_6_N_4_PRuS_2_	C_24_H_36_ClF_6_N_4_PRuS_2_	C_21_H_30_ClF_6_N_4_PRuS_2_	C_24_H_36_ClF_6_N_4_OsPS_2_	C_27_H_35_F_12_N_5_P_2_RuS_2_	C_23_H_27_F_12_N_5_OsP_2_S_2_
Formula weight	675.68	698.12	726.18	684.10	815.31	884.73	917.75
Crystal system	triclinic	Monoclinic	monoclinic	Monoclinic	monoclinic	monoclinic	Triclinic
Space group	P-1	P21/n	P21/c	P21/n	P2_1_/n	C2/c	P-1
a/Å	9.2668(3)	13.9150(2)	20.0391(3)	11.7443(2)	14.3155(13)	27.0151(7)	11.4823(7)
b/Å	11.4774(3)	14.4134(2)	14.6704(2)	17.4566(4)	14.9434(15)	11.2276(3)	11.9080(6)
c/Å	16.7090(5)	14.0586(2)	20.3946(3)	14.1523(3)	14.5774(14)	26.3787(7)	13.5933(8)
α/°	70.0180(10)	90	90	90	90	90	73.725(2)
β/°	88.453(2)	92.5410(10)	91.8760(10)	111.8930(10)	90.364(5)	113.2150(10)	65.784(2)
γ/°	69.1310(10)	90	90	90	90	90	64.656(2)
Volume/Å^3^	1551.49(8)	2816.85(7)	5992.42(15)	2692.19(10)	3118.4(5)	7353.2(3)	1519.53(15)
Z	2	4	8	4	4	8	2
ρ_calc_ g/cm^3^	1.446	1.646	1.610	1.688	1.737	1.598	2.006
Temperature/K	149.9	149.99	149.99	149.98	296.15	296	150.0
μ/mm^−1^	0.747	0.916	0.865	0.957	4.417	0.715	4.538
λ/Å	0.71073	0.71073	0.71073	0.71073	0.71073	0.71073	0.71073
F(000)	686.0	1416.0	2960.0	1384.0	1608.0	3568.0	892.0
Cryst size, mm^3^	0.31 × 0.25 × 0.14	0.35 × 0.27 × 0.14	0.28 × 0.24 × 0.13	0.25 × 0.17 × 0.13	0.18 × 0.12 × 0.08	0.26 × 0.21 × 0.14	0.18 × 0.16 × 0.11
*θ*min, *θ*max,^◦^	3.972 to 55.754	6.354 to 56.844	2.034 to 56.666	5.604 to 56.682	4.82 to 56.546	3.982 to 56.618	4.368 to 57
No. of reflns. Collected	24,335	28,323	97,276	44,694	46,353	40,270	30,272
No of indep. reflns.	7328	6969	14,899	6643	7666	9032	7443
Completeness to theta	27.877(99.1%)	28.422(98.4%)	28.333(99.6%)	28.341(98.9%)	28.273(99.1%)	28.309(98.7%)	28.500(96.6%)
Absorbed correction	MULTI-SCAN	MULTI-SCAN	MULTI-SCAN	MULTI-SCAN	MULTI-SCAN	MULTI-SCAN	MULTI-SCAN
Goodness-of-fit on F^2^	1.025	1.060	1.034	1.097	1.041	1.048	1.038
Final R indices	0.0255, 0.0624	0.0209, 0.0488	0.0331, 0.0758	0.0388, 0.1145	0.0342, 0.0770	0.0587, 0.1727	0.0180, 0.0430
R indices (all data)	0.0284, 0.0645	0.0245, 0.0508	0.0445, 0.0834	0.0440, 0.1192	0.0565, 0.0871	0.0700, 0.1839	0.0199, 0.0439
Largest diff. peak/hole/eÅ^−3^	0.69/−0.38	0.62/−0.32	1.08/−0.72	1.31/−1.22	1.04/−0.67	1.48/−0.78	0.90/−0.47

**Table 2:** Selected bond distances (Å) and angles (°) for complexes **3a_i_**, **3a_ii_**, **3a_iii_**, **3b_ii_**, **4a_iii_**, **5c_iv_,** and **6c**_i_.

Compounds							
Bond Distances (Å)							
Bond/Angle	3a_i_ (Ru)	3a_ii_ (Ru)	3a_iii_ (Ru)	3b_ii_ (Ru)	4a_iii_ (Os)	5c_iv_ (Ru)	6c_i_ (Os)
M-S	Ru(1)-S(1) 2.4182(5)	Ru(1)-S(1) 2.4405(4)	Ru(1)-S(1) 2.4426(6)	Ru(1)-S(1) 2.4479(7)	Os(1)-S(1) 2.4478(13)	Ru(1)-S(1) 2.3857(10)	Os(1)-S(1) 2.4113(6)
	Ru(1)-S(2) 2.4656(5)	Ru(1)-S(2) 2.4726(4)	Ru(1)-S(2) 2.4698(6)	Ru(1)-S(2) 2.4320(6)	Os(1)-S(2) 2.4488(13)	Ru(1)-S(2) 2.4071(10)	Os(1)-S(2) 2.3905(5)
M-Cl		Ru(1)-Cl(1) 2.4072(4)	Ru(1)-Cl(1) 2.4030(6)	Ru(1)-Cl(1) 2.4018(7)	Os(1)-Cl(1) 2.4027(12)		
M-N	Ru(1)-N(5) 2.0710(16)					Ru(1)-N(3) 2.168(3)	Os(1)-N(3) 2.1733(18)
C-S	S(1)-C(2) 1.7222(19)	S(1)-C(5) 1.7196(15)	S(1)-C(4) 1.720(2)	S(1)-C(5) 1.718(3)	S(1)-C(16) 1.715(6)	S(1)-C(16) 1.706(4)	S(1)-C(4) 1.706(2)
	S(2)-C(7) 1.7170(19)	S(2)-C(10) 1.7186(17)	S(2)-C(9) 1.719(2)	S(2)-C(7) 1.716(3)	S(2)-C(22) 1.715(5)	S(2)-C(22) 1.701(4)	S(2)-C(10) 1.699(2)
Bond angles (°)							
S-M-S	S(1)-Ru(1)-S(2) 88.255(17)	S(1)-Ru(1)-S(2) 92.835(14)	S(1)-Ru(1)-S(2) 91.02(2)	S(1)-Ru(1)-S(2) 87.33(2)	S(1)-Os(1)-S(2) 90.94(6)	S(1)-Ru(1)-S(2) 84.70(4)	S(2)-Os(1)-S(1) 86.07(2)
S-M-Cl		C1(1)-Ru(1)-S(1) 89.034(13)	Cl(1)-Ru(1)-S(1) 89.98(2)	Cl(1)-Ru(1)-S(1) 91.42(2)	Cl(1)-Os(1)-S(1) 89.61(5)		
		Cl(1)-Ru(1)-S(2) 90.985(13)	Cl(1)-Ru(1)-S(2) 91.16(2)	Cl(1)-Ru(1)-S(2) 90.43(2)	Cl(1)-Os(1)-S(2) 90.18(4)		
S-M-N	N(5)-Ru(1)-S(1) 90.65(4)					N(3)-Ru(1)-S(1) 84.62(9)	N(3)-Os(1)-S(1) 84.41(5)
	N(5)-Ru(1)-S(2) 90.74(4)					N(3)-Ru(1)-S(2) 84.23(9)	N(3)-Os(1)-S(2) 84.67(5)

**Table 3:** Ruthenium and osmium complexes MTT cytotoxicity test results.

S/No.	Compound	IC_50_/μM ^a^	
		HEK293	HeLa
1	3a_i_	180 ± 23	219 ± 55
2	3a_ii_	155 ± 13	132 ± 41
3	3a_iii_	159 ± 33	344 ± 26
4	3b_ii_	29 ± 7	191 ± 25
5	4a_iii_	173 ± 23	170 ± 16
6	4b_i_	47 ± 18	469 ± 21
7	4b_ii_	211 ± 11	315 ± 62
8	5b_i_	155 ± 5	86 ± 9
9	5c_iv_	169 ± 7	147 ± 21
10	6b_i_	18 ± 7	151 ± 12
11	6c_i_	121 ± 8	36 ± 10
12	6c_iv_	121 ± 4	40 ± 4
13	5-Fluorouracil	180 ± 20	87 ± 15

^a^ IC_50_ values correspond to the concentration of the respective compound needed to cause 50% net cell mortality.

## Data Availability

The data presented in this study are available in the article and Supplementary Materials.

## Supplementary Materials

The following supporting information can be downloaded at: https://www.mdpi.com/article/10.3390/molecules29050944/s1.
