# Supplementary material for: Chemotherapeutic Activities of New η6-p-Cymene Ruthenium(II) and Osmium(II) Complexes with Chelating SS and Tridentate SNS Ligands
Source: Molecules. 2024 Feb 21;29(5):944. doi: 10.3390/molecules29050944 (PMC10935334; doi:10.3390/molecules29050944)
Supplement: Supplementary file 1 [file molecules-29-00944-s001.zip › molecules-2825010-supplementary.pdf]

Supplementary Information

FT-IR for **3ai**

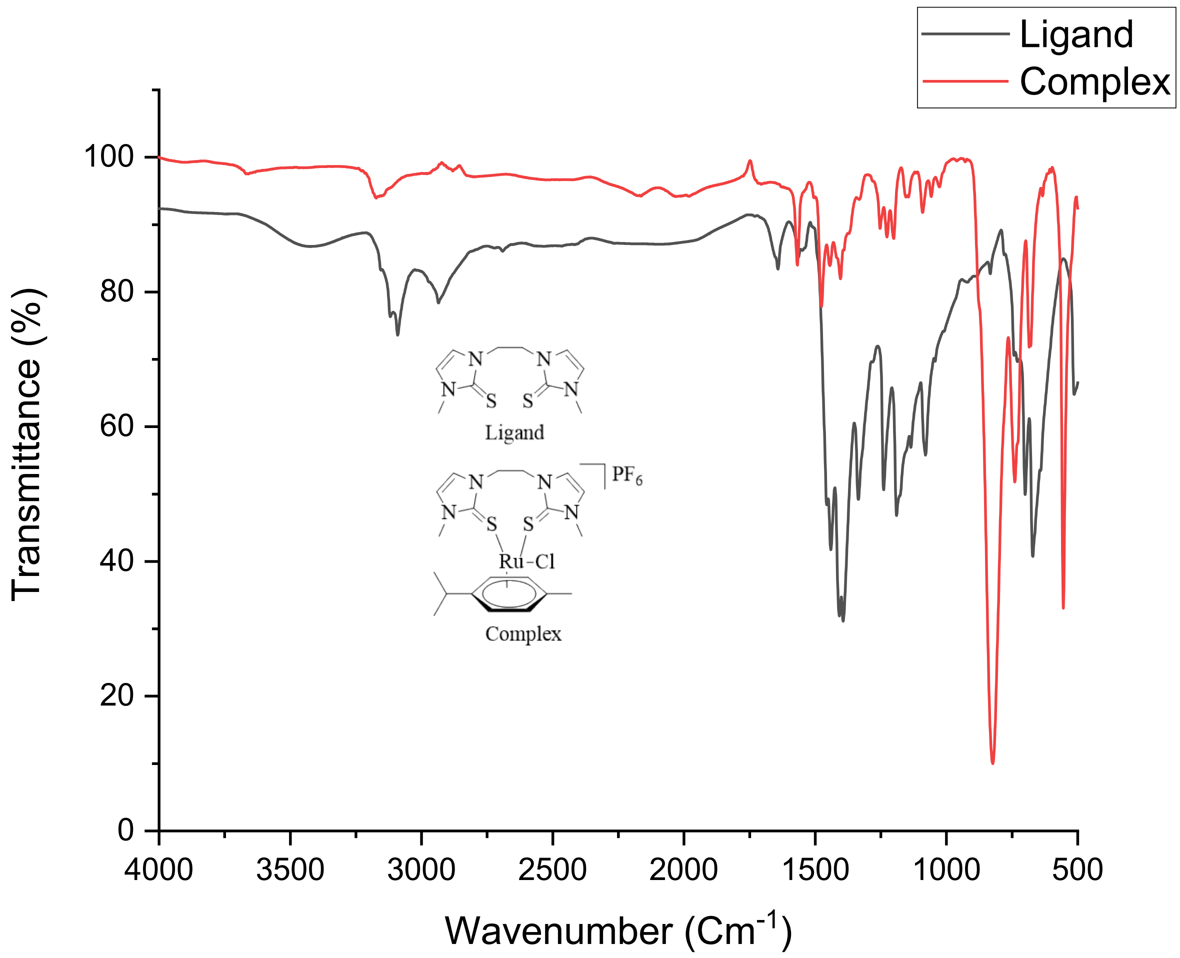

FT-IR of **3aii**

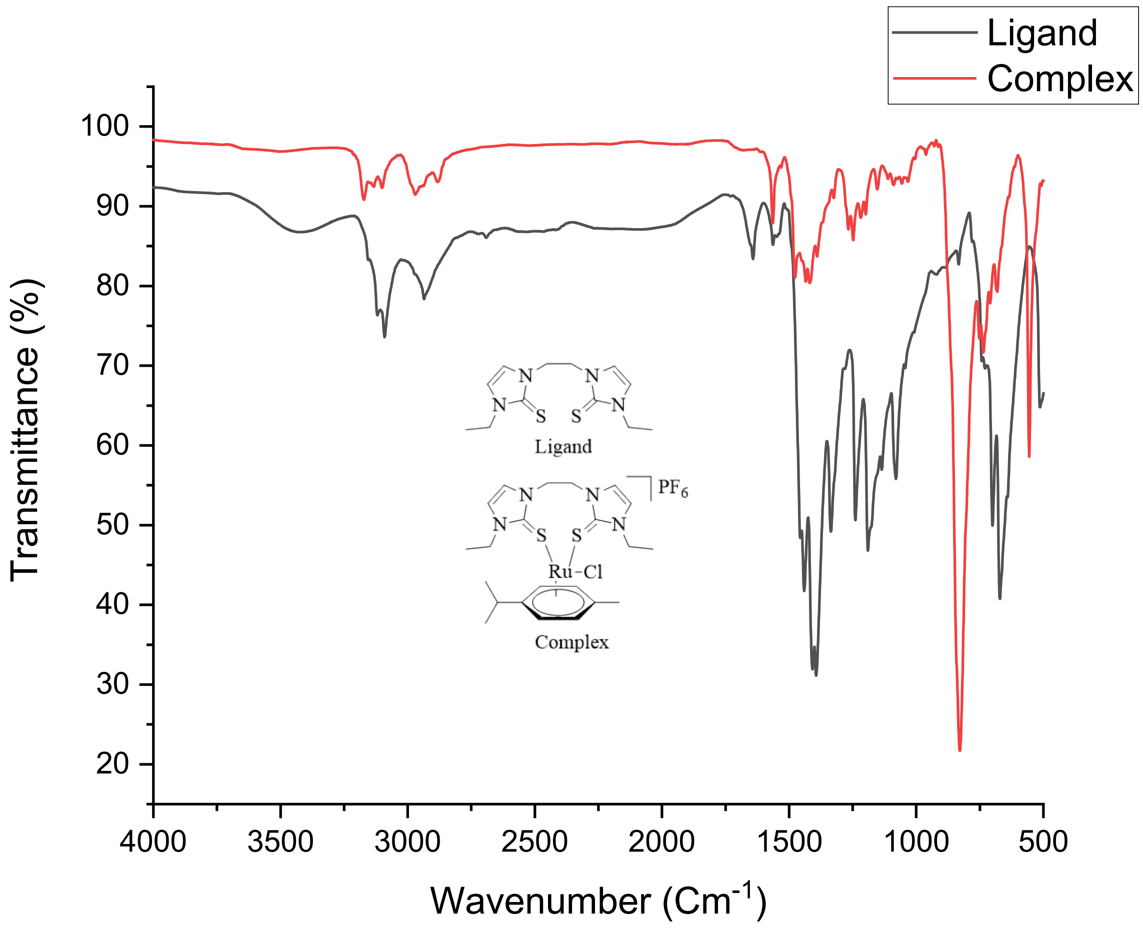

Ligand
Complex
100
90
80
70
60
50
40
30
20
Transmittance (%)
4000
3500
3000
2500
2000
1500
1000
500
Wavenumber (Cm-1)
N
N
N
N
S
S
Ligand
PF6
Ru-Cl
Complex

FT-IR of **3aiii**

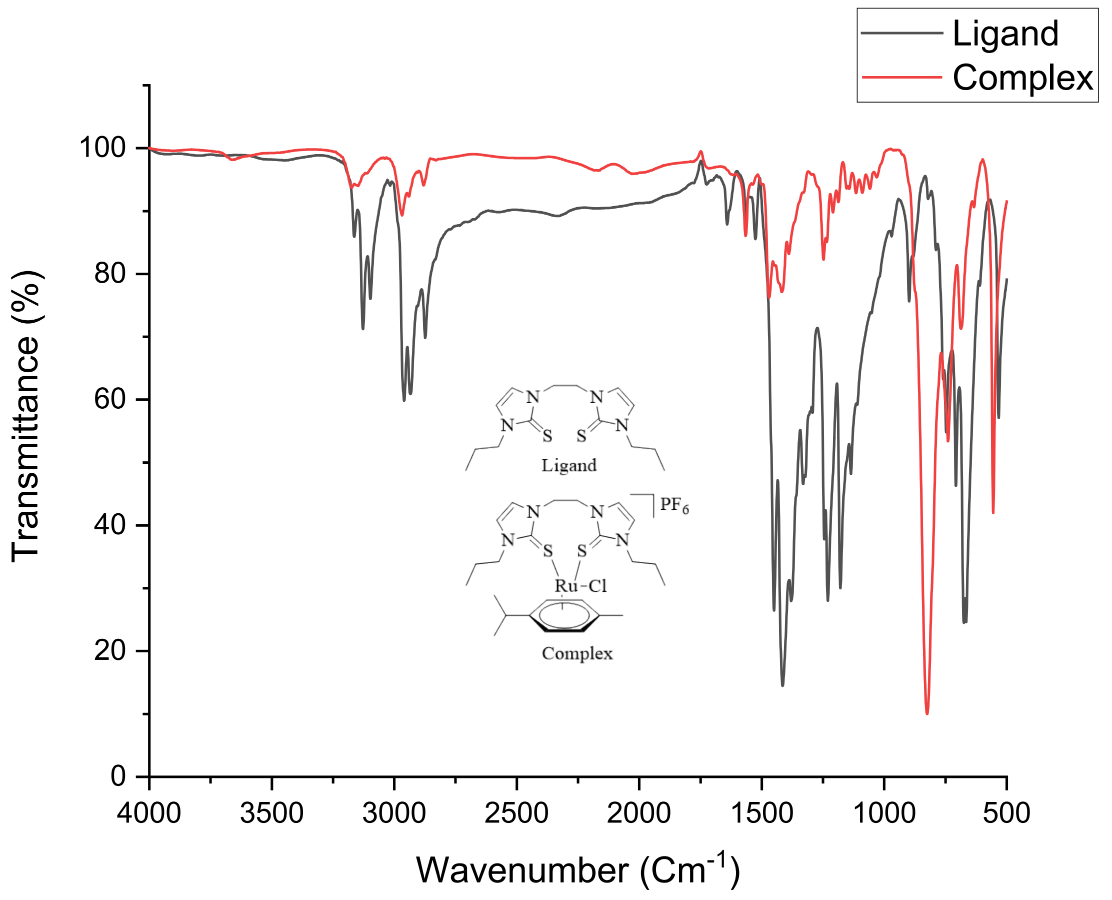

FT-IR of **3bii**

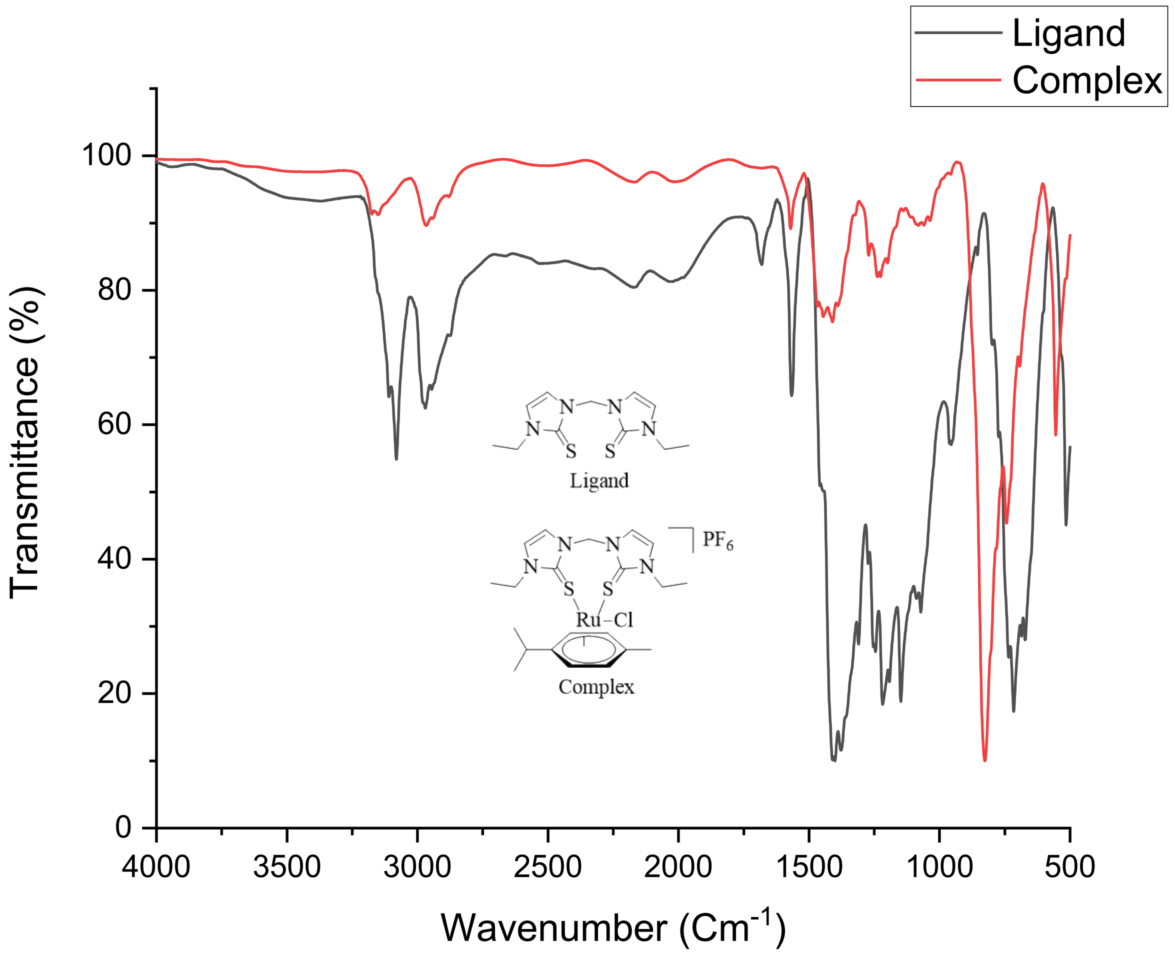

Ligand
Complex
Transmittance (%)
Wavenumber (Cm-1)
0
20
40
60
80
100
4000
3500
3000
2500
2000
1500
1000
500
N
N
N
N
S
S
Ligand
PF6
Ru
Cl
Complex

FT-IR of **4aiii**

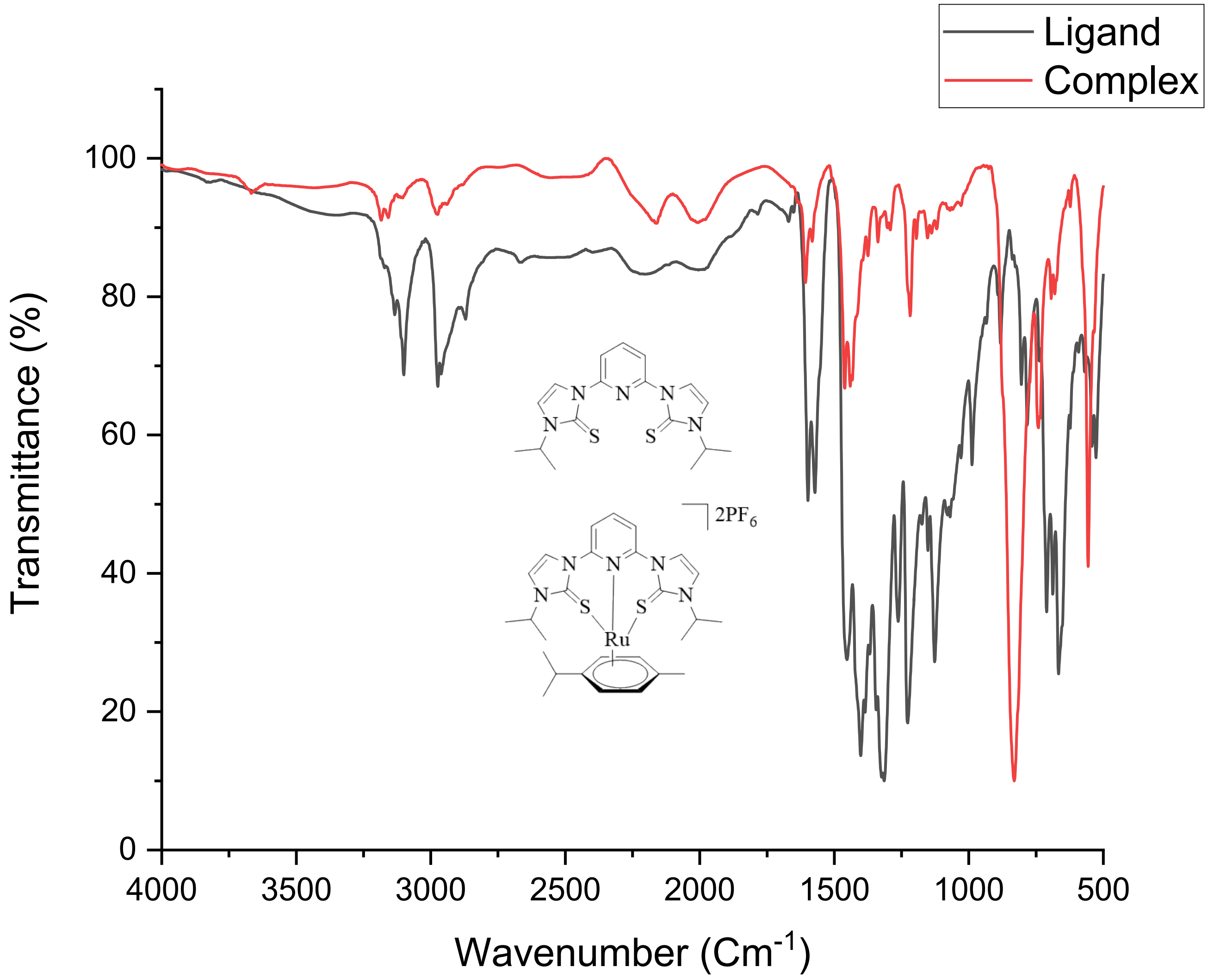

FT-IR of **4bi**

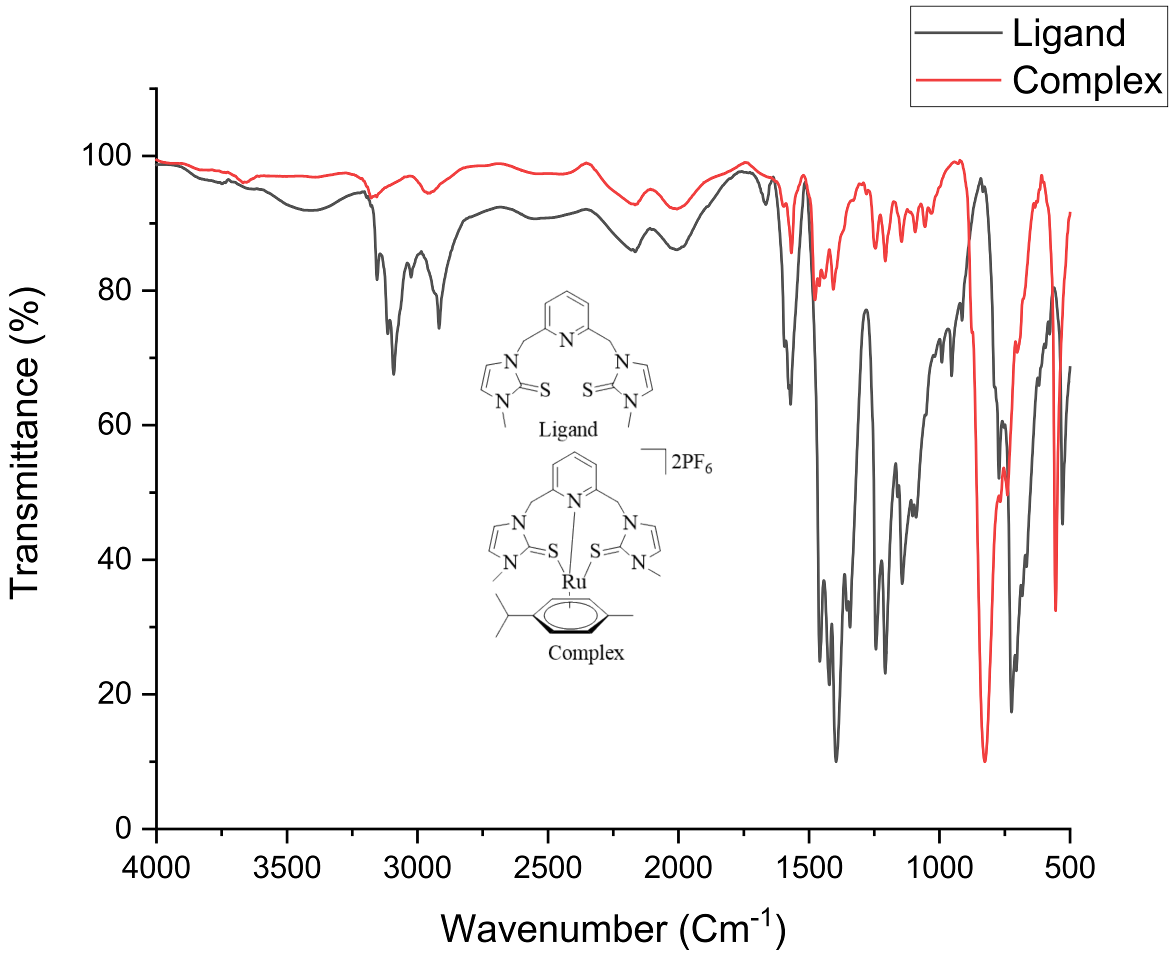

Ligand
Complex
Transmittance (%)
Wavenumber (Cm-1)
100
80
60
40
20
0
4000
3500
3000
2500
2000
1500
1000
500
N
S
Ru
2PF6

FT-IR of **4bii**

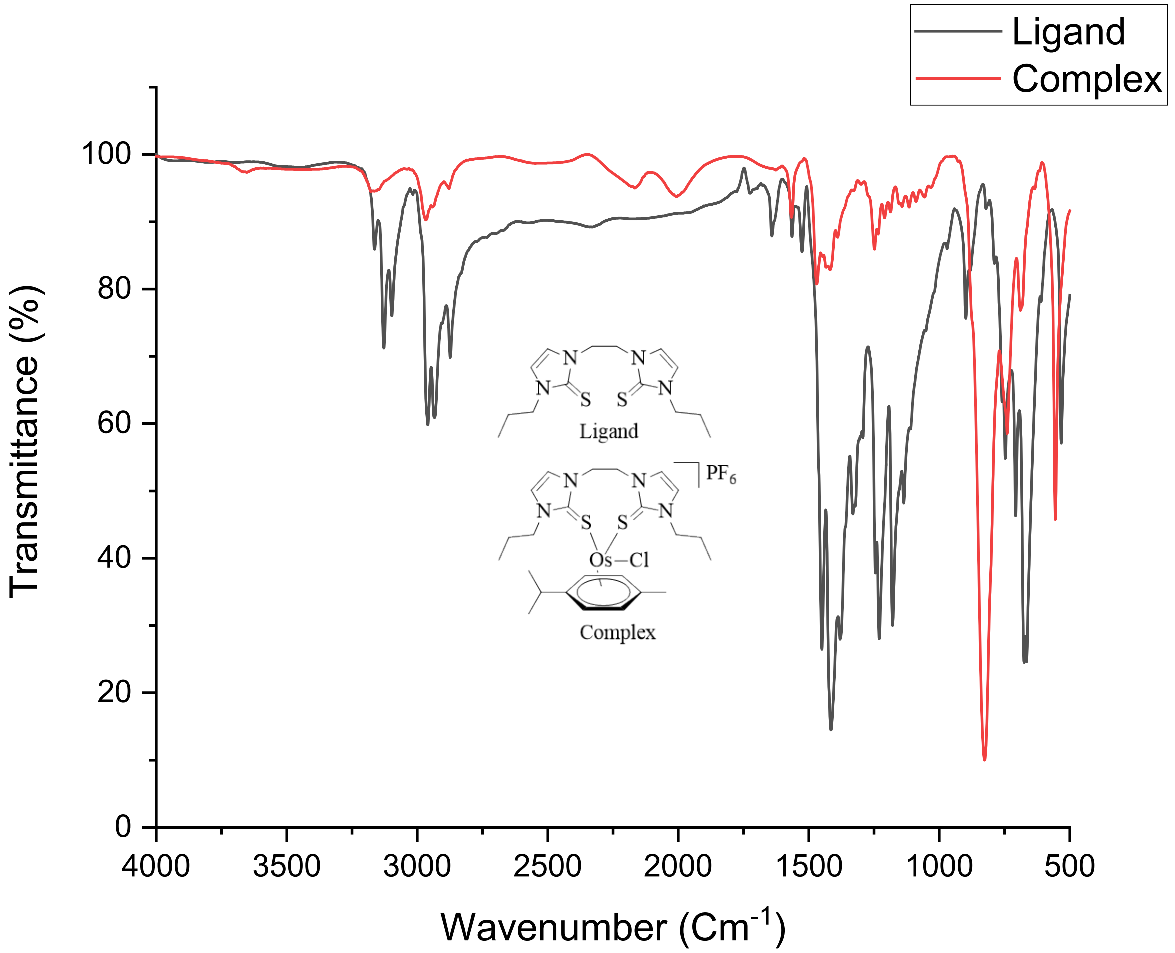
Ligand
Complex
Transmittance (%)
Wavenumber (Cm-1)
0
20
40
60
80
100
4000
3500
3000
2500
2000
1500
1000
500
N
N
N
N
S
S
Ligand
PF6
Os
Cl
Complex

FT-IR of **4bi**

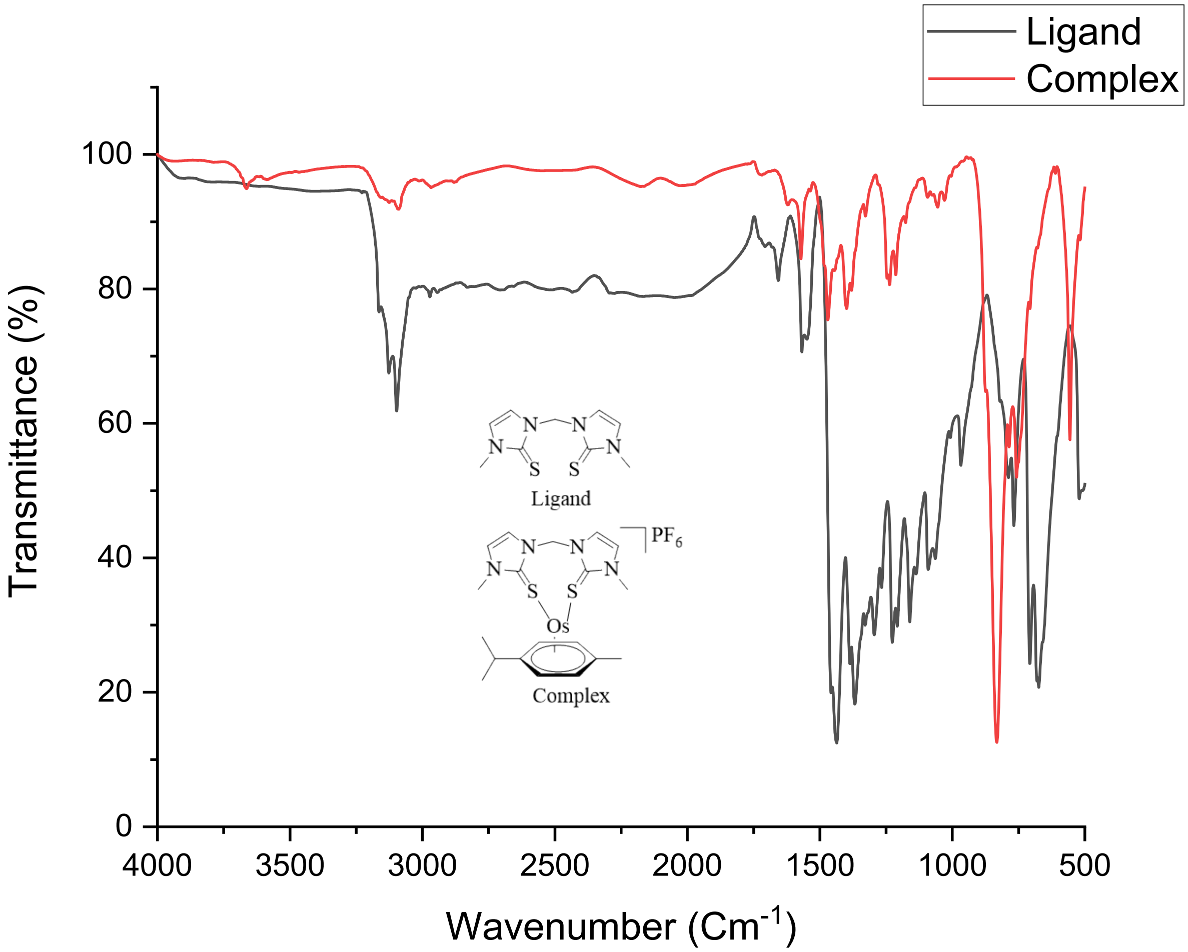

Ligand
Complex
Transmittance (%)
Wavenumber (Cm-1)
0
20
40
60
80
100
4000
3500
3000
2500
2000
1500
1000
500
N
N
N
N
S
S
Ligand
PF6
Os
Complex

FT-IR **5civ**

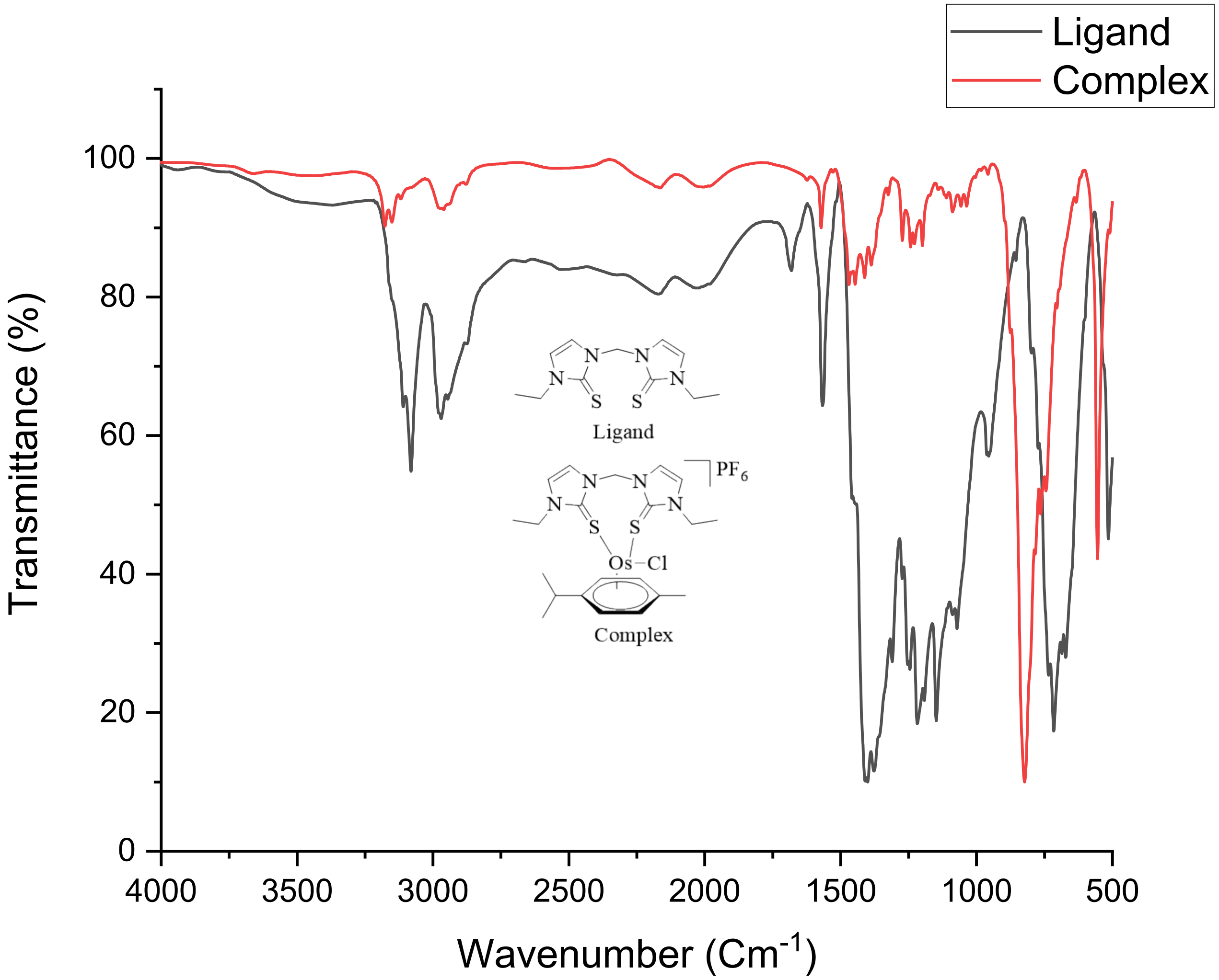
Ligand
Complex
Transmittance (%)
Wavenumber (Cm-1)
0
20
40
60
80
100
4000
3500
3000
2500
2000
1500
1000
500
PF6
Os
Cl

FT-IR of **6bi**

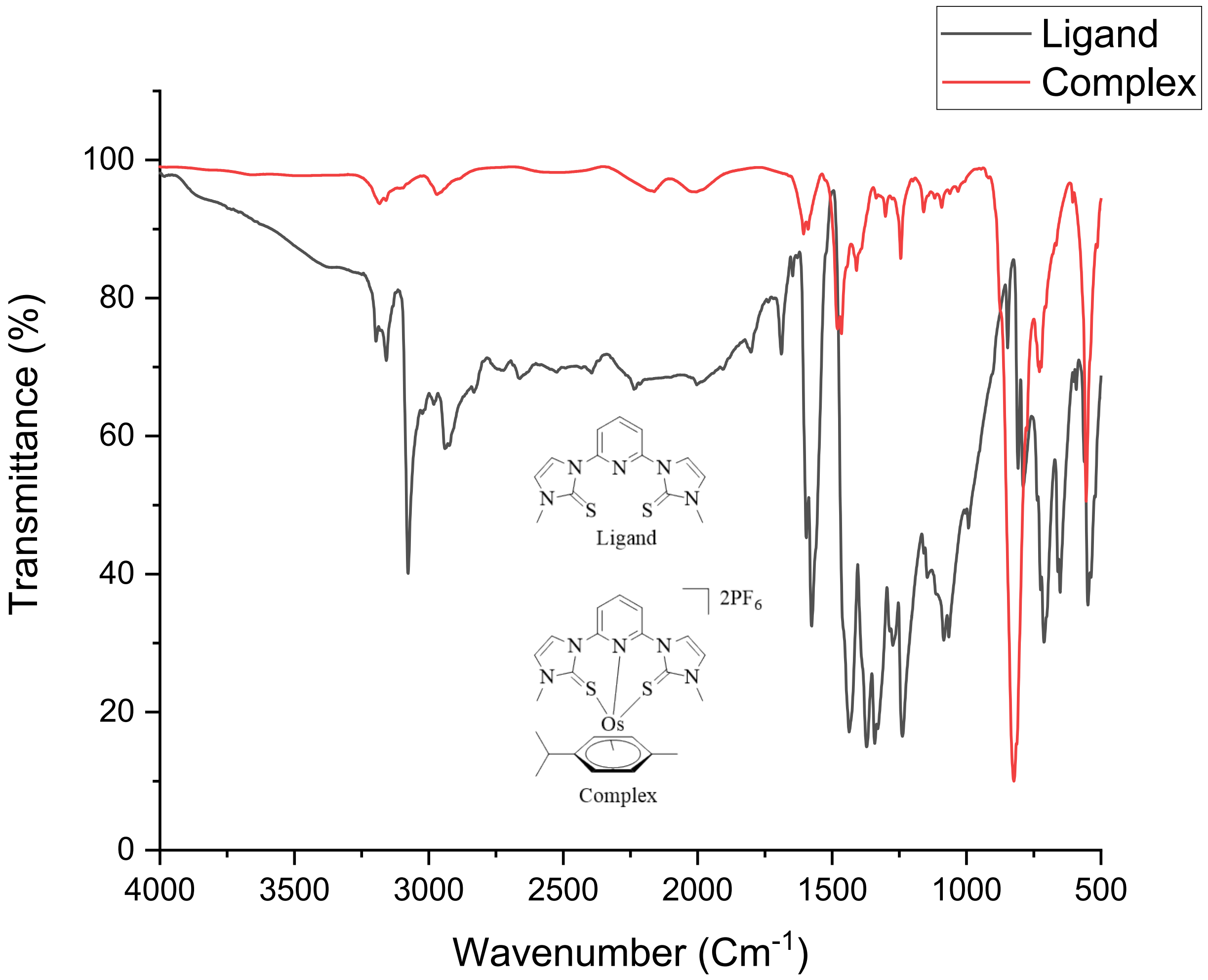

Ligand
Complex
Transmittance (%)
Wavenumber (Cm-1)
0
20
40
60
80
100
4000
3500
3000
2500
2000
1500
1000
500
N
N
N
N
N
S
S
Ligand
2PF6
Os
Complex

FT-IR of **6ci**

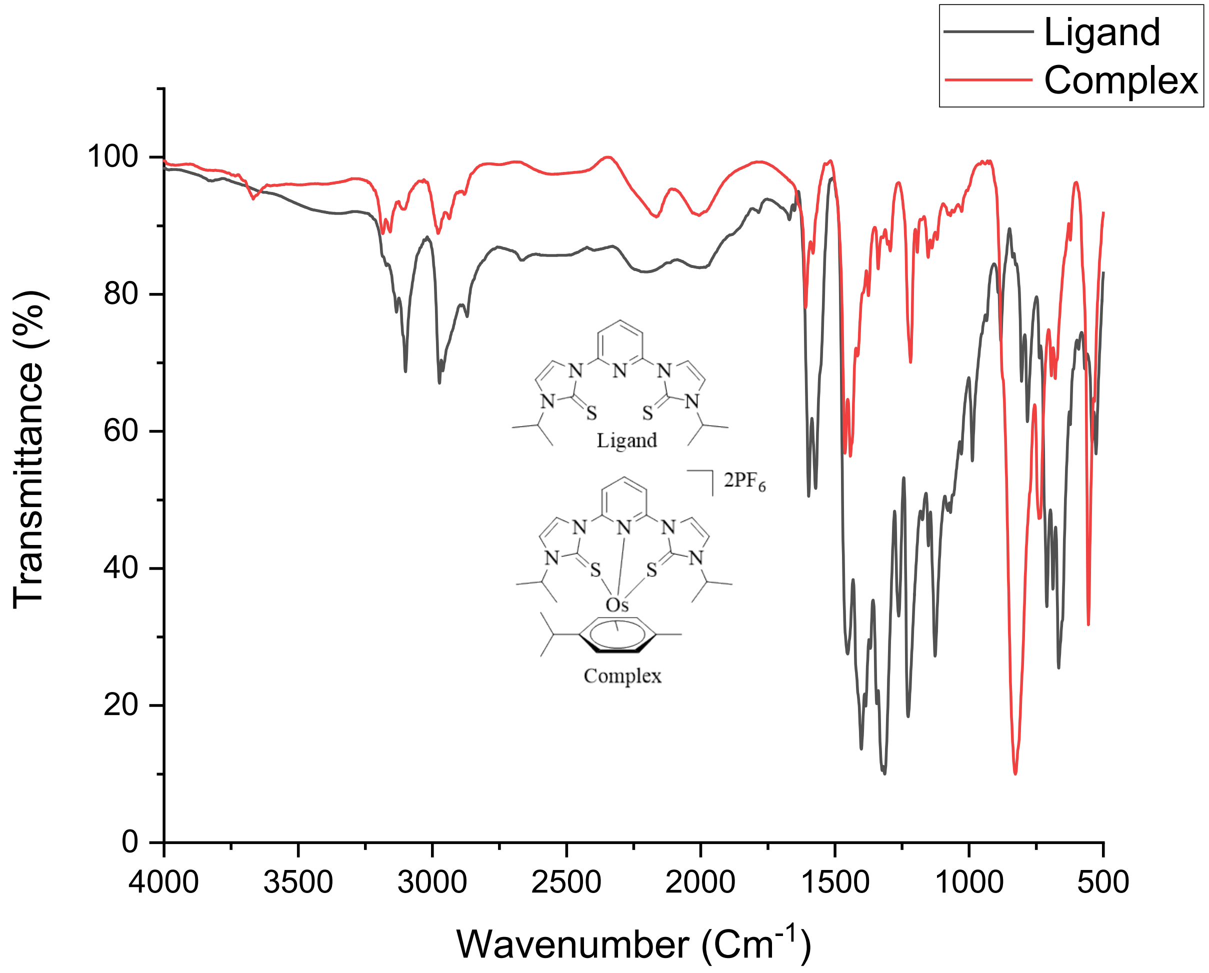
Ligand
Complex
Transmittance (%)
Wavenumber (Cm-1)
100
80
60
40
20
0
4000
3500
3000
2500
2000
1500
1000
500
Ligand
2PF6
Os
Complex

FT-IR of **6civ**

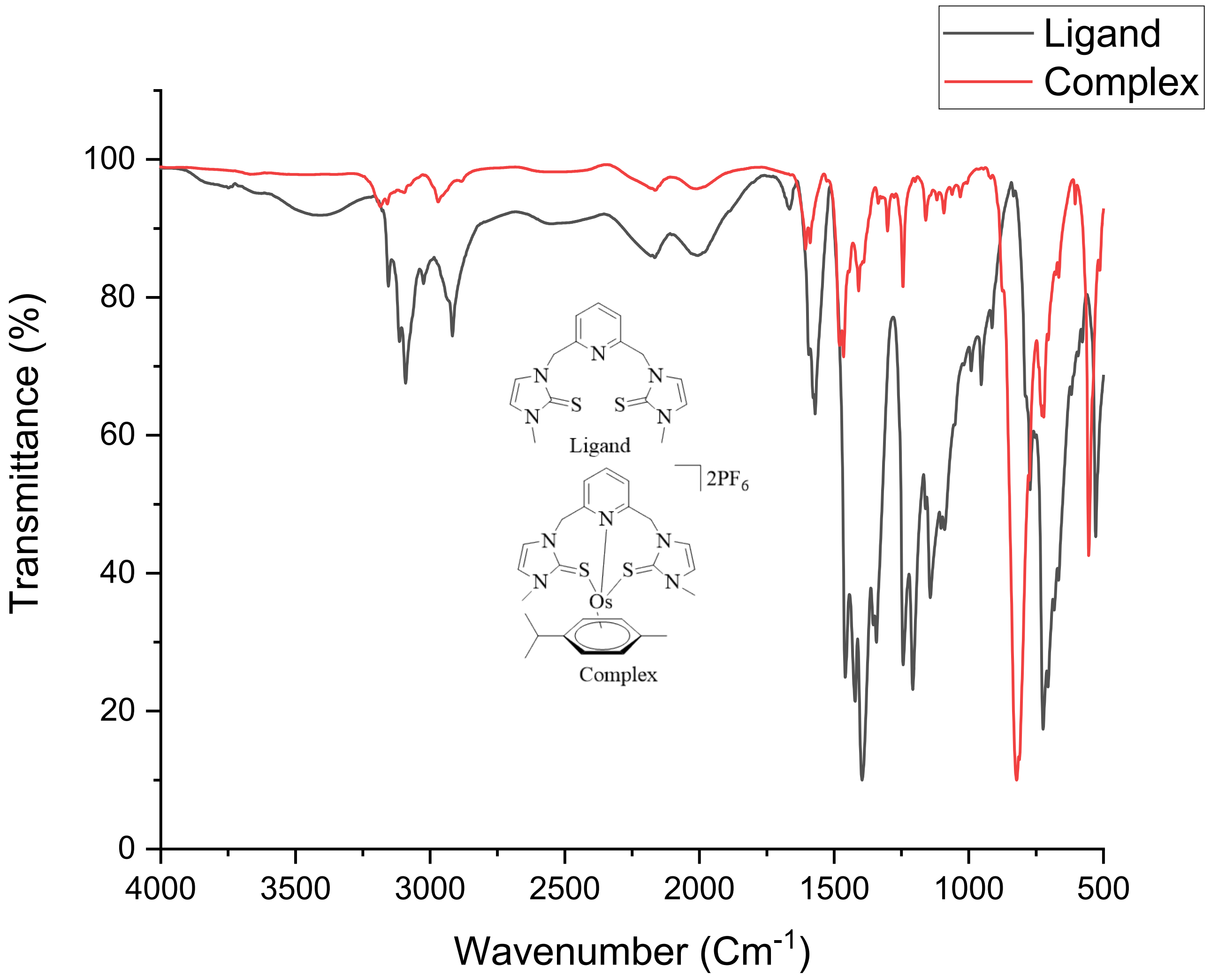

Ligand
Complex
100
80
60
40
20
0
Transmittance (%)
4000
3500
3000
2500
2000
1500
1000
500
Wavenumber (Cm-1)
N
N
S
S
N
N
N
Ligand
2PF6
N
N
S
S
N
N
N
Os
Complex

^{1}H NMR of **3ai**

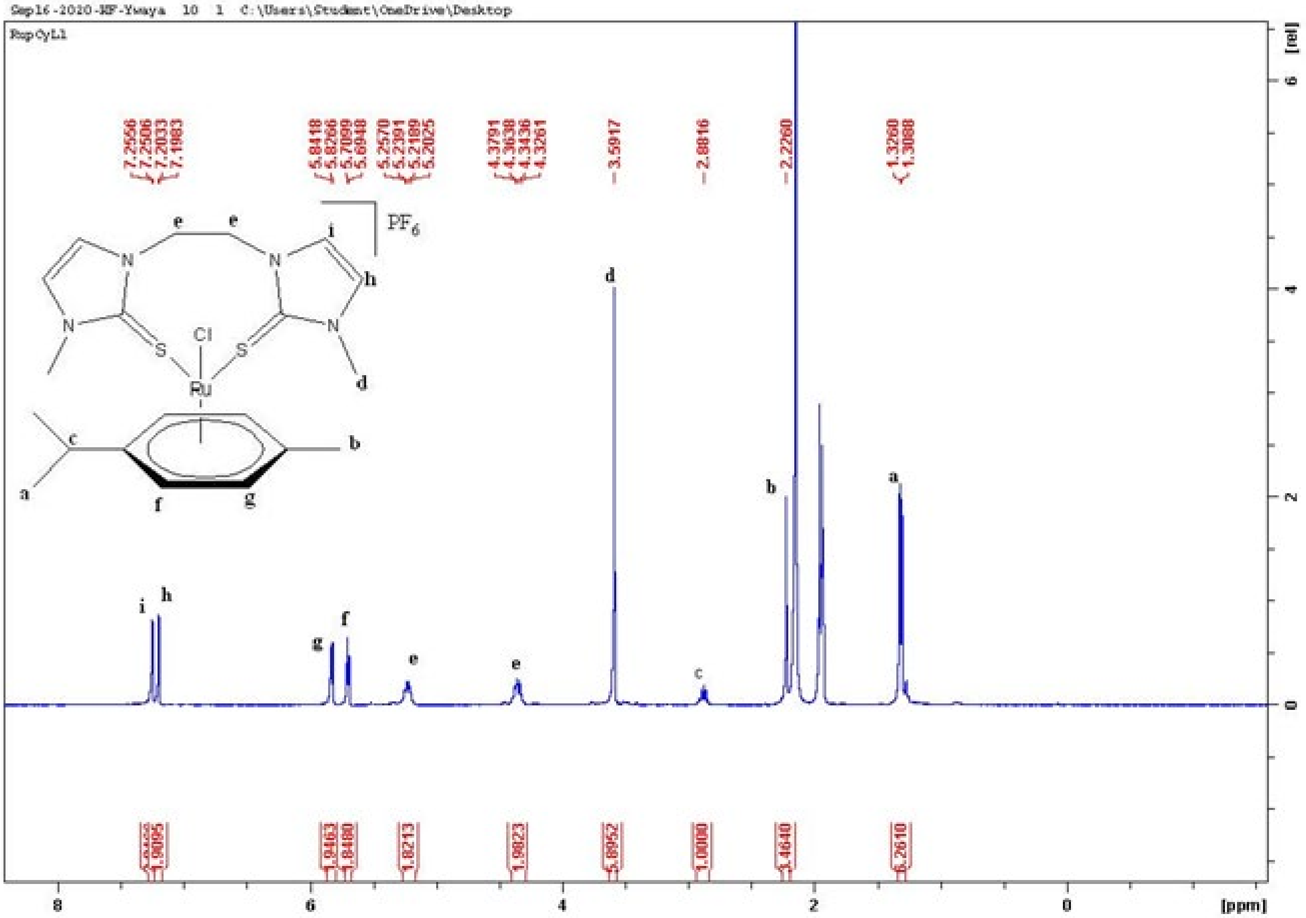
Sep16-2020-RF-Ywaya 10 1 C:\Users\Student\OneDrive\Desktop
RupCyL1
7.2556
7.2506
7.2033
7.1983
5.8418
5.8266
5.7099
5.6948
5.2570
5.2391
5.2189
5.2025
4.3791
4.3638
4.3436
4.3261
3.5917
2.8816
2.2260
1.3260
1.3088
PF_6
Cl
Ru
N
S
a
b
c
d
e
f
g
h
i
1.9095
1.9463
1.8480
1.8213
1.9823
5.8952
1.0000
3.4640
6.2610
[rel]
[ppm]

^{13}C NMR of **3ai**

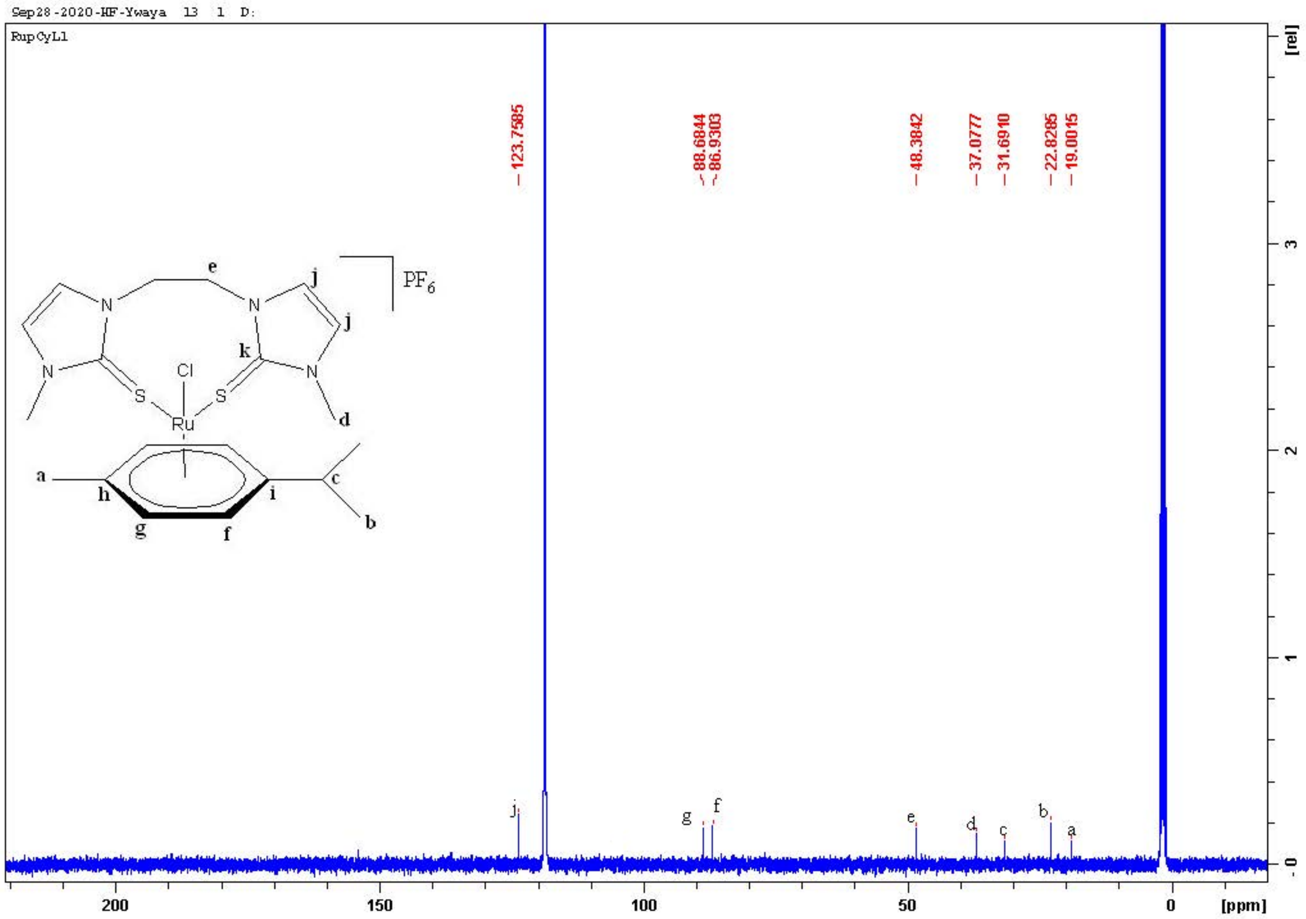

^{1}H NMR of **3aii**

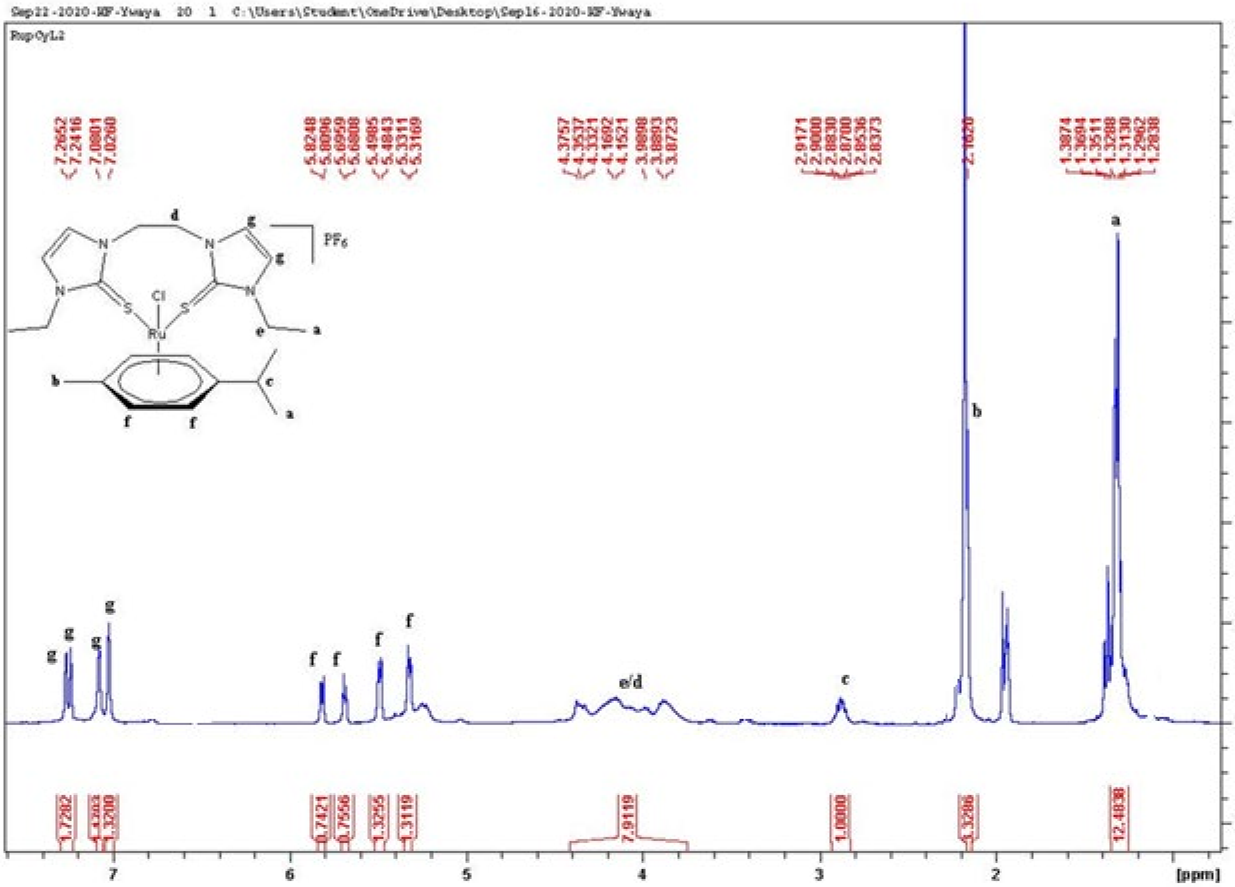

^{13}C NMR of **3aii**

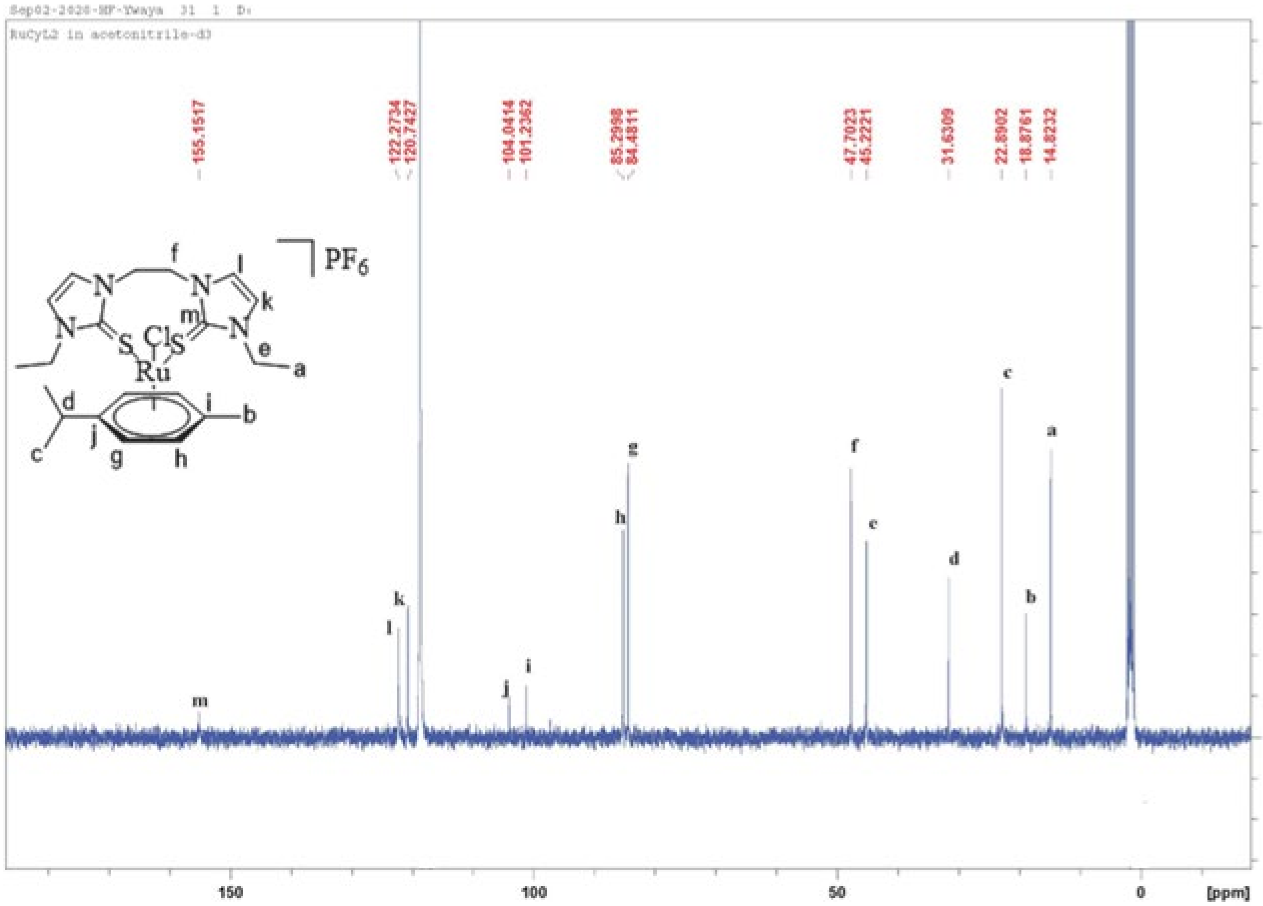

^{1}H NMR of **3aiii**

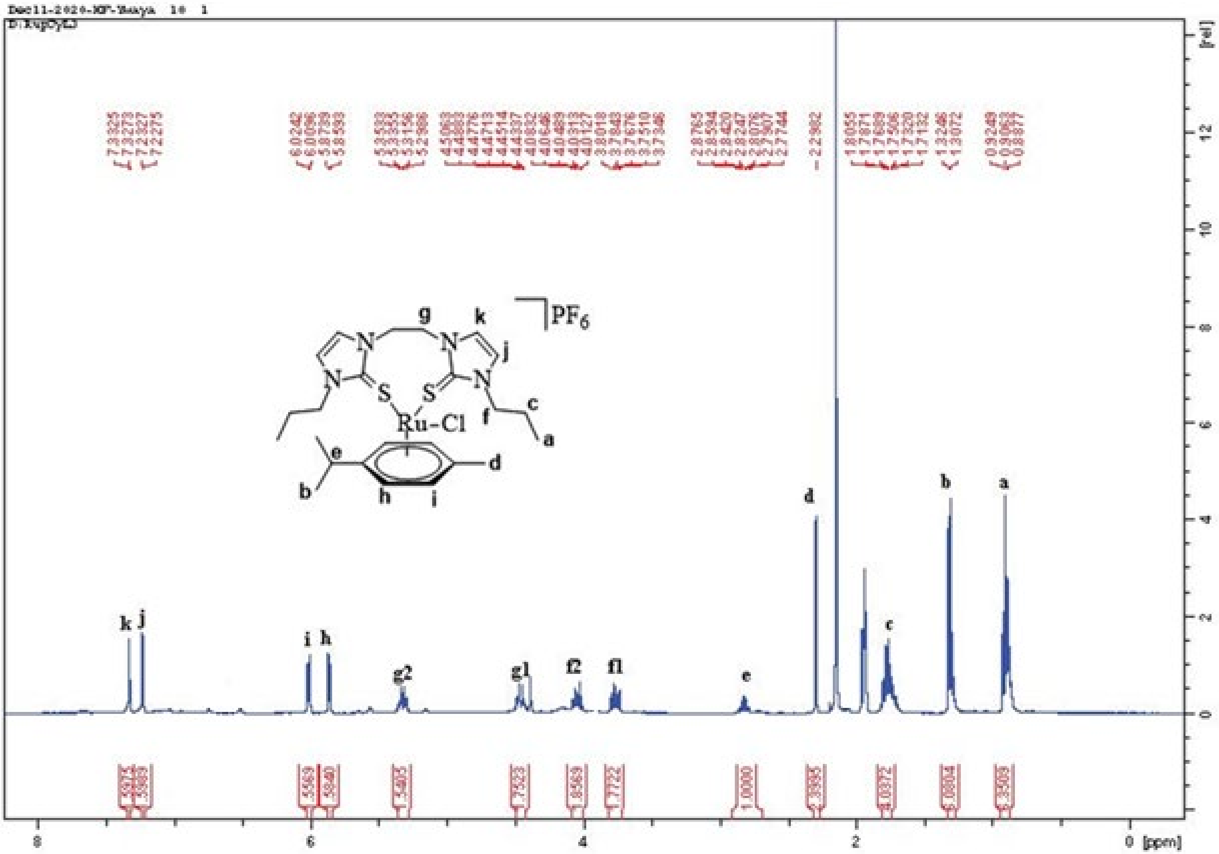

^{13}C NMR of **3aiii**

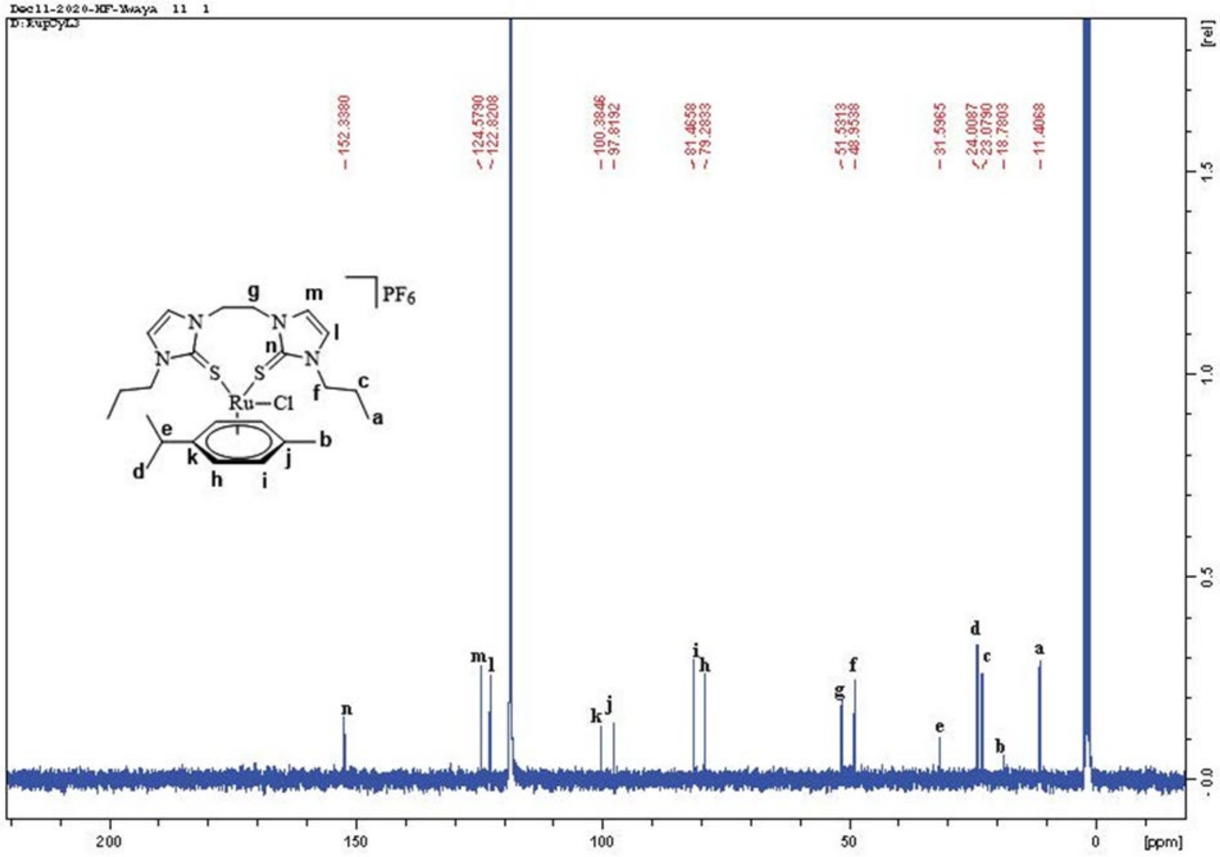

^1H NMR of **3bii**

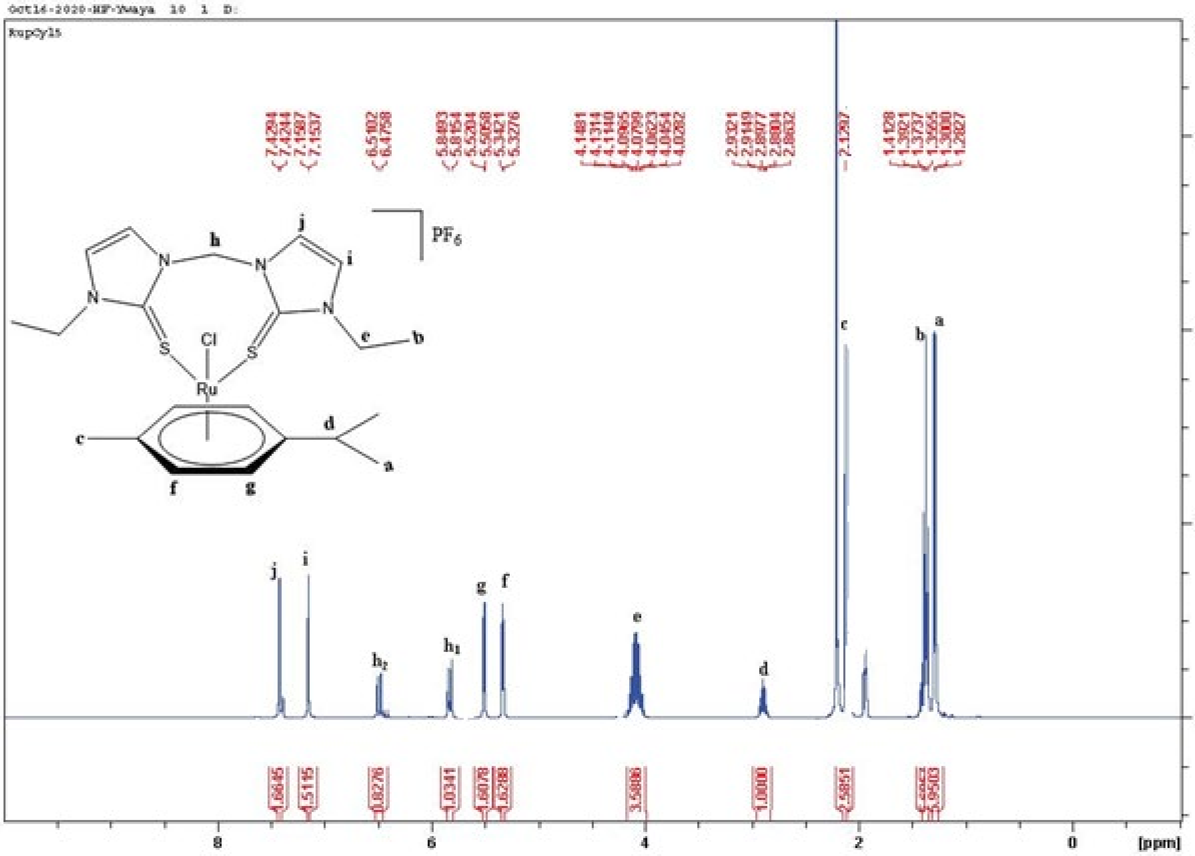

^{13}C NMR of **3bii**

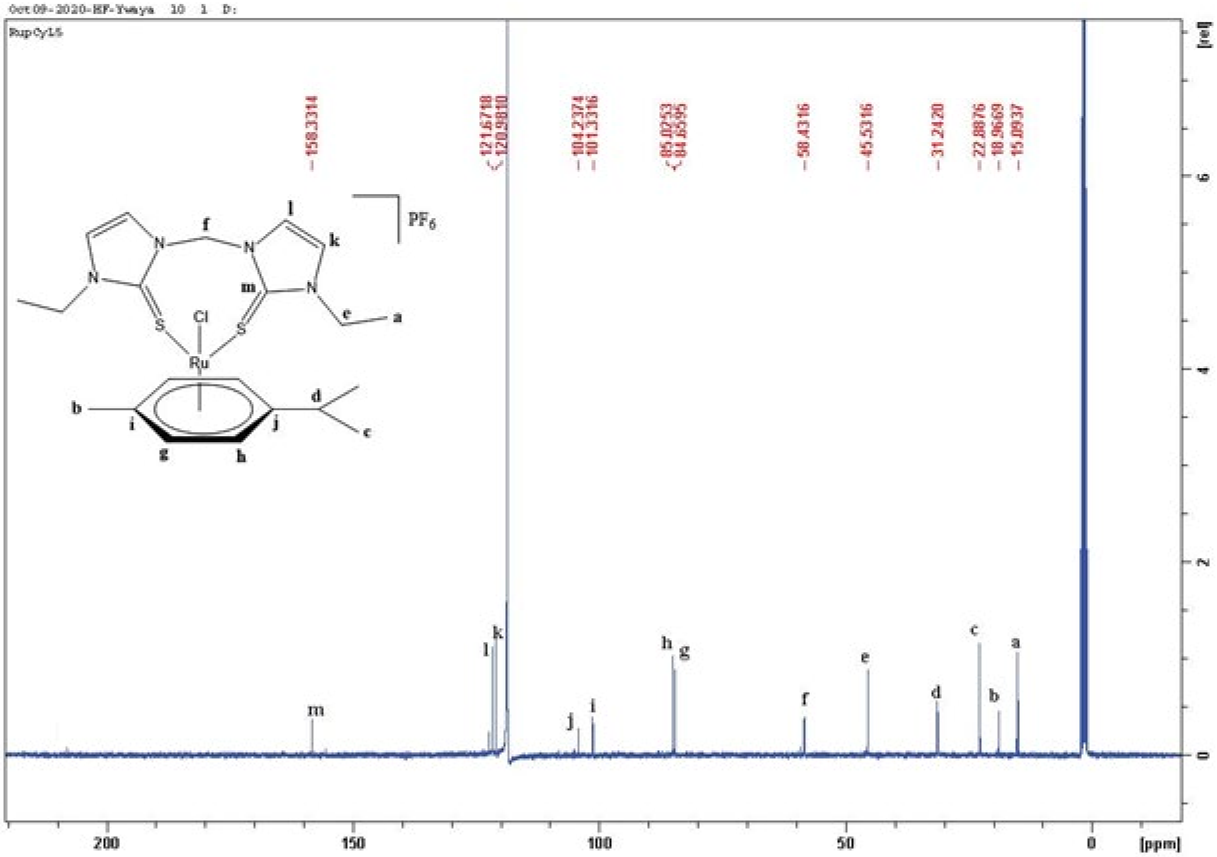

^{1}H NMR of **4aiii**

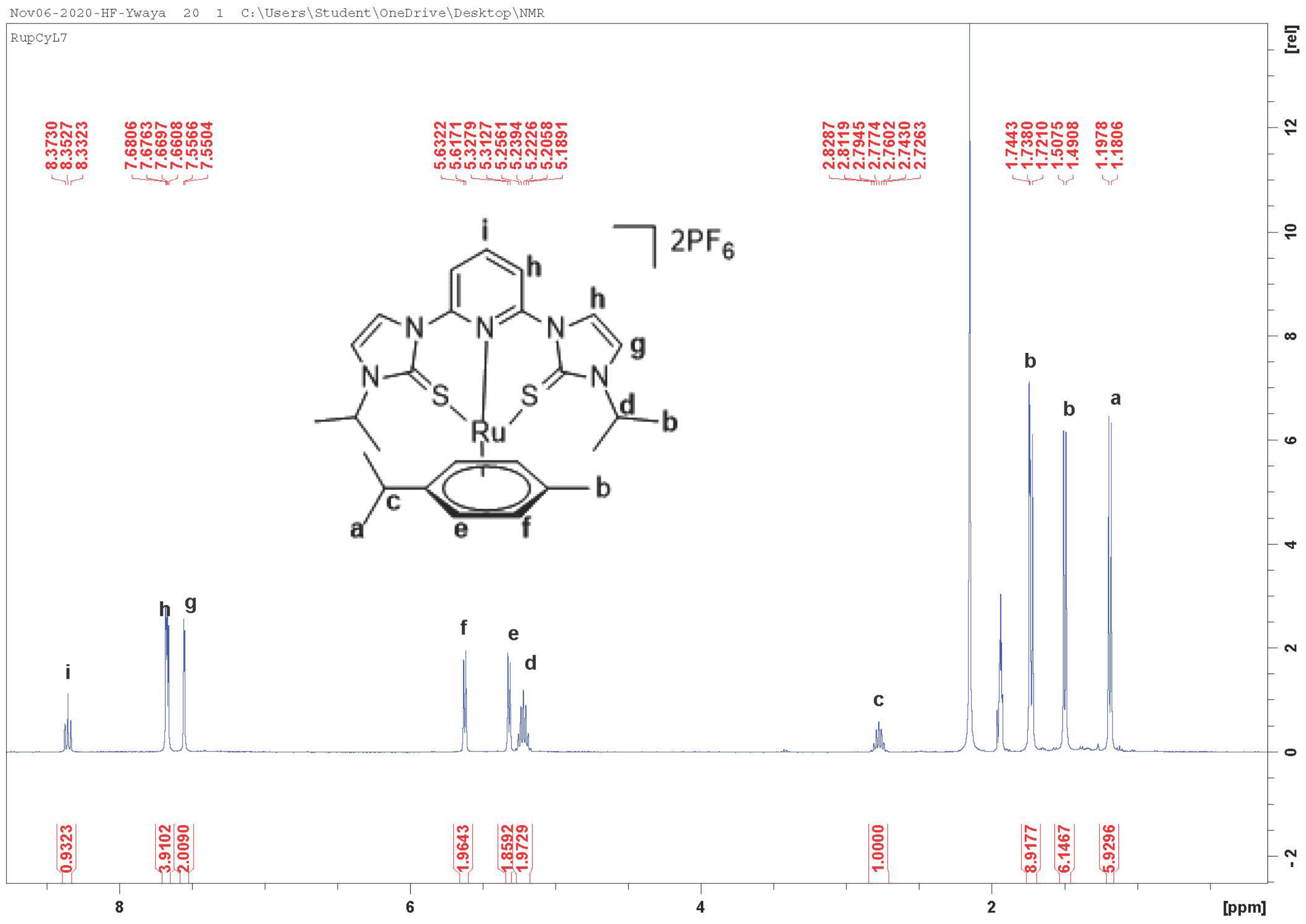

^{13}NMR for **4aiii**

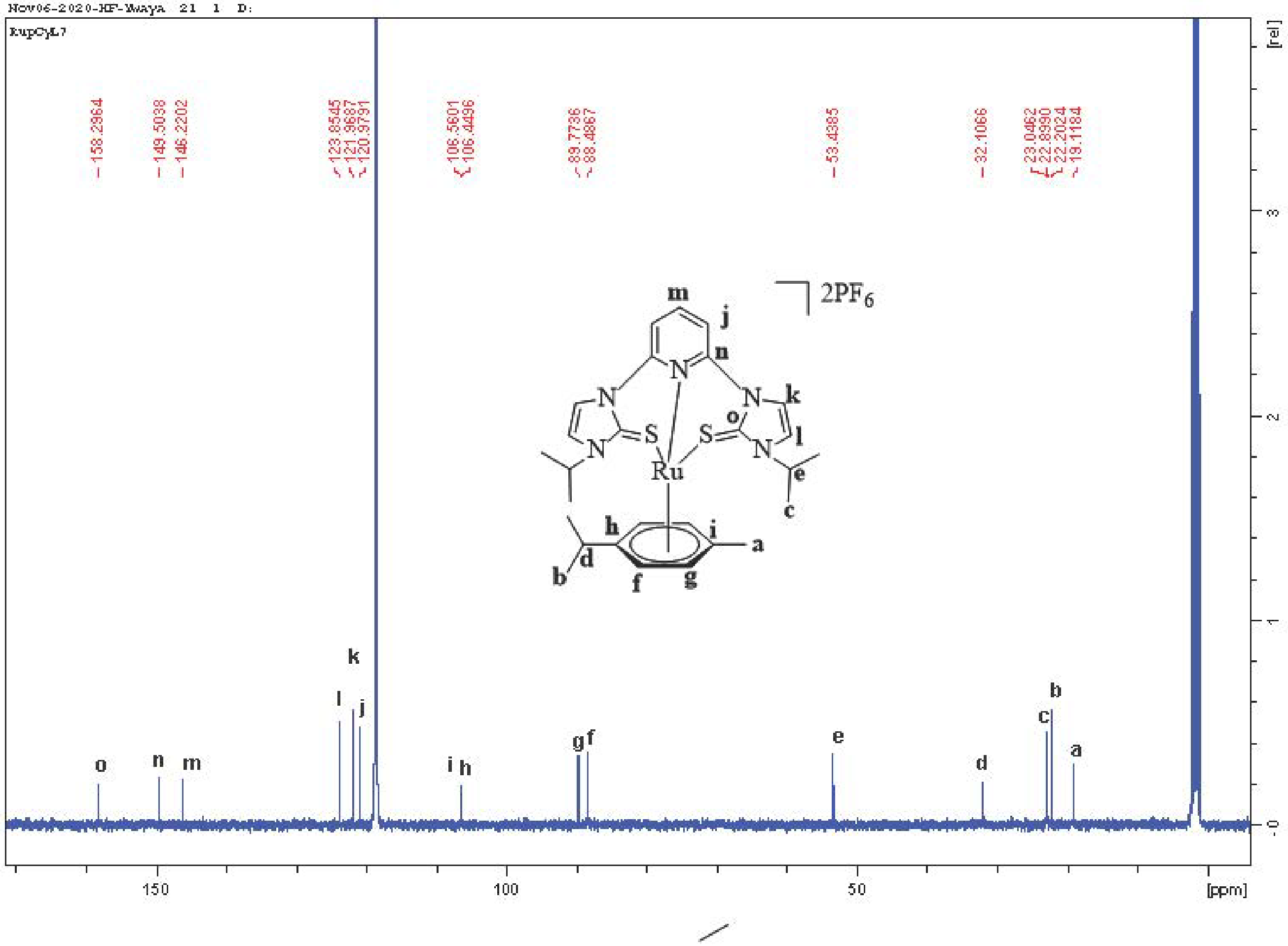

^{1}H NMR of **4bi**

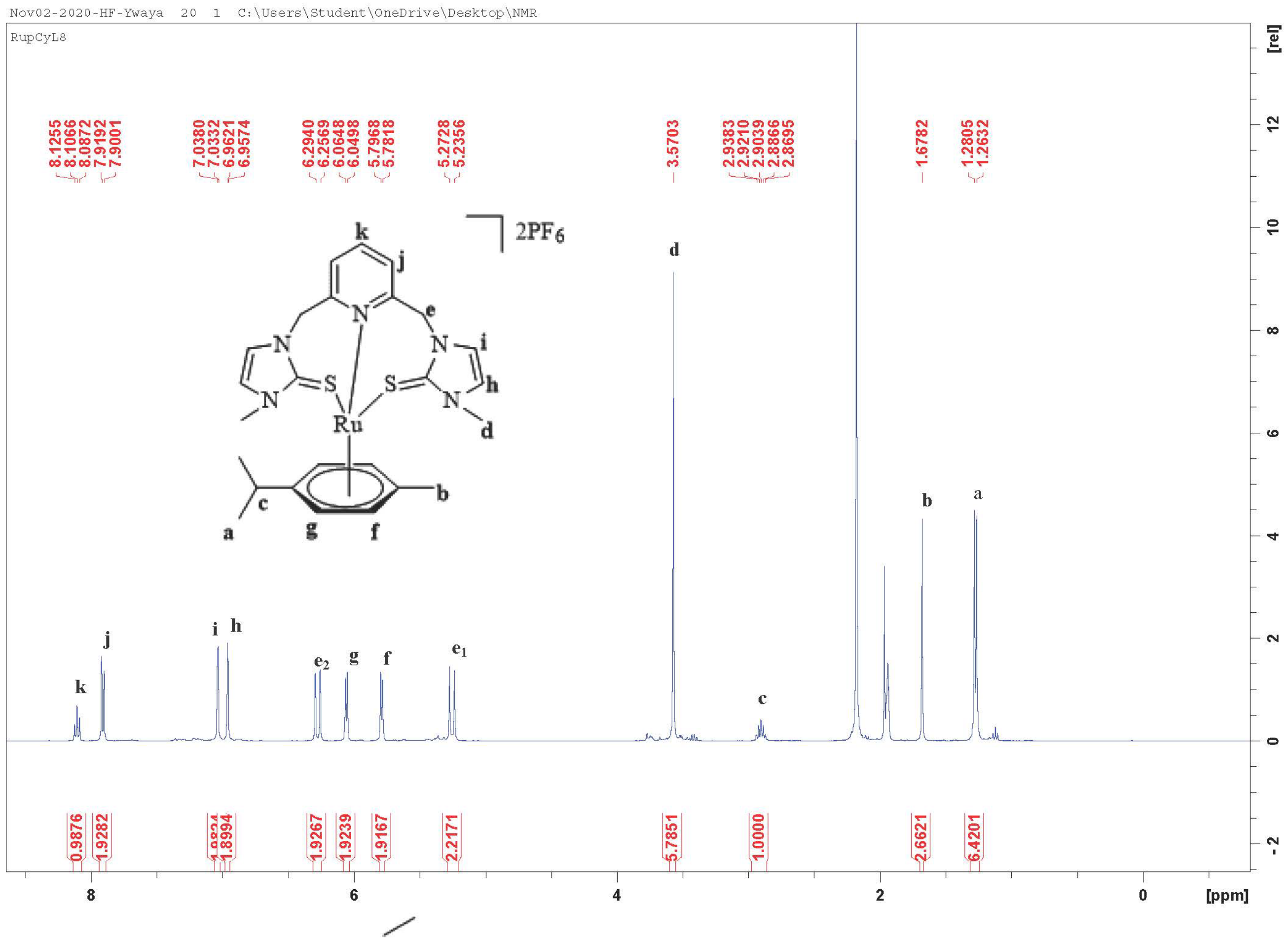
Nov02-2020-HF-Ywaya 20 1 C:\Users\Student\OneDrive\Desktop\NMR
RupCyL8
8.1255
8.1066
8.0872
7.9192
7.9001
7.0380
7.0332
6.9621
6.9574
6.2940
6.2569
6.0648
6.0498
5.7968
5.7818
5.2728
5.2356
3.5703
2.9383
2.9210
2.9039
2.8866
2.8695
1.6782
1.2805
1.2632
2PF6
k
j
e
N
i
h
S
Ru
d
b
c
a
g
f
e2
e1
0.9876
1.9282
1.8994
1.9267
1.9239
1.9167
2.2171
5.7851
1.0000
2.6621
6.4201
[rel]
[ppm]

^{13}C NMR of **4bi**

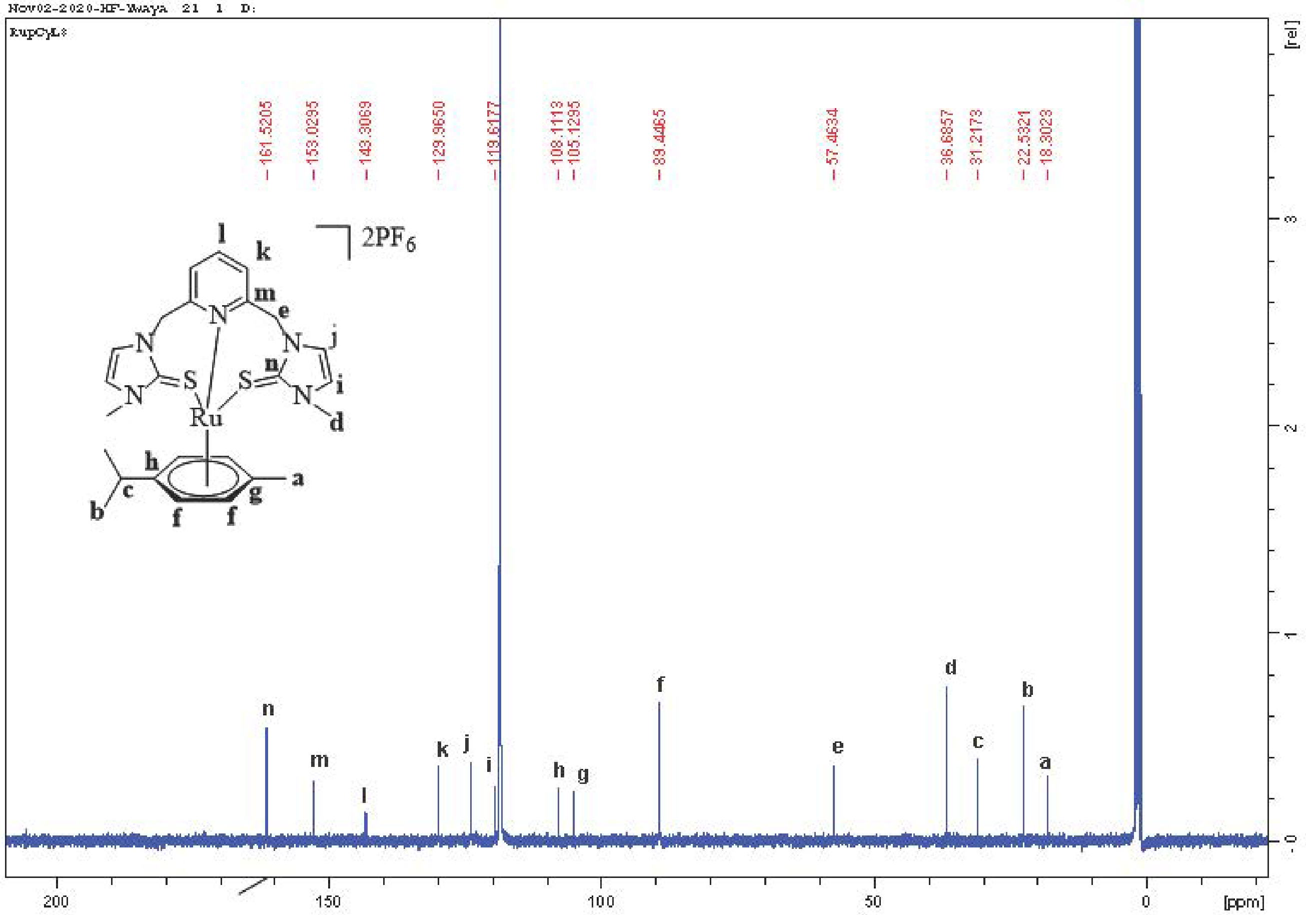

^{1}H NMR of **4bii**

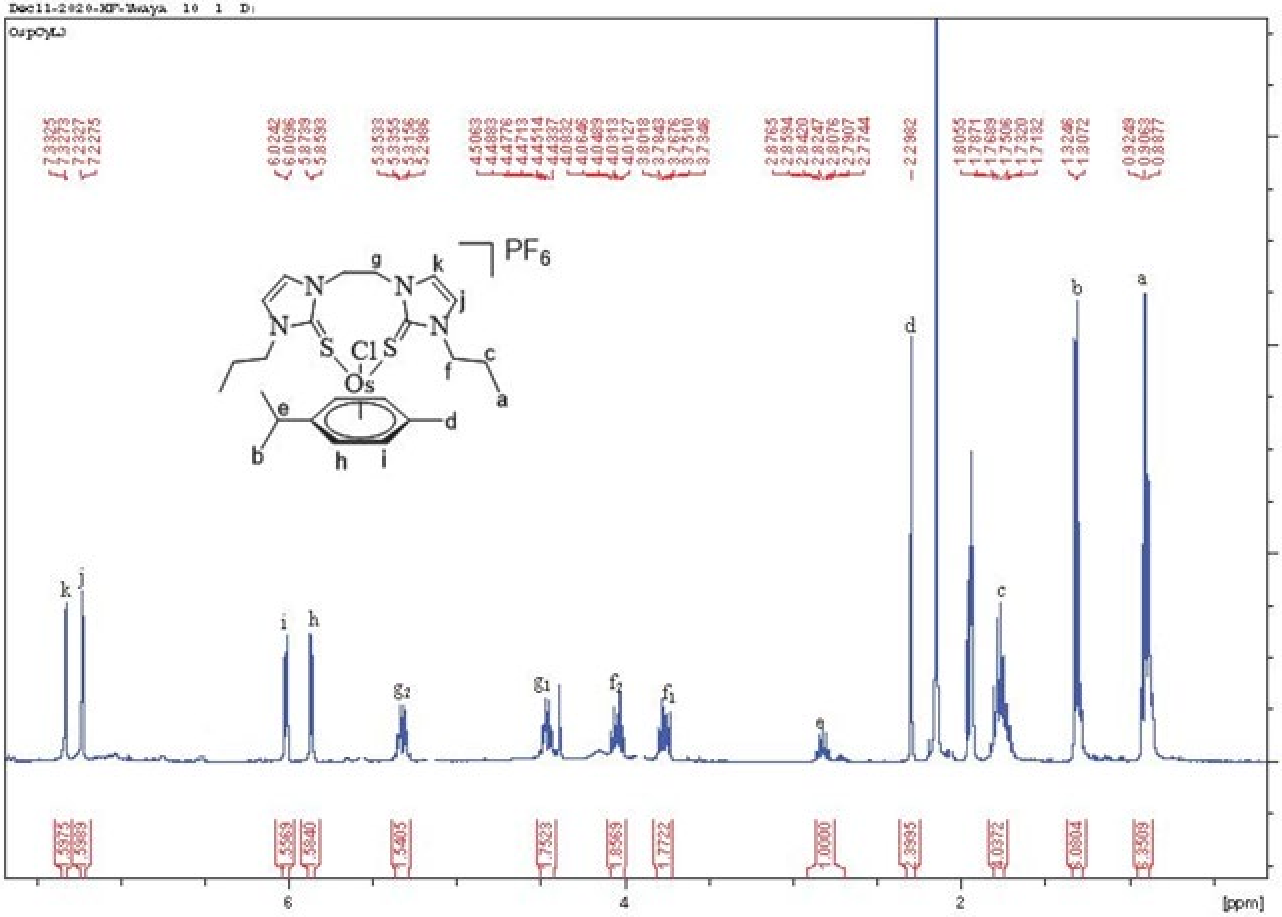

^{13}C NMR of **4bii**

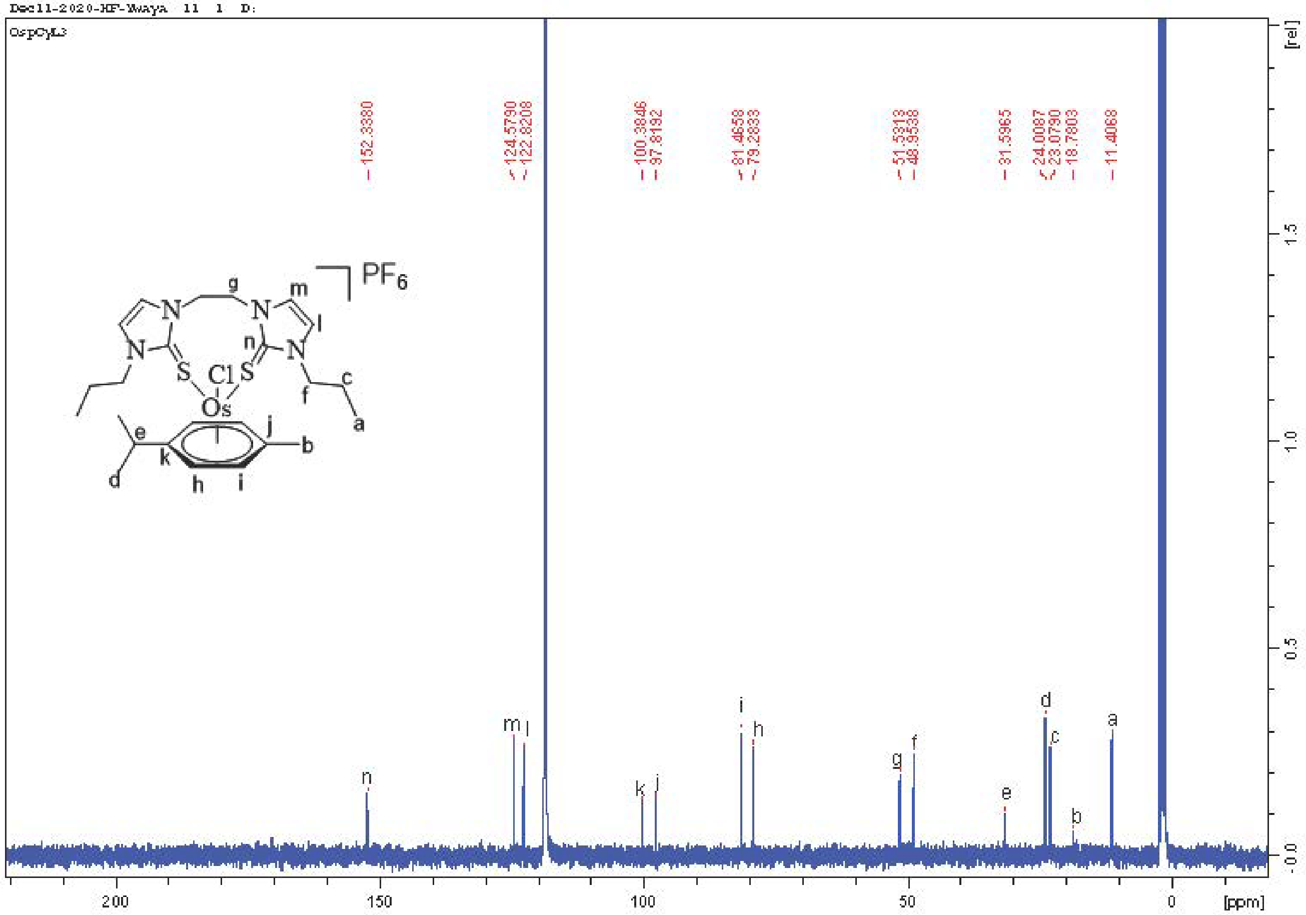

^{1}H NMR **5bi**

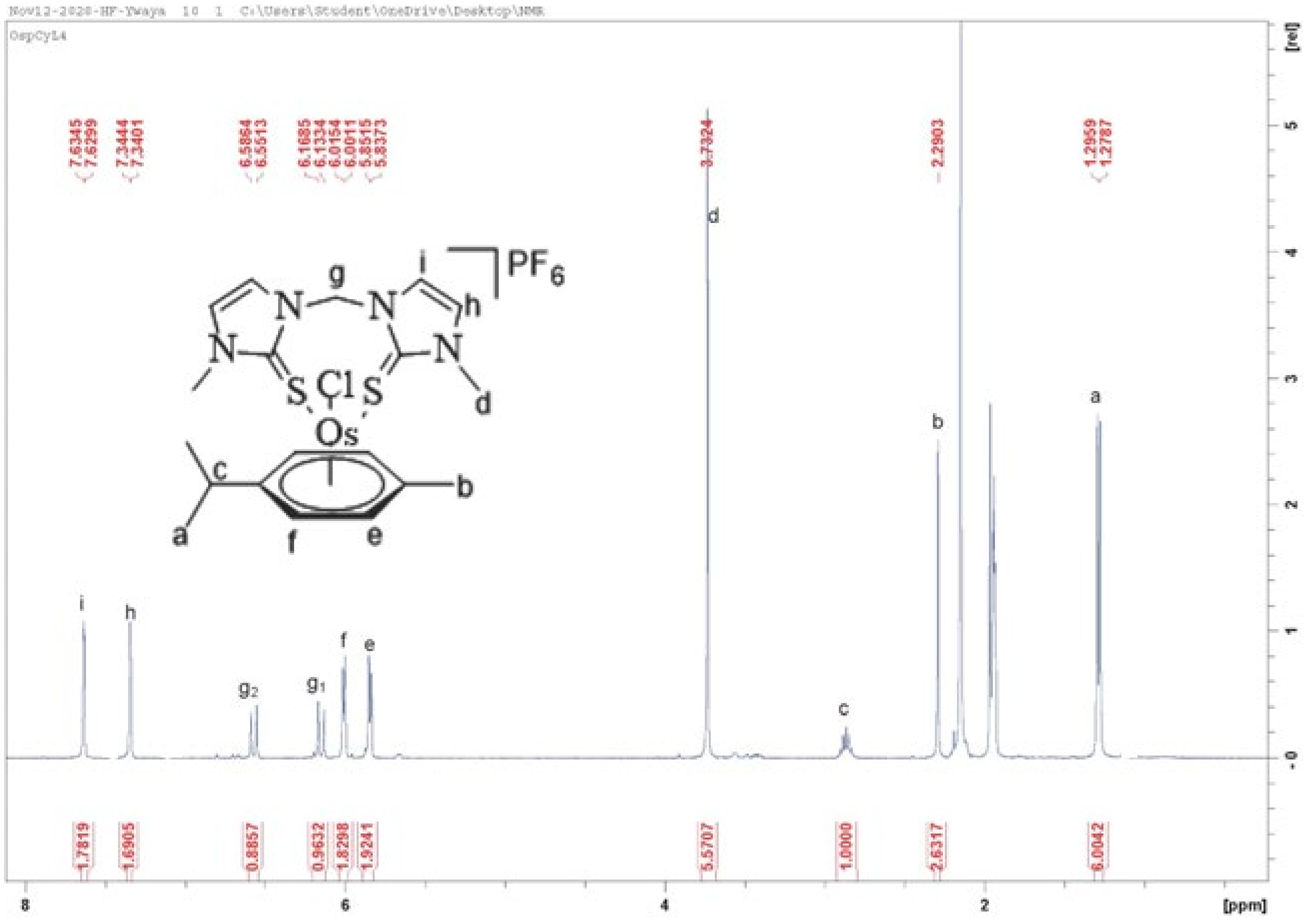

Nov12-2020-HF-Ywaya 10 1 C:\Users\Student\OneDrive\Desktop\NMR
OspCyL4
7.6345
7.6299
7.3444
7.3401
6.5864
6.5513
6.1685
6.1334
6.0154
6.0011
5.8515
5.8373
3.7324
2.2903
1.2959
1.2787
PF6
i
h
g2
g1
f
e
d
c
b
a
1.7819
1.6905
0.8857
0.9632
1.8298
1.9241
5.5707
1.0000
2.6317
6.0042
[rel]
[ppm]

^{13}C NMR of **5bi**

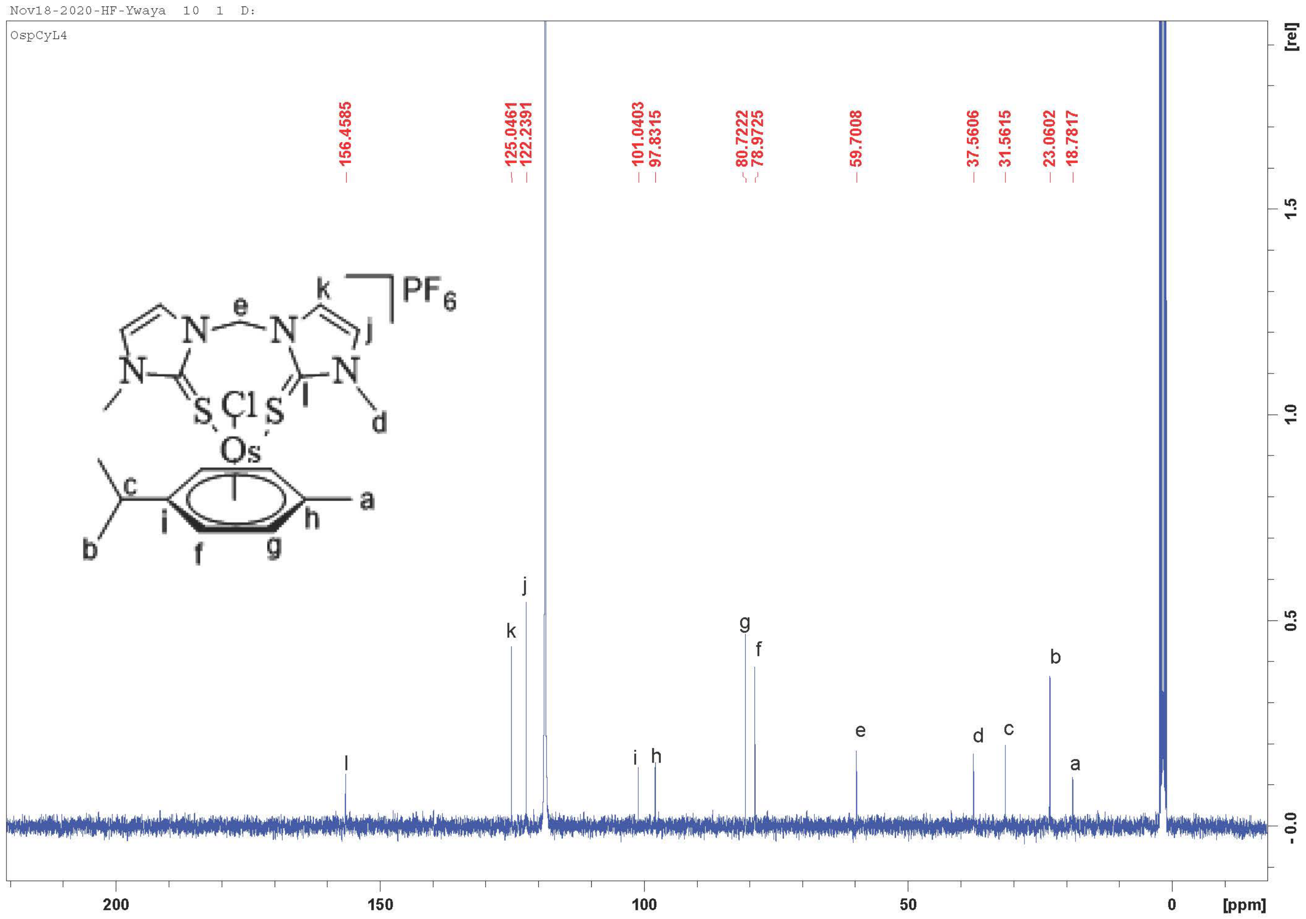

^{1}H NMR of **5civ**

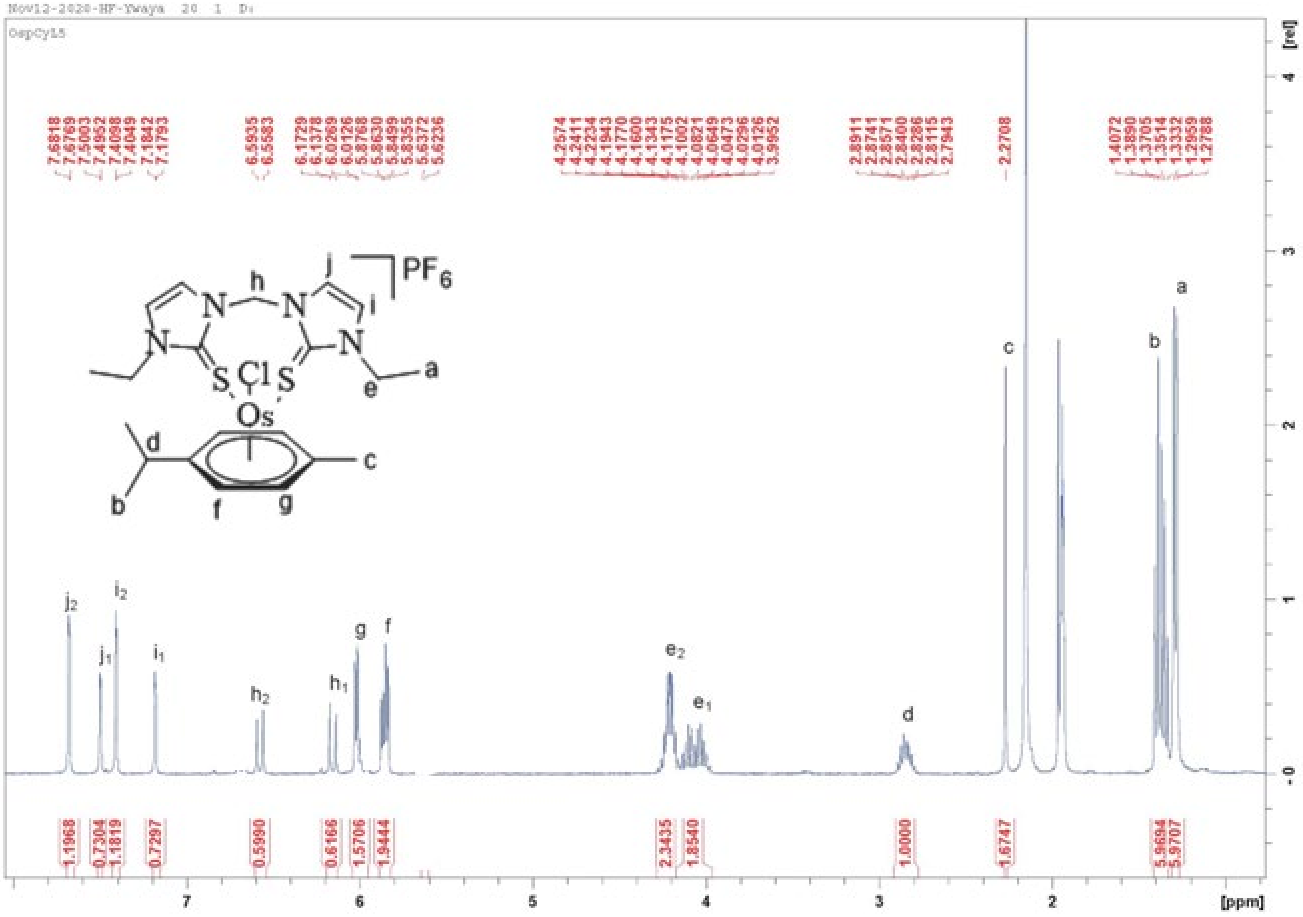

^{13}C NMR of **5civ**

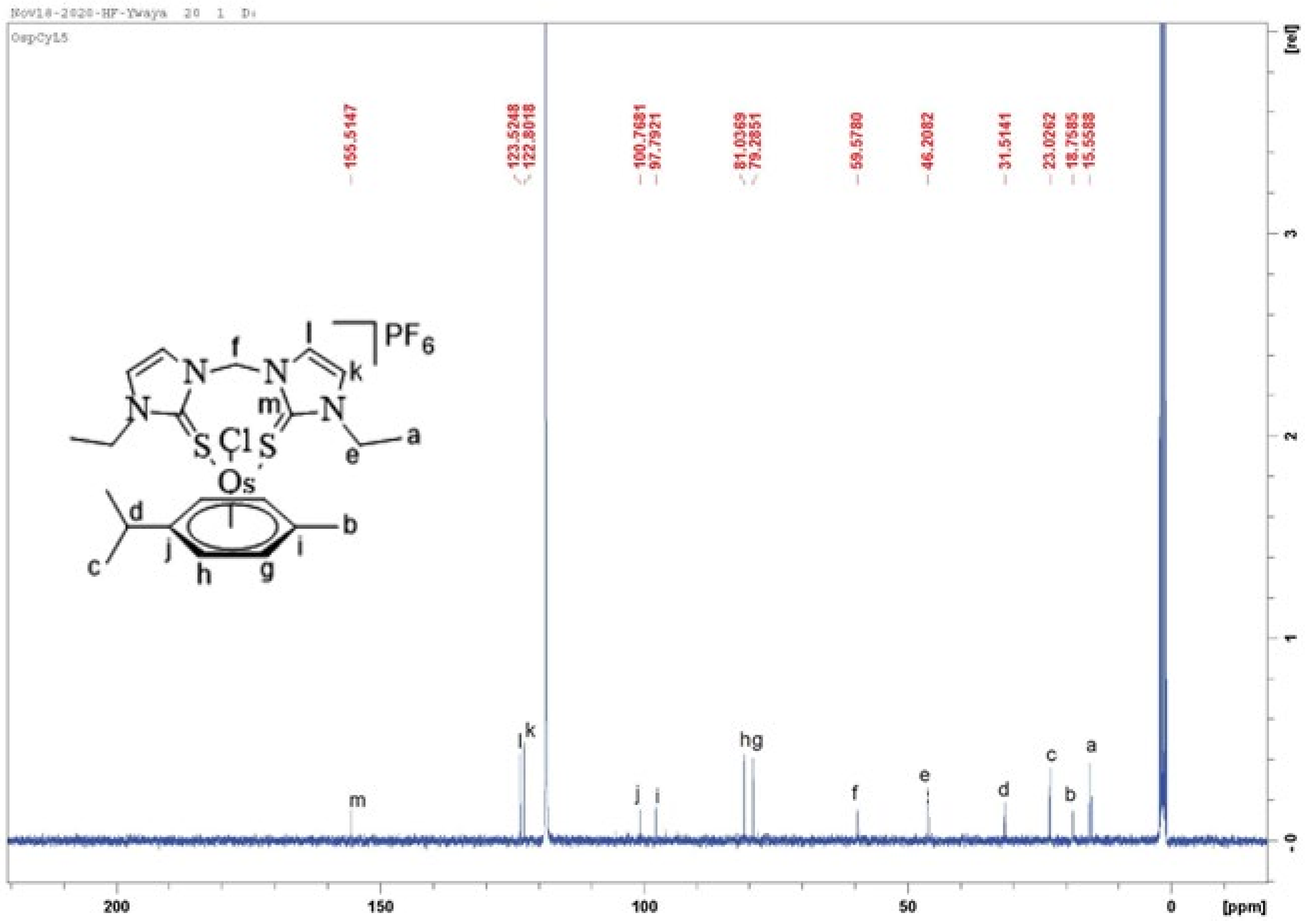

^{1}H NMR of **6bi**

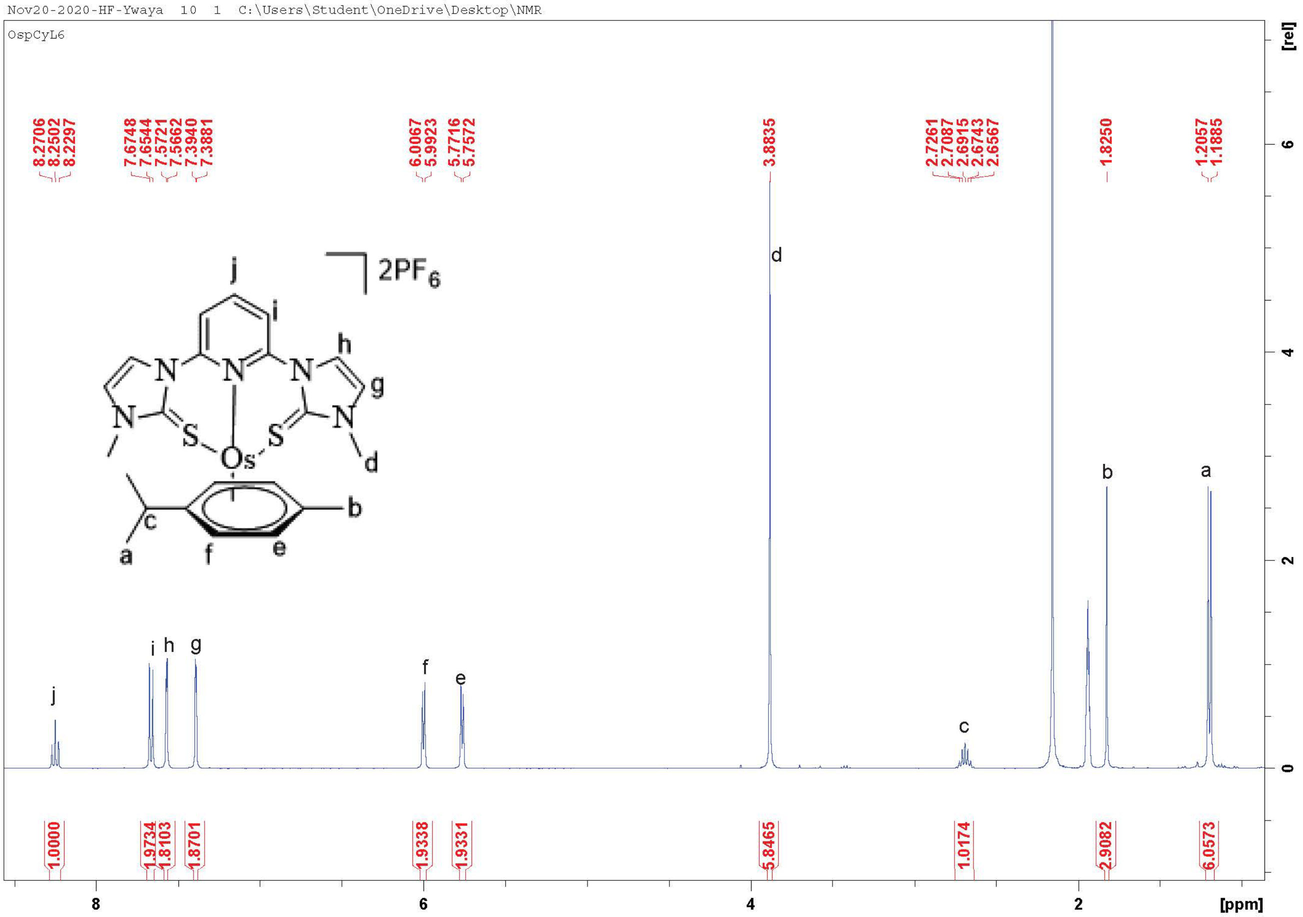

Nov20-2020-HF-Ywaya 10 1 C:\Users\Student\OneDrive\Desktop\NMR
OspCyL6
8.2706
8.2502
8.2297
7.6748
7.6544
7.5721
7.5662
7.3940
7.3881
6.0067
5.9923
5.7716
5.7572
3.8835
2.7261
2.7087
2.6915
2.6743
2.6567
1.8250
1.2057
1.1885
2PF6
j
i
h
g
d
c
b
a
f
e
1.0000
1.9734
1.8103
1.8701
1.9338
1.9331
5.8465
1.0174
2.9082
6.0573
[rel]
[ppm]

^{13}NMR of **6bi**

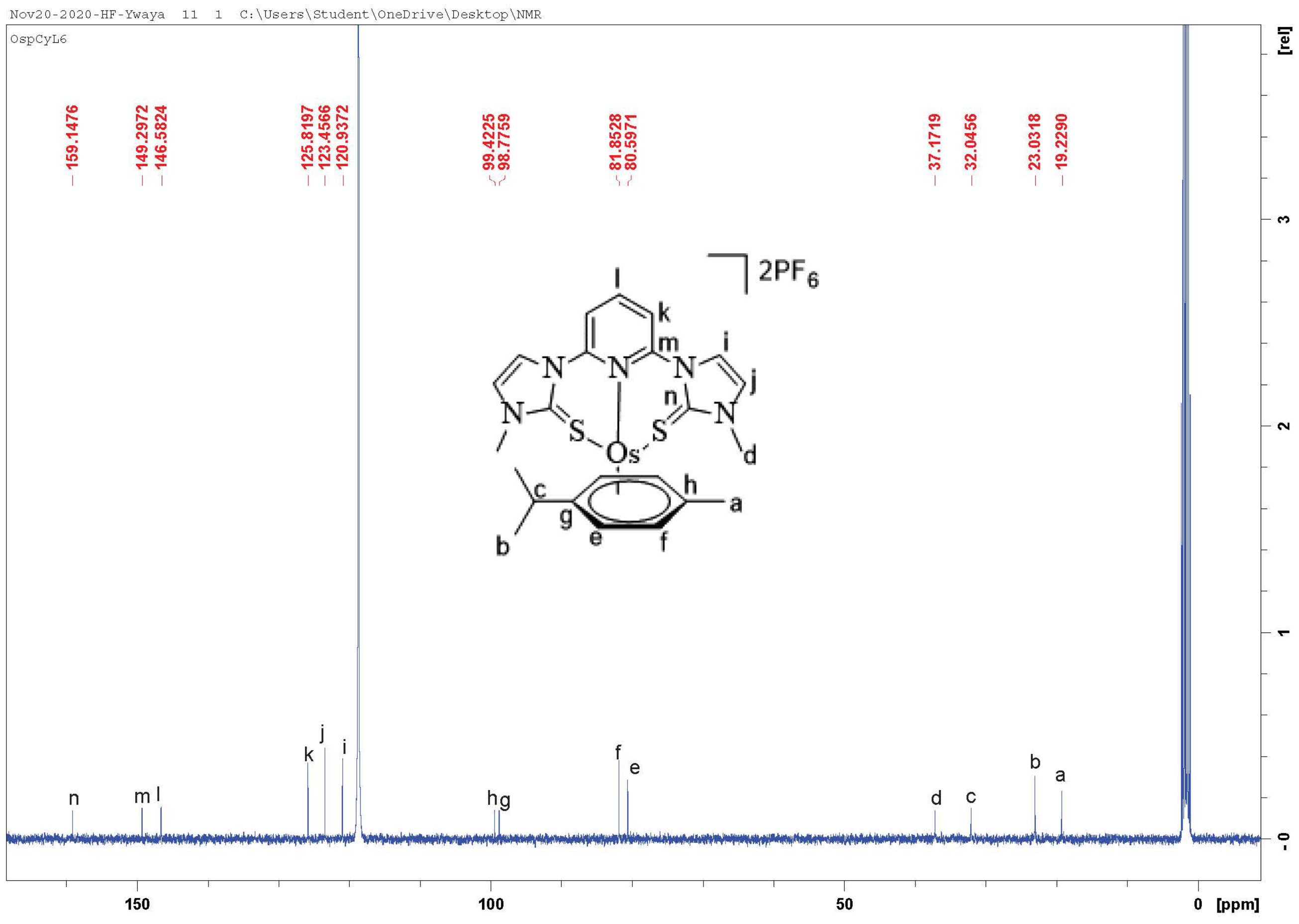

Nov20-2020-HF-Ywaya 11 1 C:\Users\Student\OneDrive\Desktop\NMR
OspCyL6
159.1476
149.2972
146.5824
125.8197
123.4566
120.9372
99.4225
98.7759
81.8528
80.5971
37.1719
32.0456
23.0318
19.2290
2PF6
n
m
l
k
j
i
h
g
f
e
d
c
b
a
150
100
50
0
[ppm]
[rel]

^{1}H NMR of **6ci**

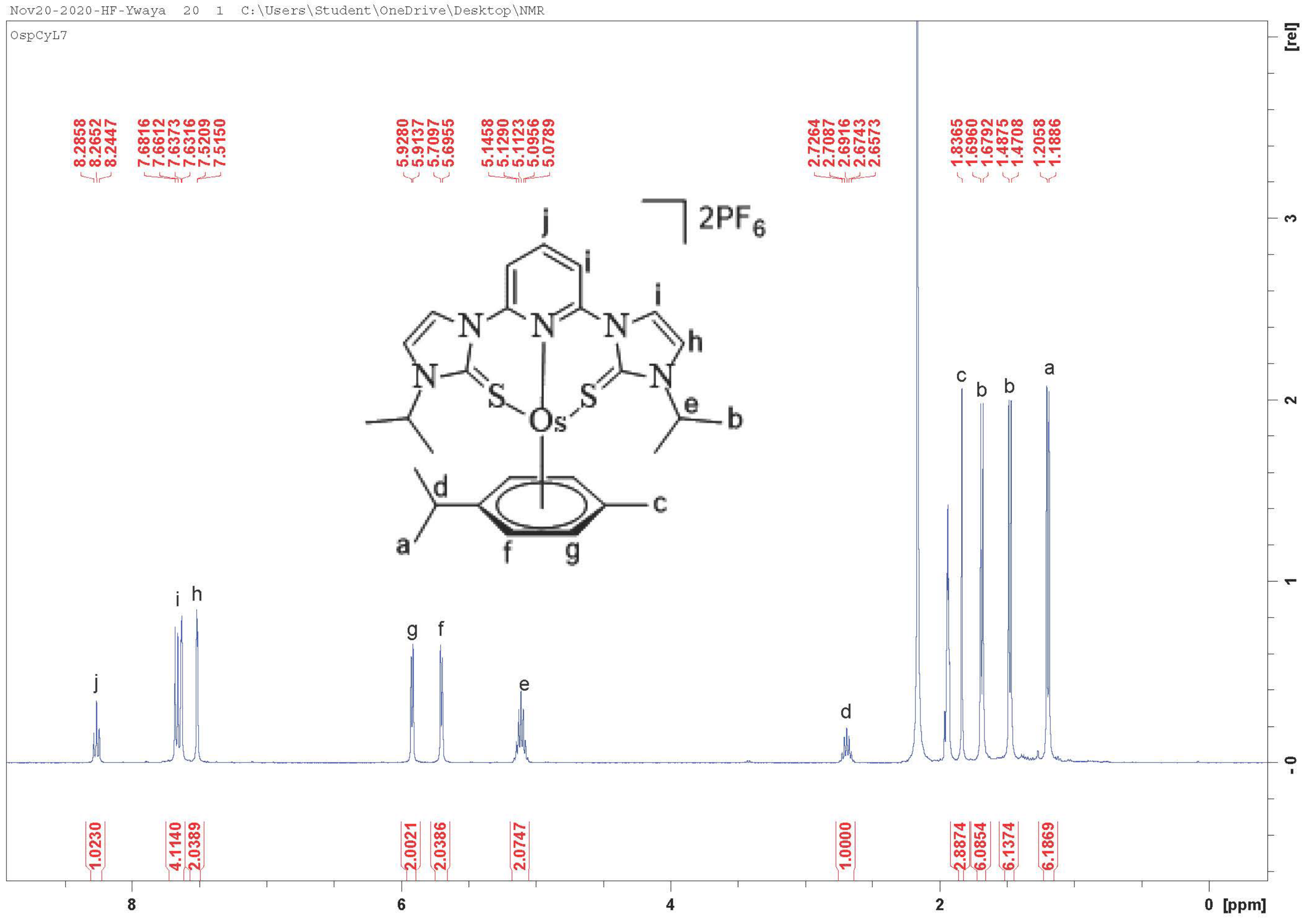
Nov20-2020-HF-Ywaya 20 1 C:\Users\Student\OneDrive\Desktop\NMR
OspCyL7
8.2858
8.2652
8.2447
7.6816
7.6612
7.6373
7.6316
7.5209
7.5150
5.9280
5.9137
5.7097
5.6955
5.1458
5.1290
5.1123
5.0956
5.0789
2.7264
2.7087
2.6916
2.6743
2.6573
1.8365
1.6960
1.6792
1.4875
1.4708
1.2058
1.1886
2PF6
j
i
h
e
b
d
c
a
f
g
N
S
Os
1.0230
4.1140
2.0389
2.0021
2.0386
2.0747
1.0000
2.8874
6.0854
6.1374
6.1869
8
6
4
2
0 [ppm]
[rel]

^{13}C NMR of **6ci**

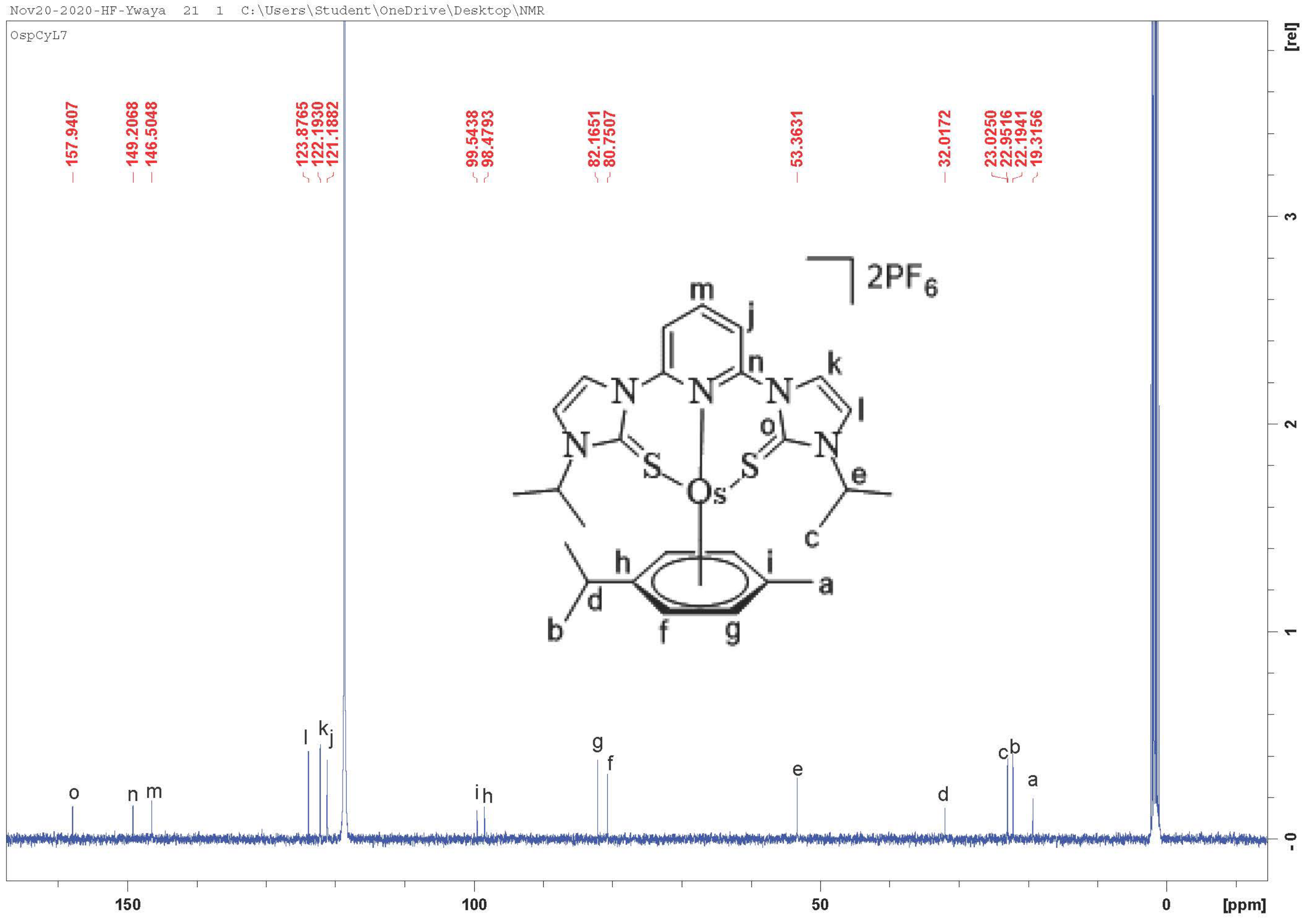

^{1}H NMR of **6civ**

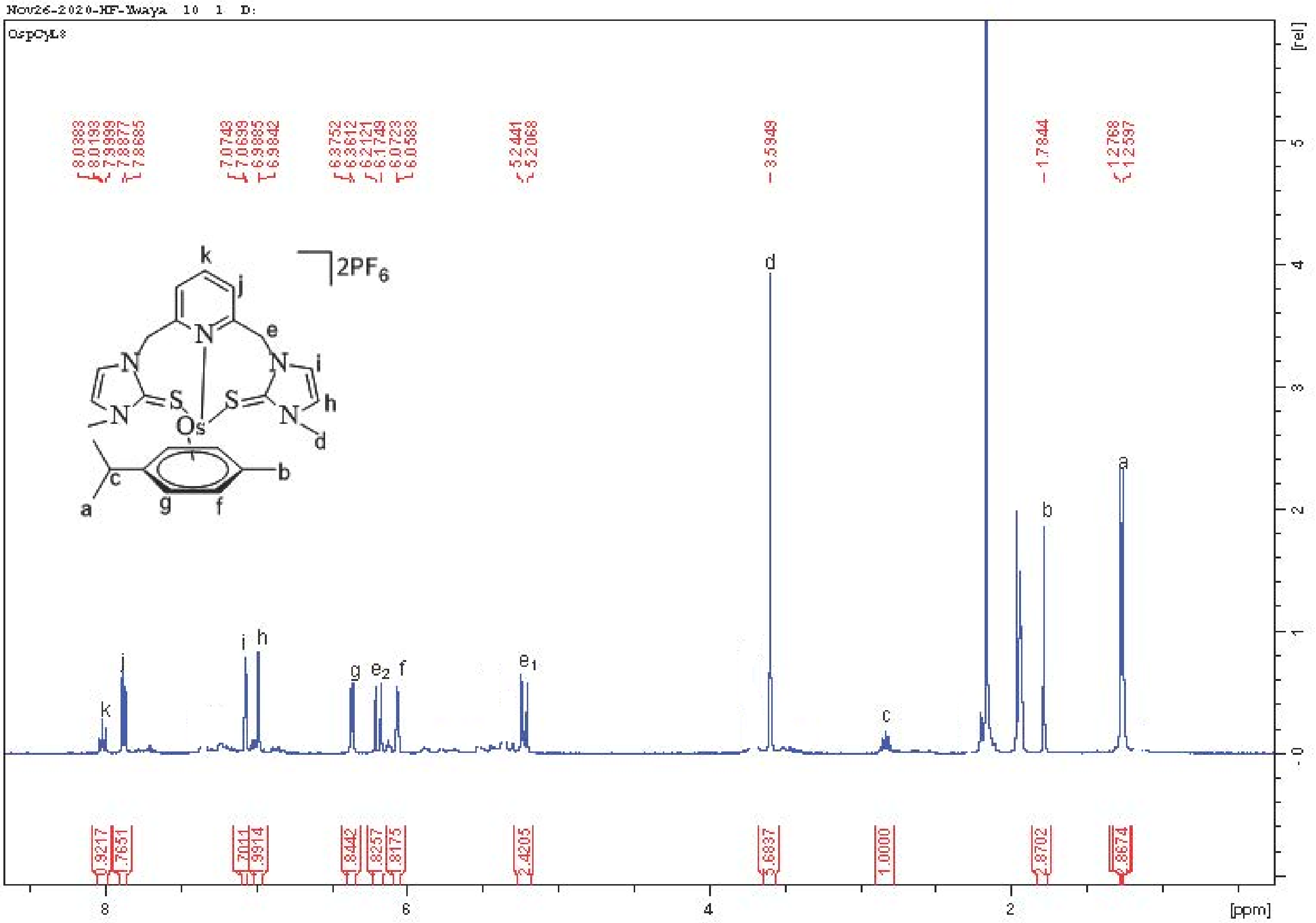

^{13}C NMR of **6civ**

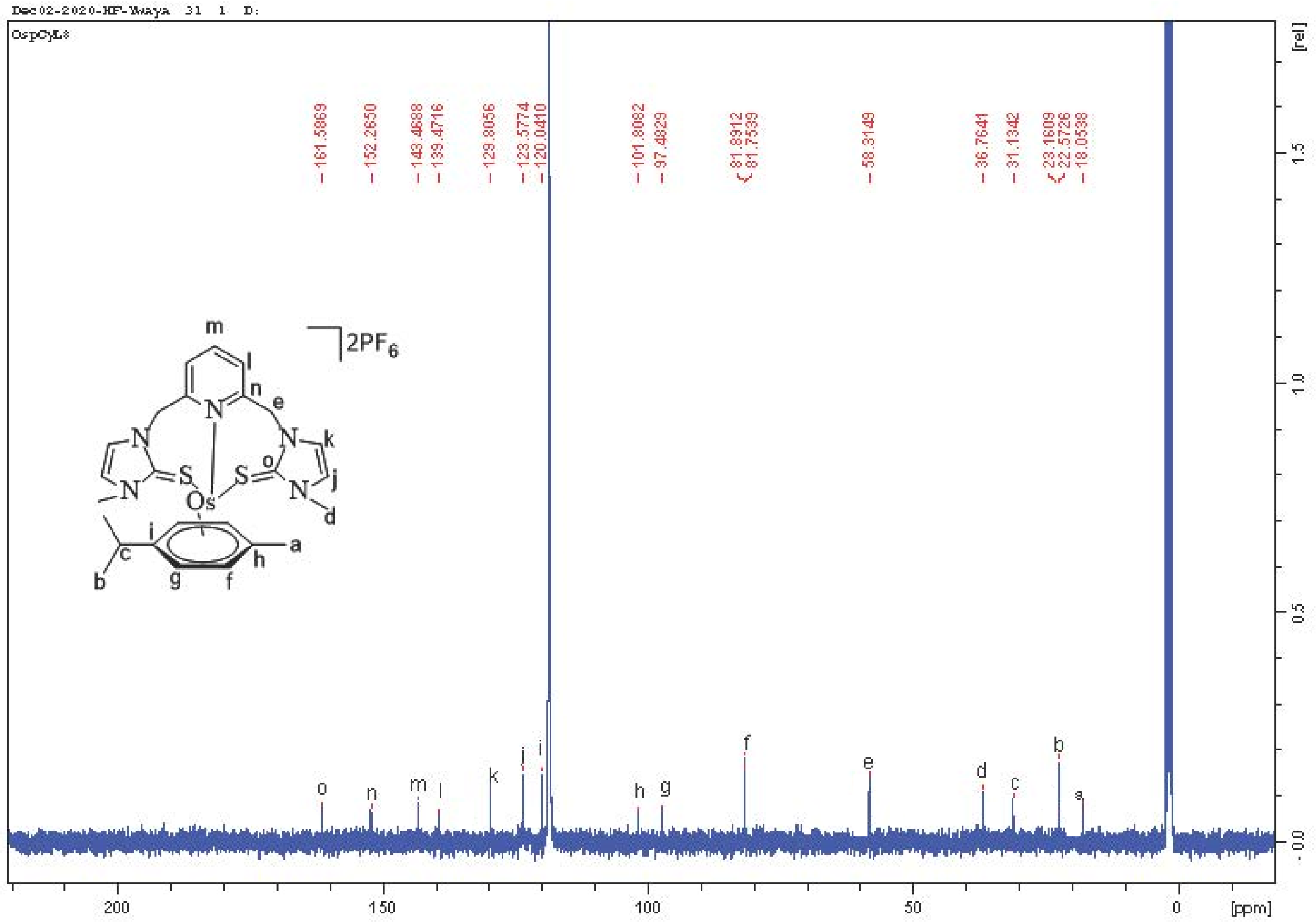

HR-MS of **3ai**

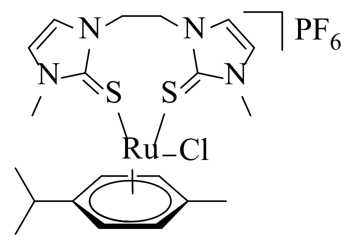

Mol. Wt. [M] = 670.0803

Found for $[M\text{-}PF_6]^{2+}$ = 538.0687; calculated = 539.0751

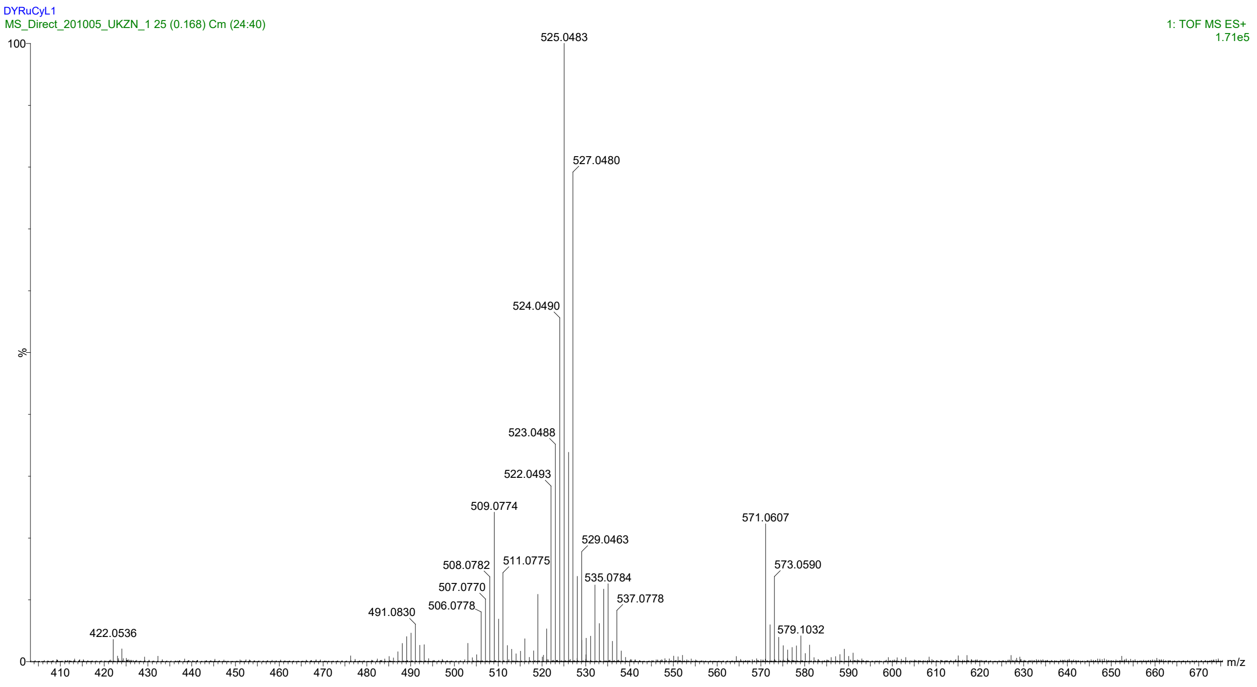

HR-MS of **3aii**

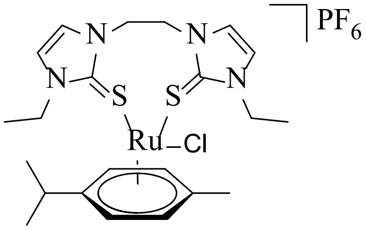

Mol. wt [M] = 698.1335

Found $[M-PF_6]^+$ = 553.0801

Calculated $[M-PF_6]^+$ = 555.0690

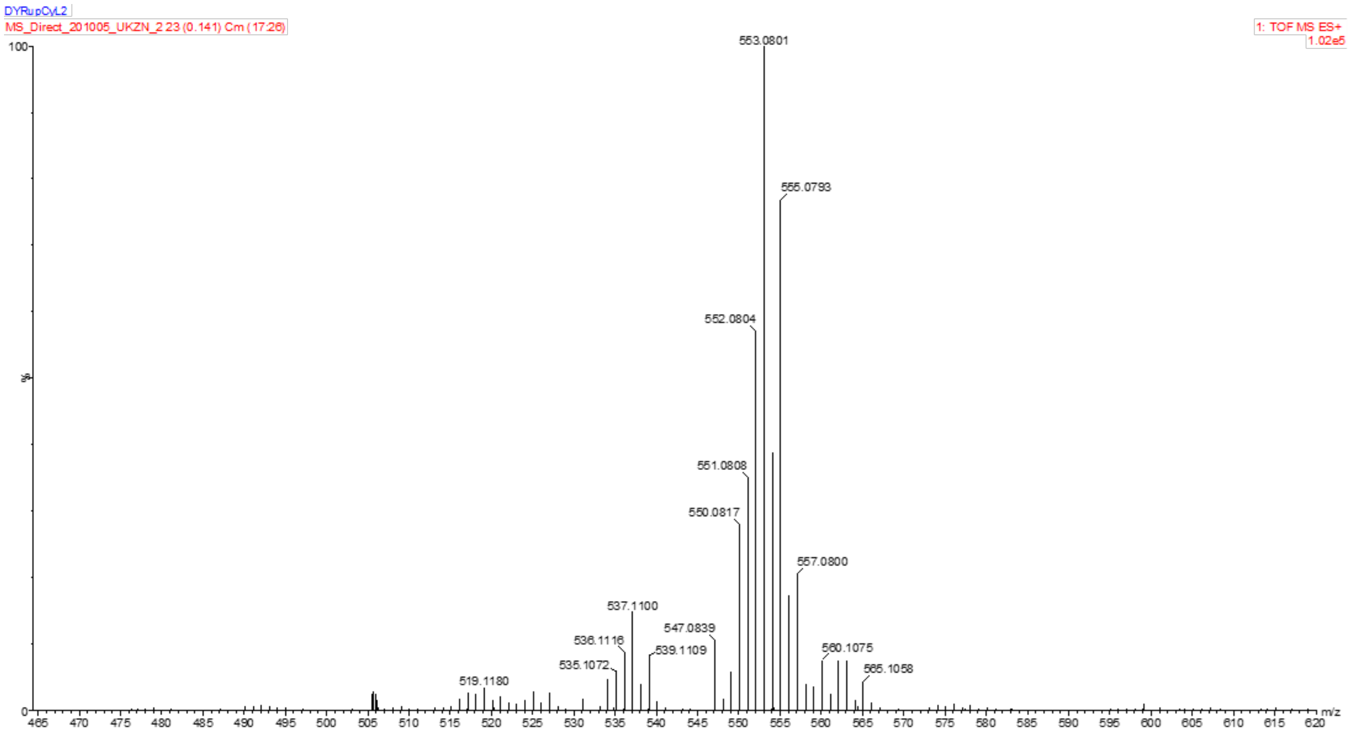

HR-MS of **3aii**

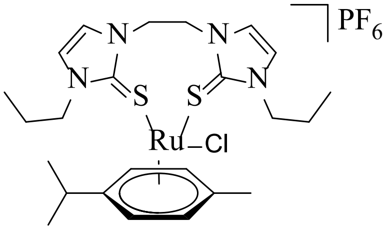

Mol. wt [M] = 726.1866

Found $[M\text{-}PF_6]^+$ = 581.1107

Calculated $[M\text{-}PF_6]^+$ = 581.1003

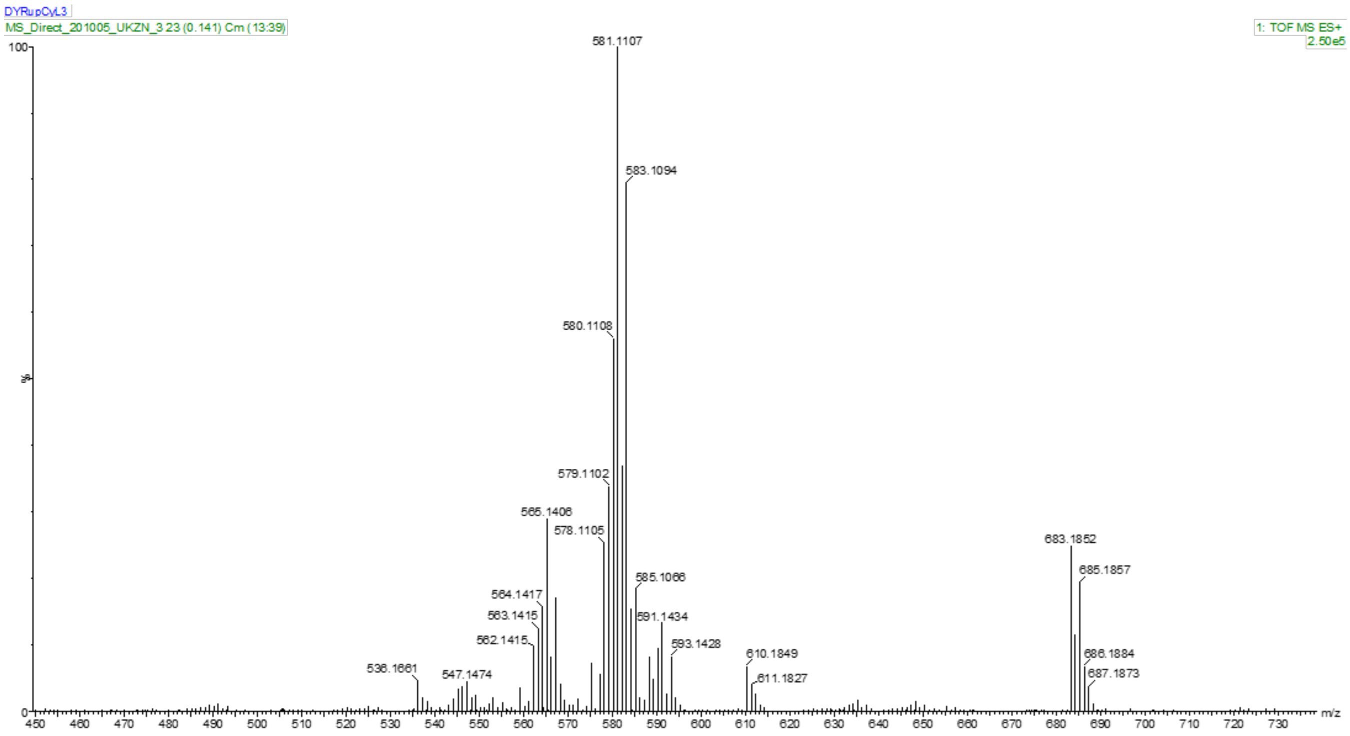

HR-MS of **3bii**

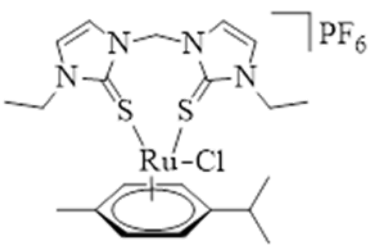

Mol. Wt. [M] = 684.1069
Found [M-PF_6] = 539.0646
Calculated [M-PF_6] = 539.0644

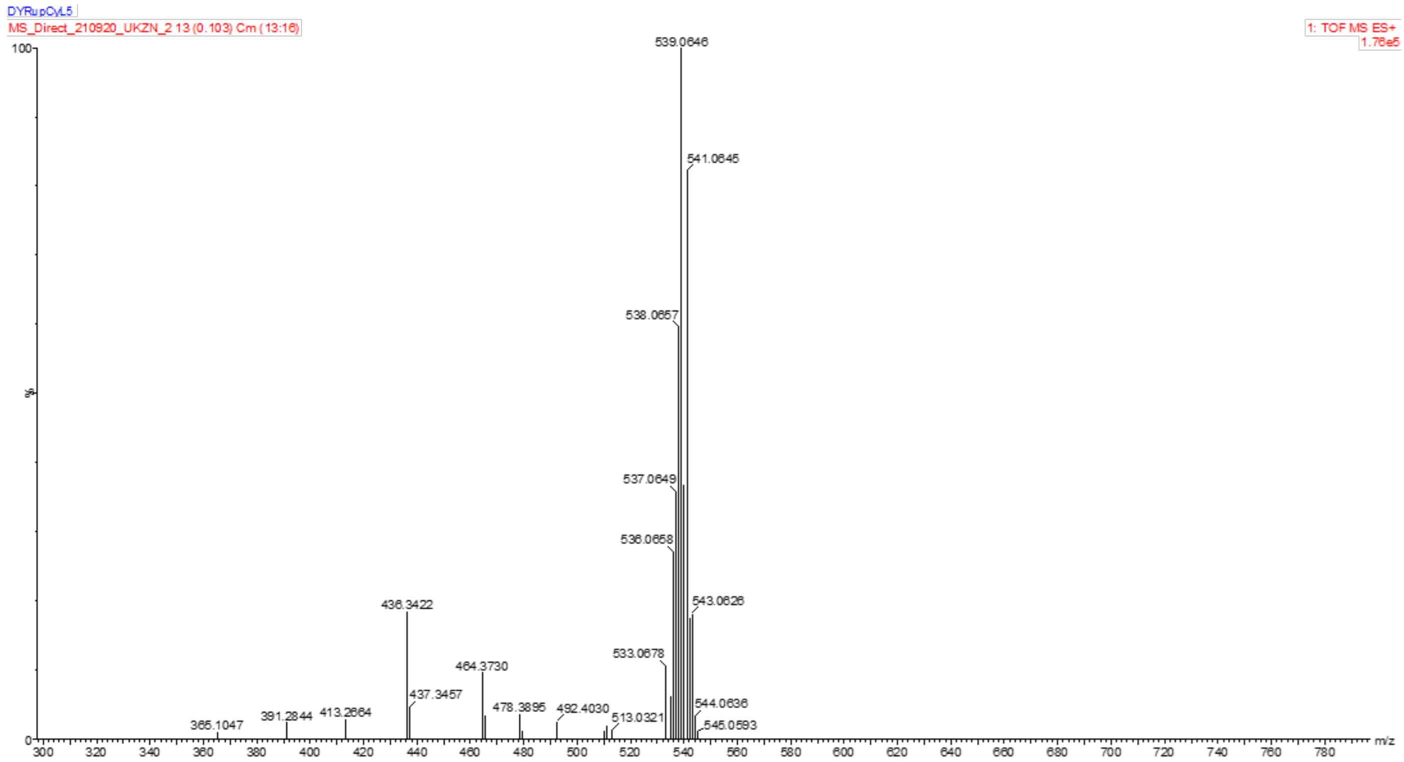

HR-MS of **4aiii**

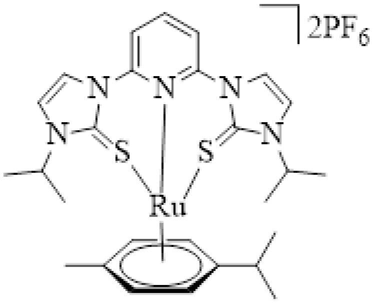

Mol. Wt. [M] = 884.7289
Found $[M-PF_6]^{2+}$ = 740.1014
Calculated $[M-PF6]^{2+}$ = 740.1019
Found $[M+Li]^{2+}$ = 892.0860
Calculated $[M+Li]^{2+}$ = 892.0821

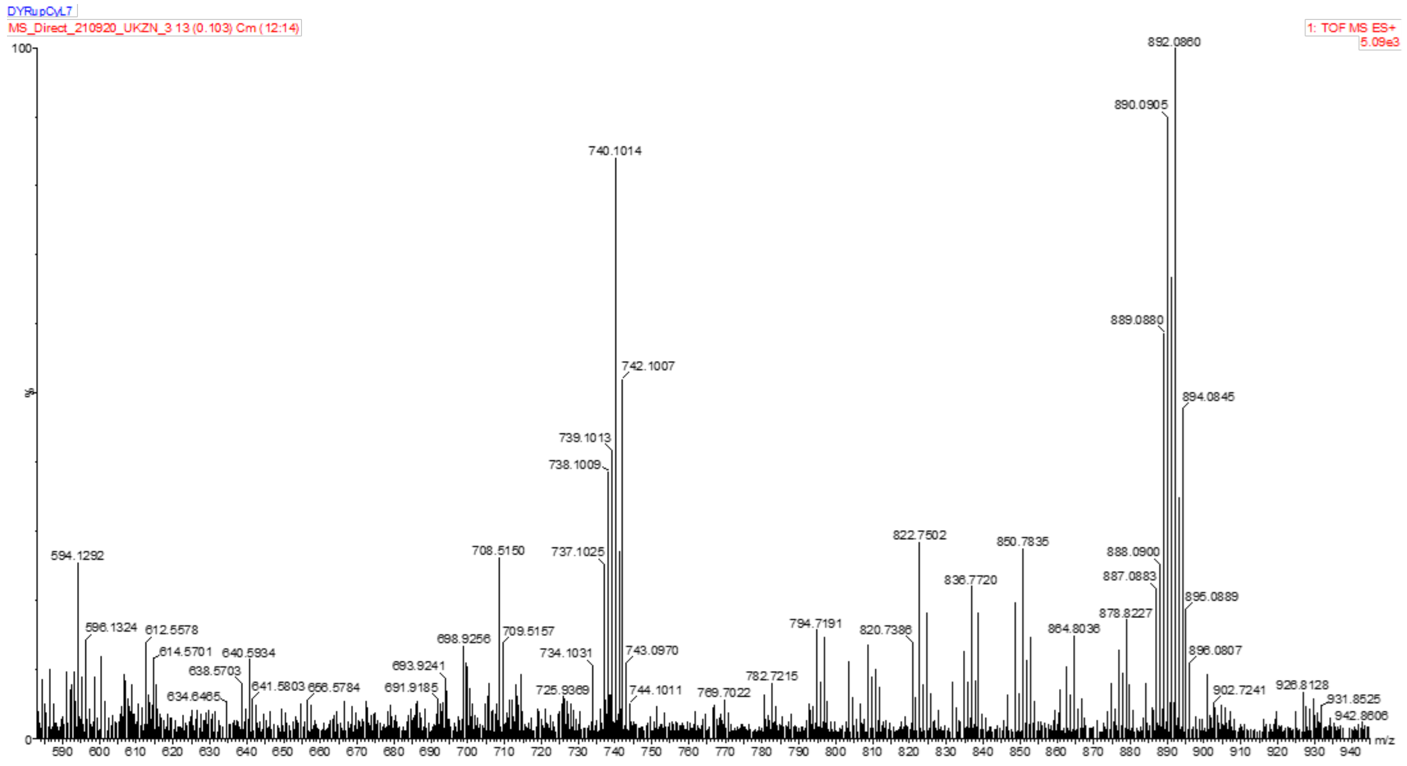

HR-MS of 4bi

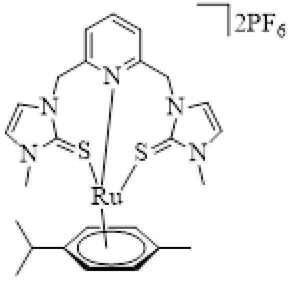

Mol. Wt. [M] = 856.6755

Found for $[M\text{-}2PF_6\text{-}2CH_3]^{2+}$ = 538.0687; calculated = 539.0751

Found for $[M\text{-}PF_6\text{-}2CH_3]^{2+}$ = 684.0386; calculated = 684.0393

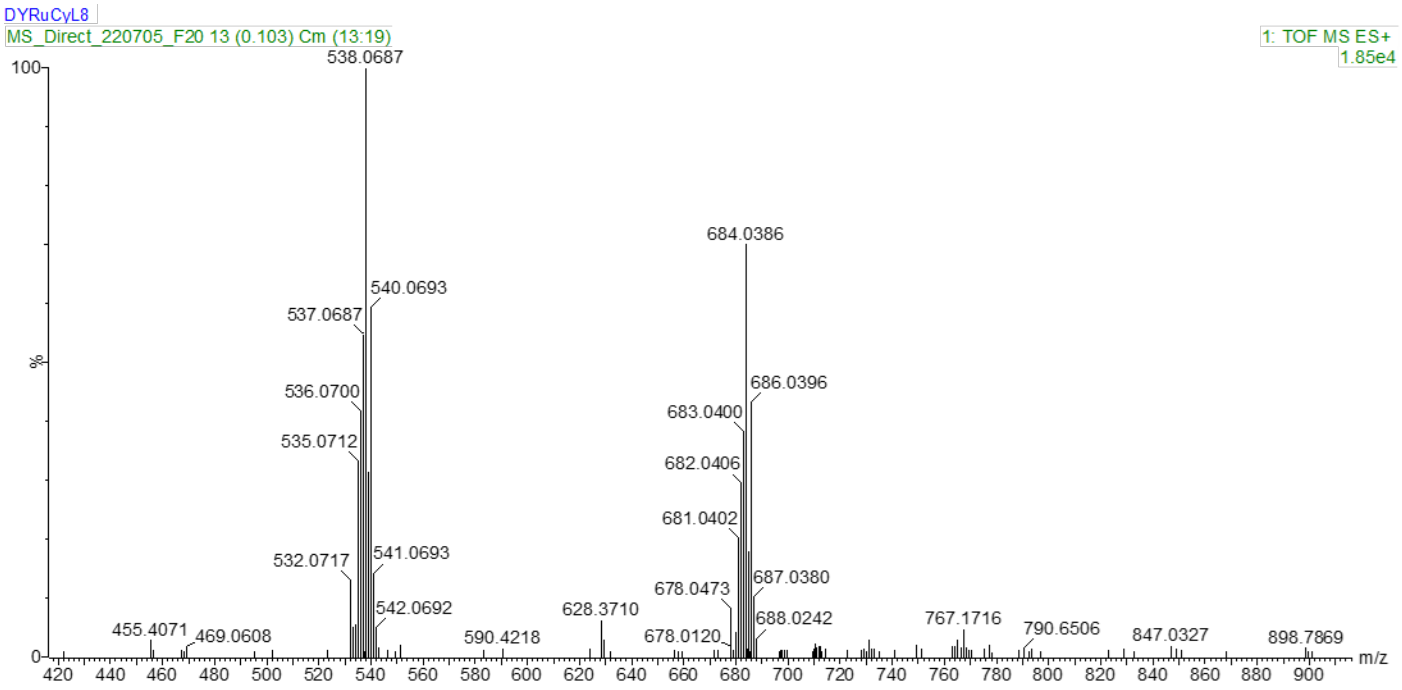

HR-MS of **4bii**

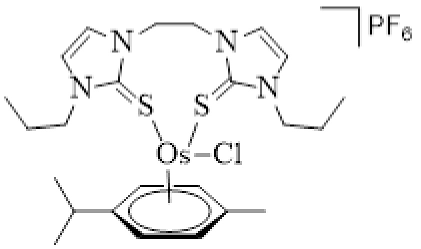

Mol. Wt. [M] = 815.3466
Found $[M-PF_6-Cl]^{2+}$ = 635.1862
Calculated $[M-PF_6-Cl]^{2+}$ = 635.4136

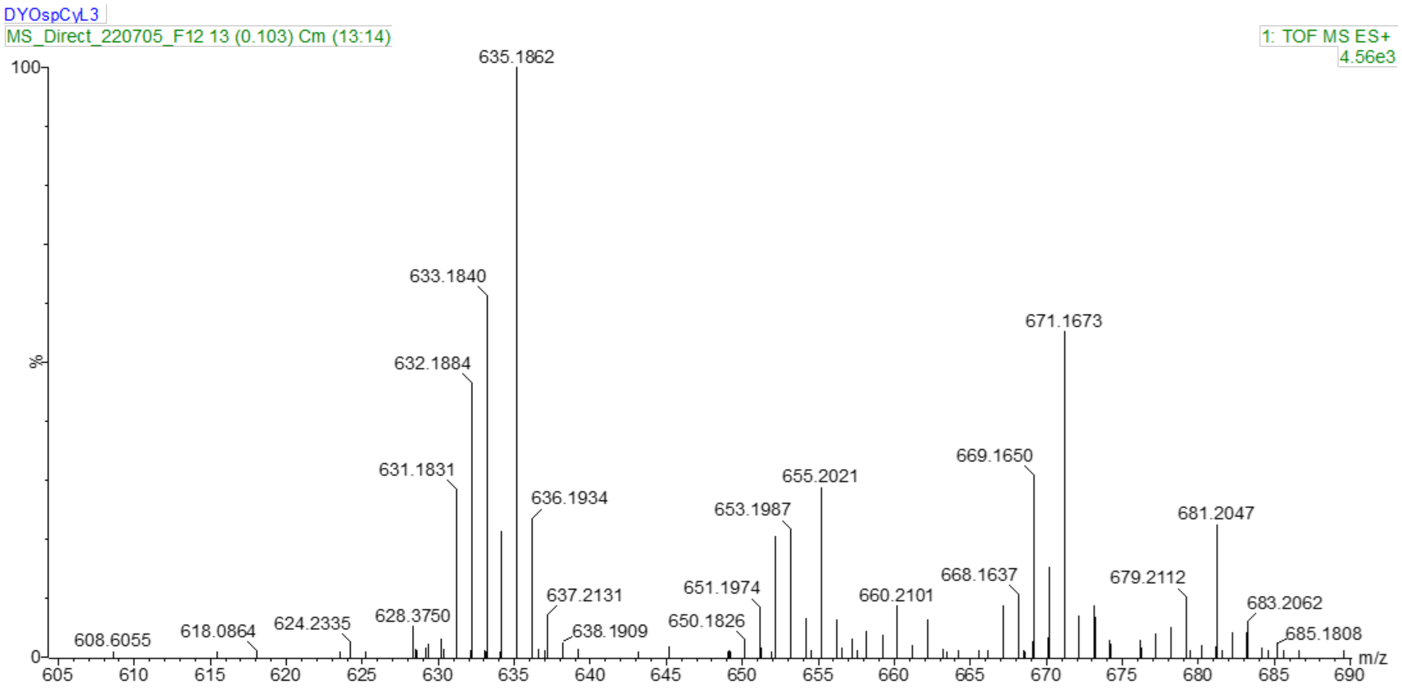

HR-MS of 5bi

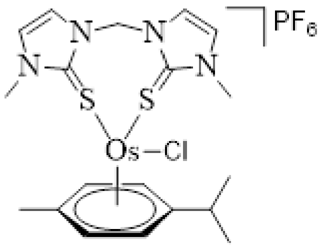

Mol. Wt. [M] = 745.2137
Found $[M-PF_6]^{2+}$ = 601.0878
Calculated $[M-PF_6]^{2+}$ = 601.0902

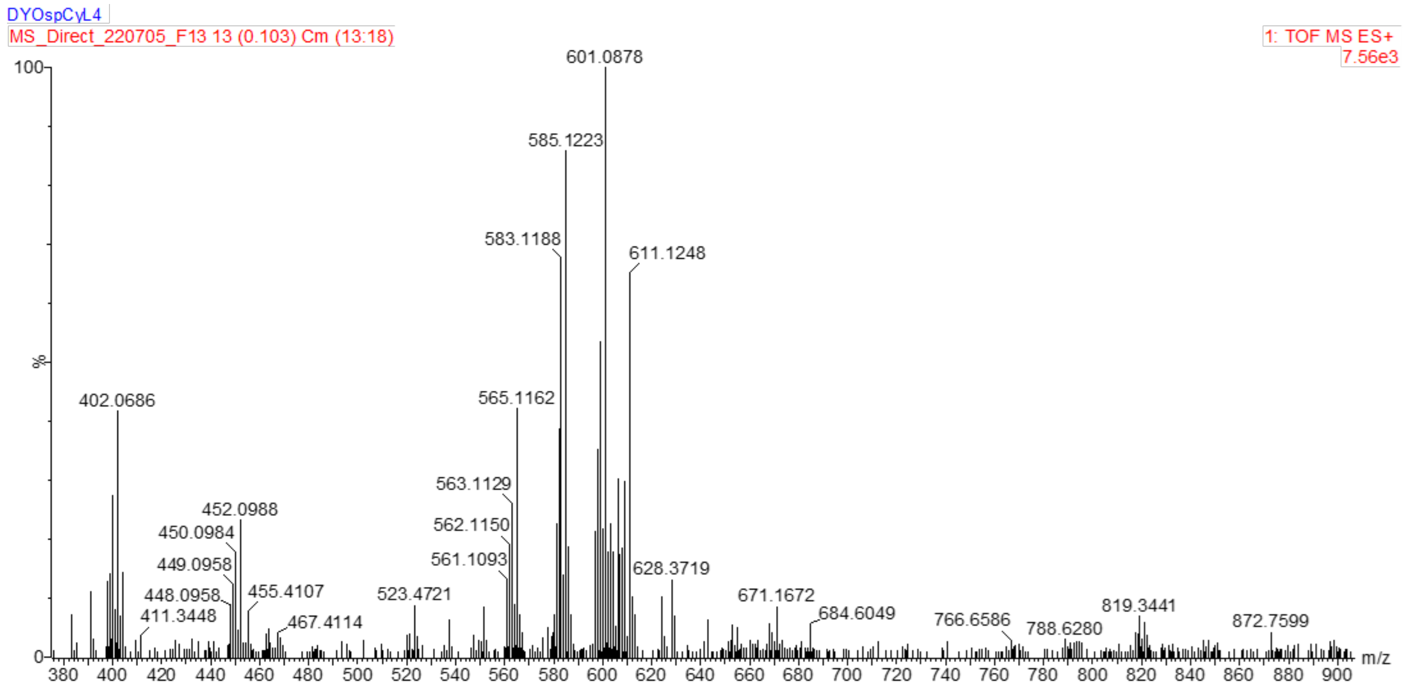

HR-MS 5civ

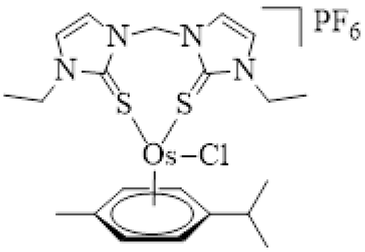

Mol. Wt. [M] = 773.2669

Found: $[M - PF_6 - Cl]^{2+}$=593.1472

Calculated: $[M - PF_6 - Cl]^{2+}$ = 594.1527

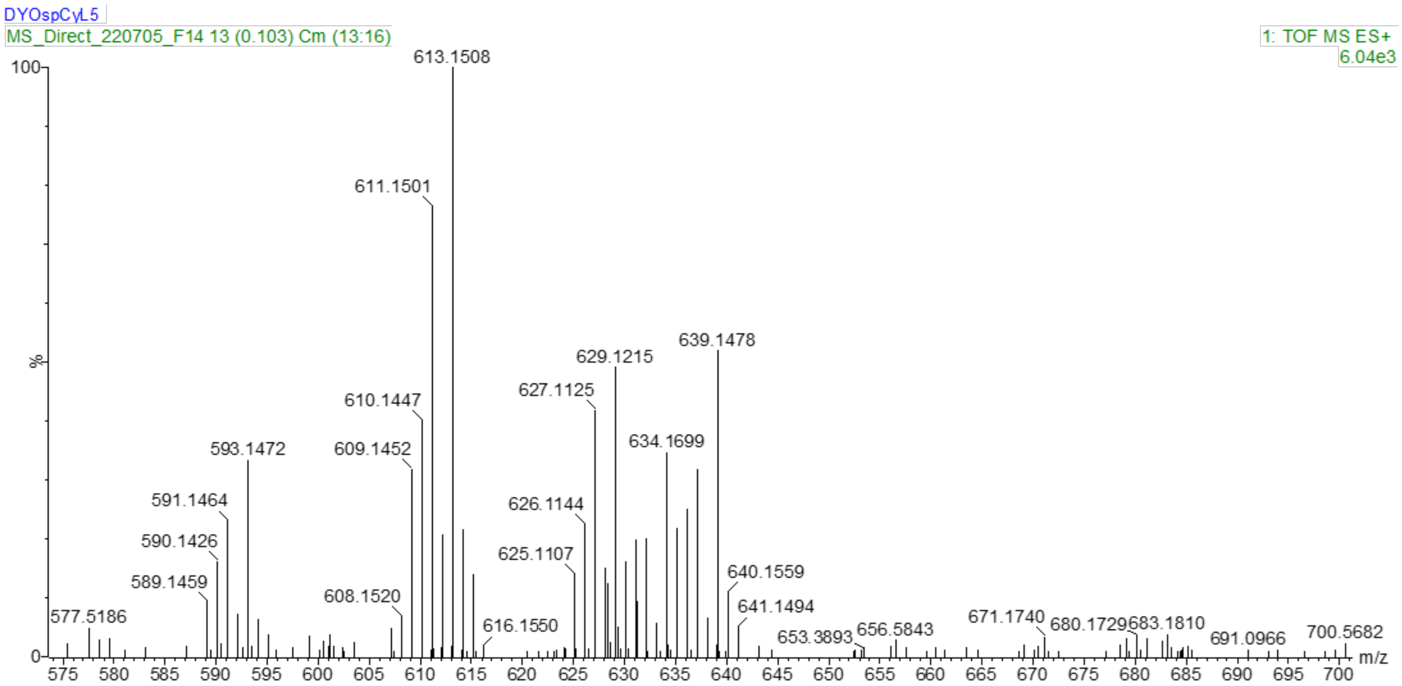

HR-MS of **6bi**

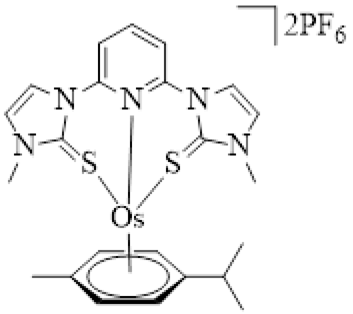

Mol. Wt. [M] = 917.7823
Found $[M-PF_6]^{2+}$ = 772.0970
Calculated $[M-PF_6]^{2+}$ = 772.0934

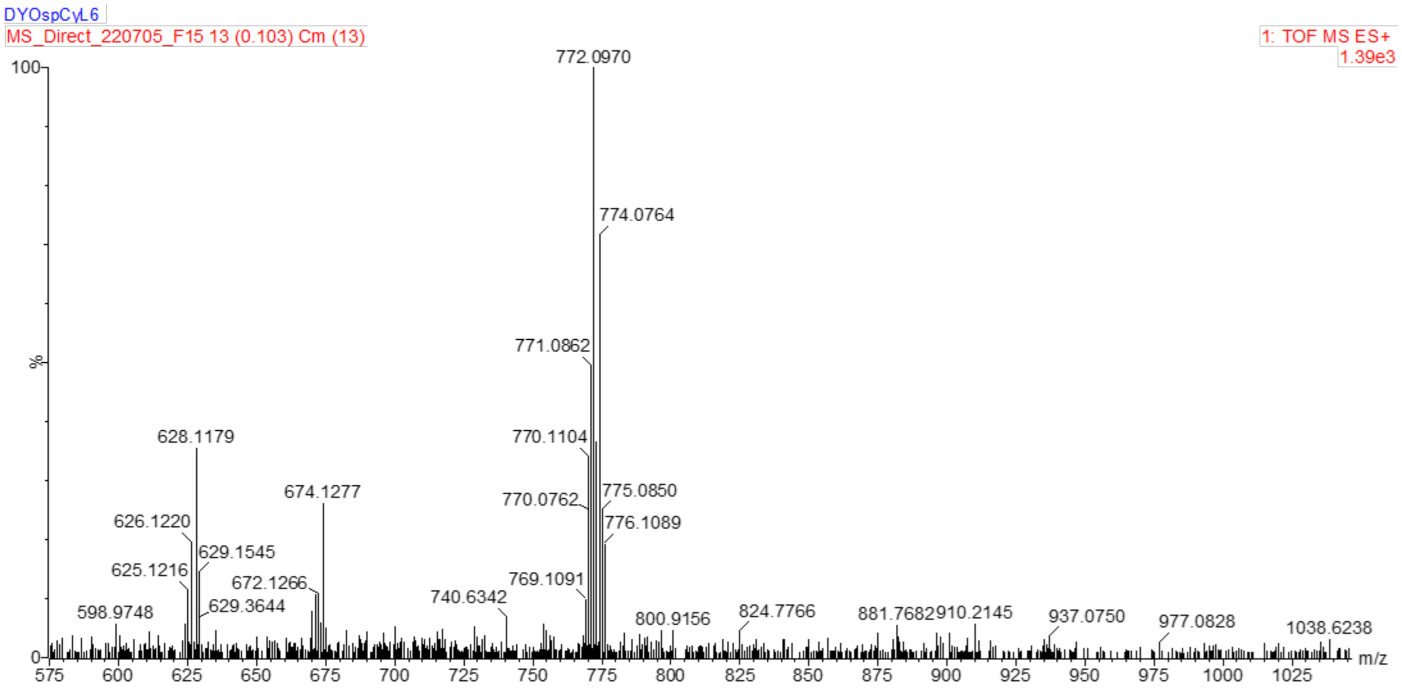

HR-MS of **6ci**

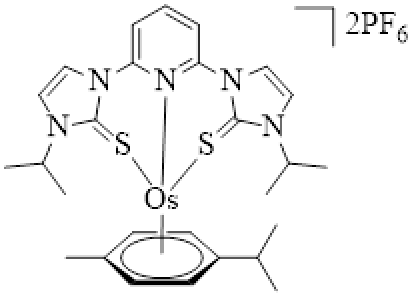

Mol. Wt. [M] = 973.8887
Found $[M\text{-}PF_6]^{2+}$ = 830.1597
Calculated $[M\text{-}PF_6]^{2+}$ = 830.1590

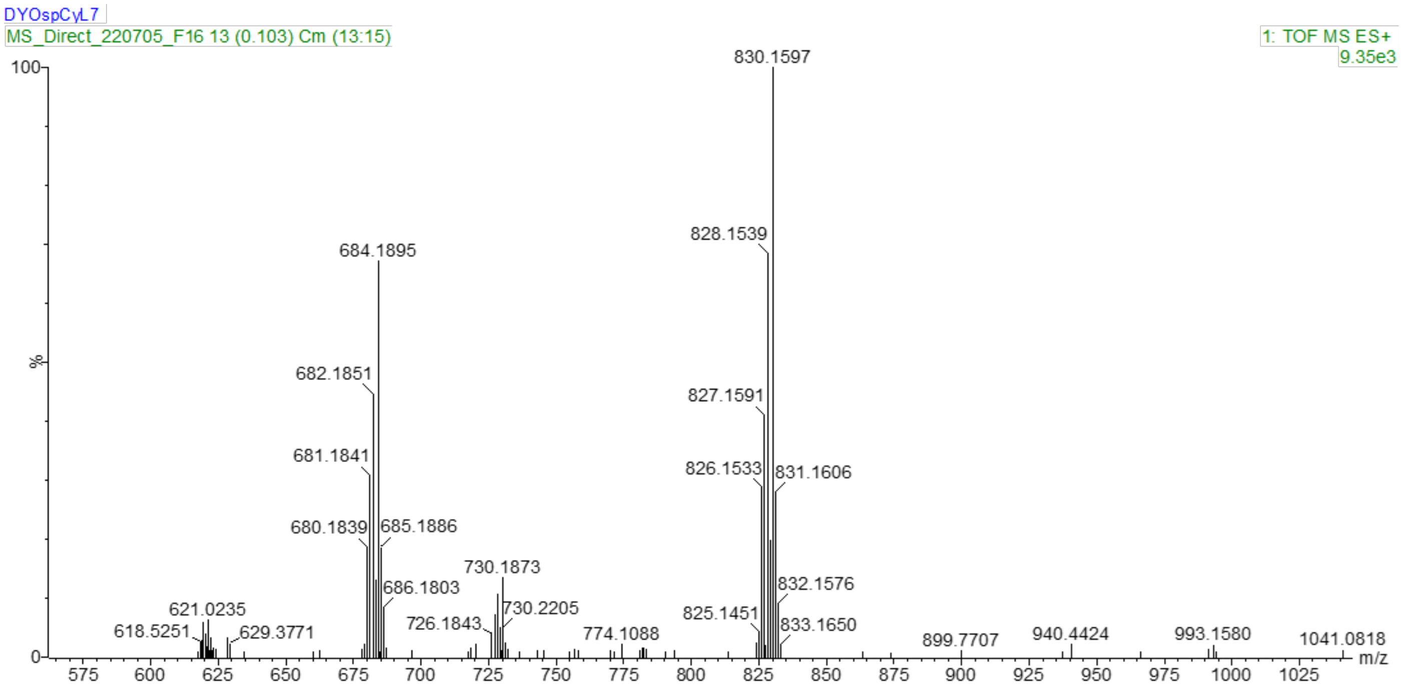

HR-MS of **6civ**

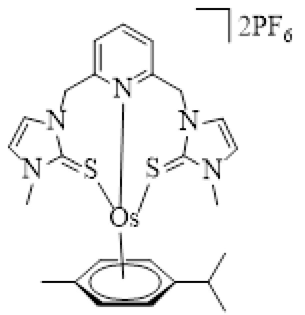

Mol. Wt. [M] = 945.8355
Found: $[M-PF_6 -2CH_3]^{2+}$ = 774.1000
Calculated: $[M-PF_6-2CH_3]^{2+}$ = 774.0964

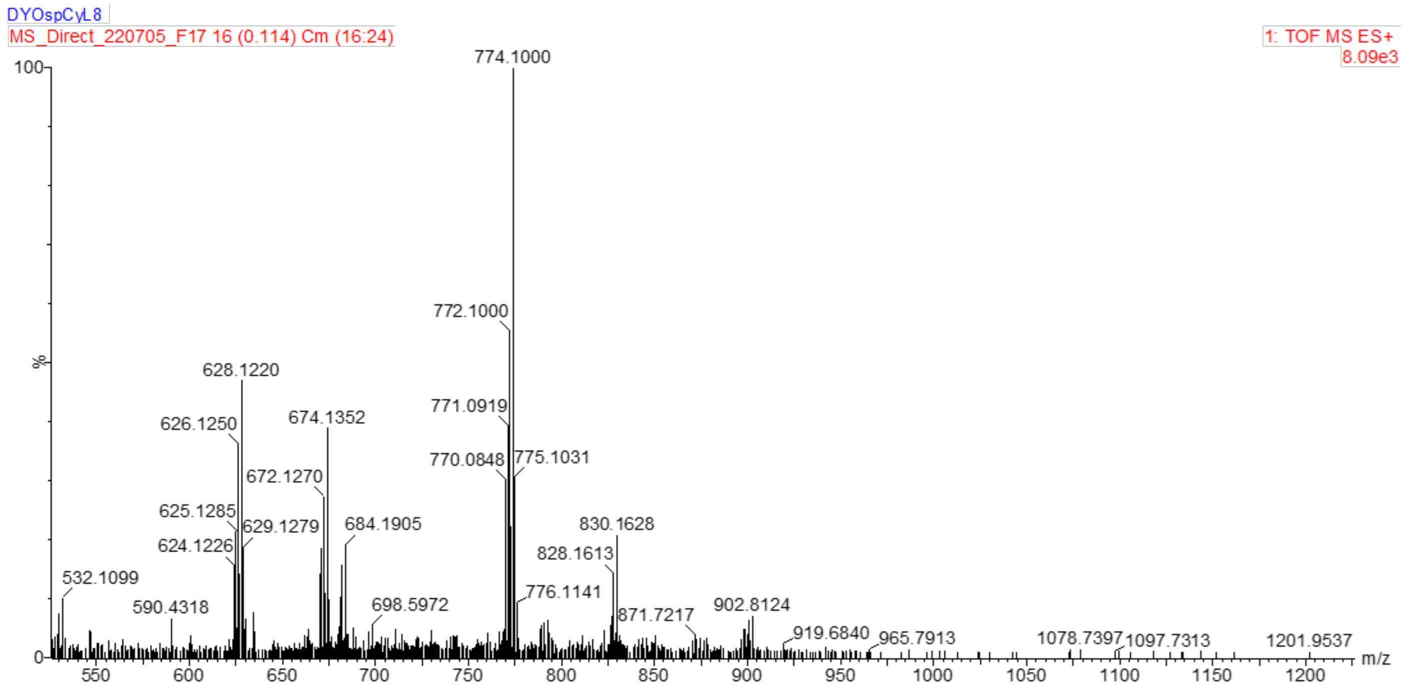

Samples of IC_{50} plots

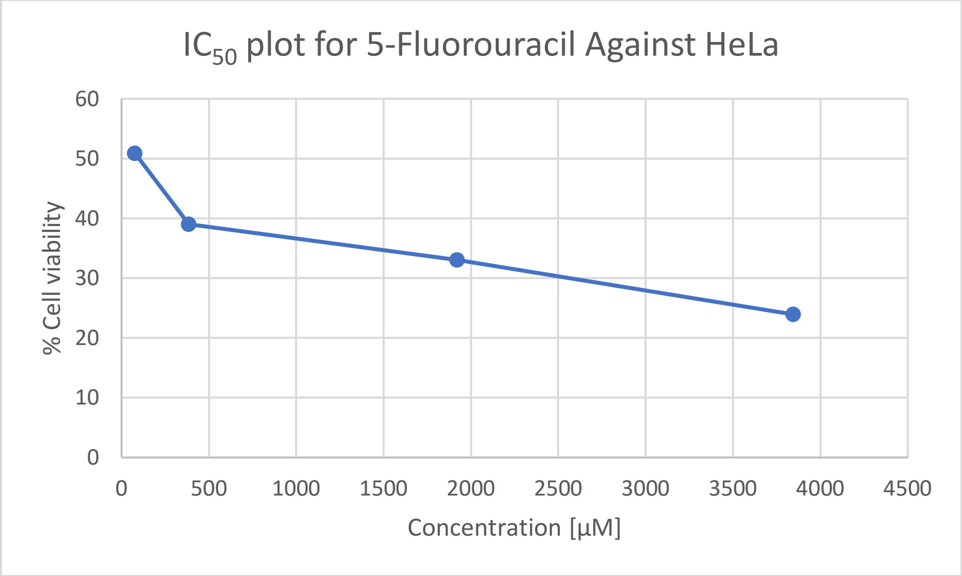

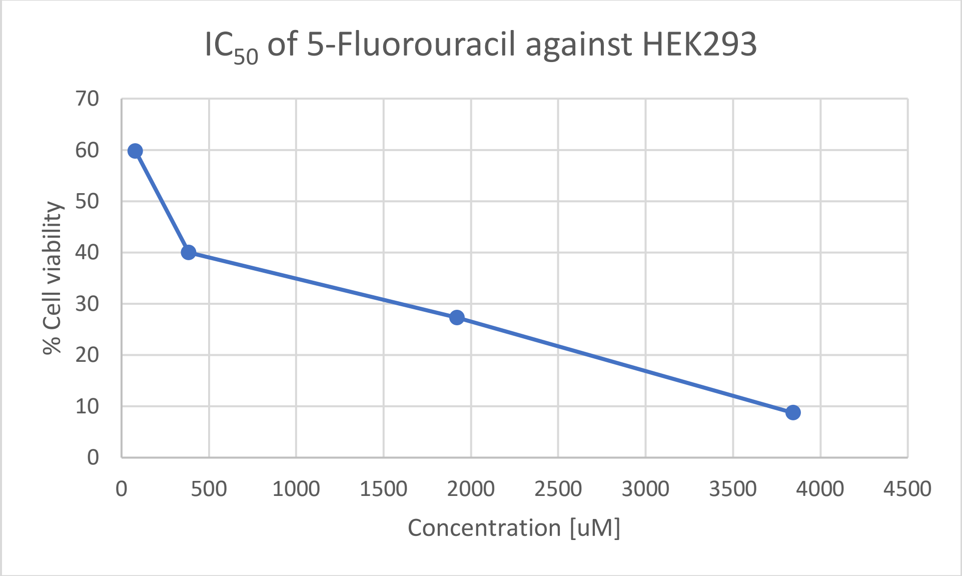

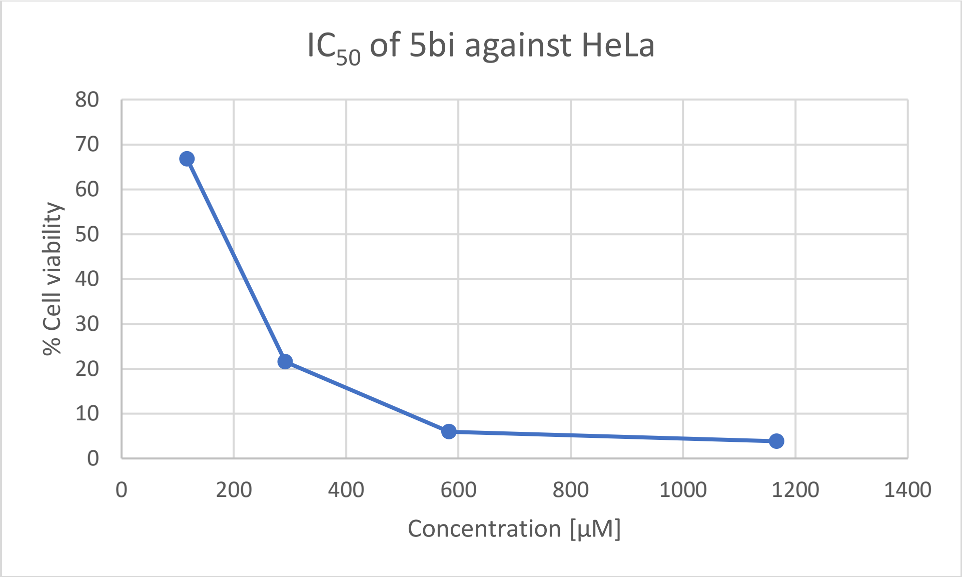
IC$_{50}$ of 5bi against HeLa
% Cell viability
0
10
20
30
40
50
60
70
80
0
200
400
600
800
1000
1200
1400
Concentration [µM]

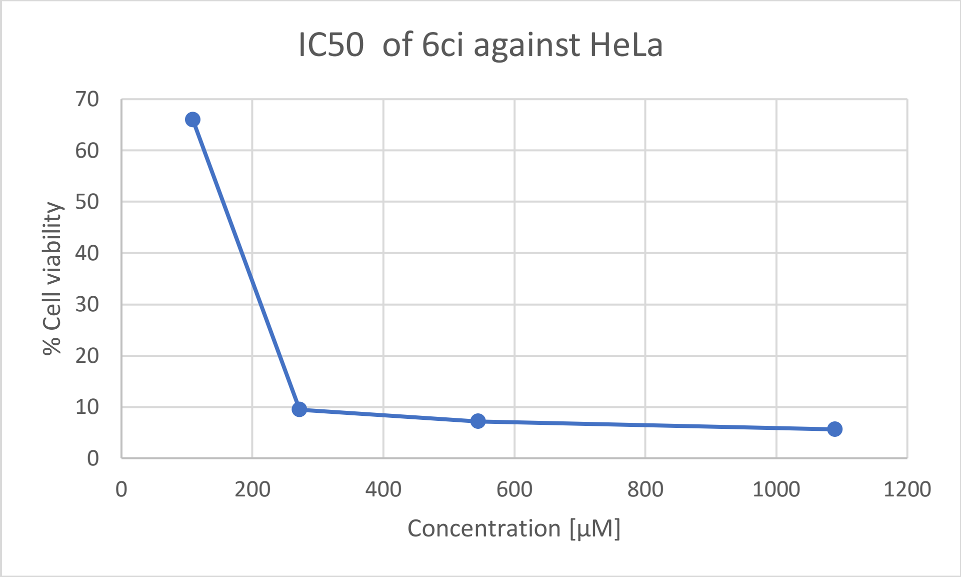
IC50 of 6ci against HeLa
% Cell viability
0
10
20
30
40
50
60
70
0
200
400
600
800
1000
1200
Concentration [µM]

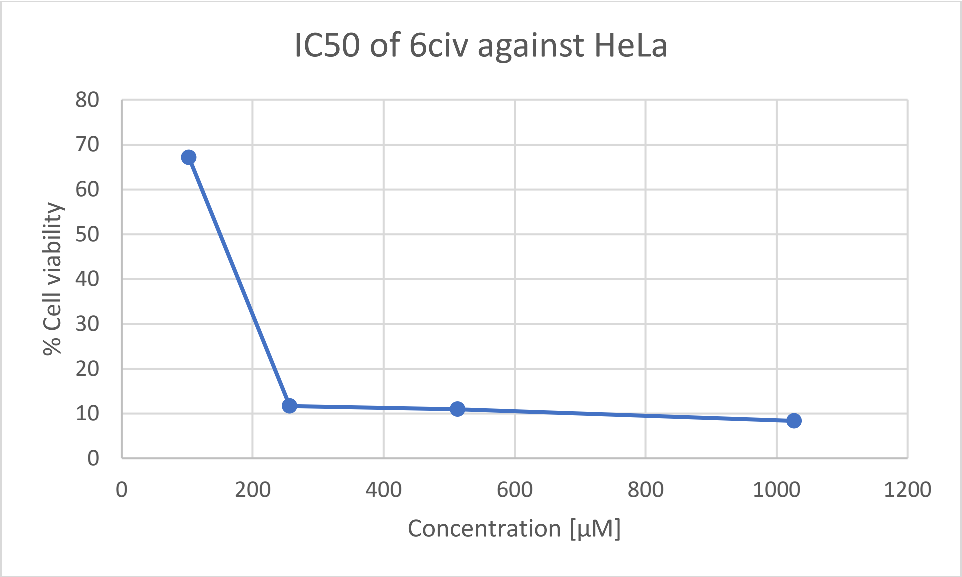
IC50 of 6civ against HeLa
80
70
60
50
40
30
20
10
0
% Cell viability
0
200
400
600
800
1000
1200
Concentration [µM]
